# Global Diversity Lines–A Five-Continent Reference Panel of Sequenced *Drosophila melanogaster* Strains

**DOI:** 10.1534/g3.114.015883

**Published:** 2015-02-11

**Authors:** Jennifer K. Grenier, J. Roman Arguello, Margarida Cardoso Moreira, Srikanth Gottipati, Jaaved Mohammed, Sean R. Hackett, Rachel Boughton, Anthony J. Greenberg, Andrew G. Clark

**Affiliations:** * Department of Molecular Biology and Genetics, Cornell University, Ithaca, New York 14853; † Center for Integrative Genomics, University of Lausanne, Lausanne, Switzerland CH-1015; ‡ Translational Medicine and Think Team, Otsuka Pharmaceutical Development and Commercialization, Inc., Princeton, New Jersey 08540; § Department of Biological Statistics and Computational Biology, Cornell University, Ithaca, New York 14853; ** Quantitative and Computational Biology, Princeton University, Princeton, New Jersey 08544

**Keywords:** *D. melanogaster*, global diversity lines, whole-genome sequences, inversion polymorphism, residual heterozygosity

## Abstract

Reference collections of multiple *Drosophila* lines with accumulating collections of “omics” data have proven especially valuable for the study of population genetics and complex trait genetics. Here we present a description of a resource collection of 84 strains of *Drosophila melanogaster* whose genome sequences were obtained after 12 generations of full-sib inbreeding. The initial rationale for this resource was to foster development of a systems biology platform for modeling metabolic regulation by the use of natural polymorphisms as perturbations. As reference lines, they are amenable to repeated phenotypic measurements, and already a large collection of metabolic traits have been assayed. Another key feature of these strains is their widespread geographic origin, coming from Beijing, Ithaca, Netherlands, Tasmania, and Zimbabwe. After obtaining 12.5× coverage of paired-end Illumina sequence reads, SNP and indel calls were made with the GATK platform. Thorough quality control was enabled by deep sequencing one line to >100×, and single-nucleotide polymorphisms and indels were validated using ddRAD-sequencing as an orthogonal platform. In addition, a series of preliminary population genetic tests were performed with these single-nucleotide polymorphism data for assessment of data quality. We found 83 segregating inversions among the lines, and as expected these were especially abundant in the African sample. We anticipate that this will make a useful addition to the set of reference *D. melanogaster* strains, thanks to its geographic structuring and unusually high level of genetic diversity.

The human commensal dipteran *Drosophila melanogaster* has long played a central role as an experimental genetic model system, as well as a preeminent model for understanding evolutionary and population genetic processes. As one of the first fully sequenced eukaryotic genomes, *D. melanogaster* offers a wealth of genomic and genetic resources, including increasing characterization of natural genetic variation found in extant populations (Langley *et al.* 2012; Mackay *et al.* 2012; Pool *et al.* 2012). Because *Drosophila* biology is being increasingly explored through the use of high-throughput and “omics” approaches, these maps of genetic variation can be placed within a systems biology framework, wherein they are used as natural perturbations for the dissection of complex traits (Ayroles *et al.* 2009; Blair *et al.* 2012; Massouras *et al.* 2012; Breunig *et al.* 2014). The initial motivation to generate these lines was to have wide geographic/climatic provenance to maximize metabolic variation and to use interline differences to explore the genotype−phenotype relation (Jumbo-Lucioni *et al.* 2010; Ghazalpour *et al.* 2014). We intend that these lines will serve as a foundation for advancing ongoing modeling efforts at prediction of metabolic and other complex traits from DNA sequence and other “omics” data sets (Jumbo-Lucioni *et al.* 2010; Eanes 2011; Lavington *et al.* 2014).

Although several population genomic projects have been published recently, they have either been specifically designed for association mapping purposes and thus were generated from a single location and/or from a specific crossing scheme (Mackay *et al.* 2012; King *et al.* 2012; Huang *et al.* 2014), or they were constructed solely for characterizing population genetic variation, without stable lines for functional follow-up studies (Pool *et al.* 2012). A significant difference between our current effort and these previous efforts is that we have aimed to capture variation that exists between geographically diverse populations. As a result, these data will not only serve as an anchor for systems biology approach to complex traits such as metabolic regulation, but also will be generally informative from an evolutionary genetic standpoint. In particular, these data are expected to be well suited for demographic inferences and investigations of local adaptation because of the inclusion of lines from multiple globally distributed populations with distinct evolutionary histories.

In this current article, our intent is to provide a thorough description of the genetic makeup of these lines, which we refer to as the Global Diversity Lines, and detail their creation and our procedures for ensuring data quality. We also aim to highlight salient features of initial analyses characterizing single-nucleotide polymorphisms (SNPs) and small indel variation, as well as evidence for population structure (specific analyses dealing with focused topics will appear in independent articles). We have generated genome sequences for 84 lines to a depth of ~12.5× coverage. We have generated variant calls for SNPs, indels, and large inversions. Independent validation of all classes of variant calls has demonstrated that they are of high quality. A striking feature of inbred *D. melanogaster* genomes, and one that has gained more recent attention, is residual heterozygosity, especially in lines derived from more equatorial populations (*e.g.*, Langley *et al.* 2011). We also observe such heterozygous blocks, and through extensive computational and experimental steps to characterize their nature, find that large regions of residual heterozygosity in inbred lines correlate highly with inversions.

As expected, the African samples are the most diverse for all classes of mutation, but with significant heterogeneity remaining within the other four populations. By sampling from five highly dispersed regions, we anticipate that these lines will be of utility for studies that benefit from maximal genetic diversity, and in answering questions that entail aspects of interpopulation differences that may have been driven in part by local adaptation. The original intent in generating these lines was not for association mapping, but rather was to serve as a reference set of lines of high diversity for building and testing predictive models of metabolic regulation and other complex traits in *Drosophila*. Despite the fact that the lines differ at a large number of nucleotides and insertions/deletions, those differences impose a correlation structure across lines that is informative for a systems biology approach to quantitative models of complex phenotypes. Those modeling papers will appear elsewhere, and it is our intention here to simply provide an introduction to this resource set of lines and to describe the basic attributes of their genetic differences.

## Materials and Methods

We determined and vetted the whole genome sequences of 84 + 1 inbred *Drosophila* lines representing five global populations (Greenberg *et al.* 2010; the “+1” is for line ZW184, recovered in Zimbabwe but appearing to be a very recent migrant, and so is left out of population structure studies). An expanded version of our *Materials and Methods* section is provided as Supporting Information, File S1. To summarize, genomic DNA was extracted from pools of 50 adult females, and we generated paired-end 100 nt Illumina reads at average 12.5× depth for each line (File S1, part 2). Sequence reads were aligned with the Burrows-Wheeler Alignment Tool (BWA, Li and Durbin 2009; File S1, part 3) to the reference *D. melanogaster* genome, and SNPs and small indel genetic variants were called using the Genome Analysis Tool Kit (GATK; McKenna *et al.* 2010; Depristo *et al.* 2011; File S1, part 4). Genetic variant calls were validated in two ways, by resequencing one line to 100× depth and by double-digest restriction-site associated (ddRAD; Peterson *et al.* 2012) resequencing of a consistent subset of the genome for 12 lines, which informed additional filtering to create the final variant call sets (File S1, part 6). We noted that some subregions of the genome in most lines had an unexpectedly high frequency of heterozygous SNP genotypes that validated at high frequency, which we define as “heterozygous blocks” (File S1, part 5). Finally, we investigated the whole-genome sequence dataset for large chromosomal inversions using a custom bioinformatics pipeline (Cardoso-Moreira *et al.* 2012; M. Cardoso-Moreira, J. R. Arguello, D. Riccardi, S. Gottipati, J. K. Grenier, and A. G. Clark, unpublished data) that uses several available tools for genome mapping and structural variation detection [Novoalign (www.novocraft.com), Mosaik (Lee *et al.* 2014), Delly (Rausch *et al.* 2012), and BLAT (Kent 2002); File S1, parts 3 and 10]. Candidate inversions were validated by polymerase chain reaction (PCR) across at least one of the inversion breakpoints, and the genotype of each line was determined by looking for reads supporting the presence of the inversion breakpoint and/or the reference sequence bridging the breakpoint (File S1, part 11). Alignment files and final genetic variant genotypes (vcf files), as well as companion files including 1) het blocks per line, 2) genome callability (File S1, part 9), and 3) regions of genetic identity by descent (IBD; File S1, part 8), are described in Table S1.

Preliminary molecular evolution and population genetic analyses were carried out to provide additional data quality checks, as well as to provide initial broad characterizations of inter- and intrapopulation variation (File S1, parts 13-16). To summarize, our divergence analyses were based on an updated five-species whole-genome alignment that we generated [*D. melanogaster* (dm3), *D. simulans* (droSim2), *D. sechellia* (droSec1), *D. erecta* (droEre2), and *D. yakuba* (droYak2)], within which we include the recently improved *D. simulans* assembly (Hu *et al.* 2013). Population genetic analyses were carried out with the final SNP calls, but were further masked based on variant callability and regions of genetic IBD. Several analyses use an additional SNP subset, referred to as “neutral” SNPs, that fall within small introns or in fourfold degenerate coding positions as determined by our SNPeff annotation (Cingolani *et al.* 2012).

## Results

### Variant discovery

#### Variant calling pipeline:

**Figure 1:**
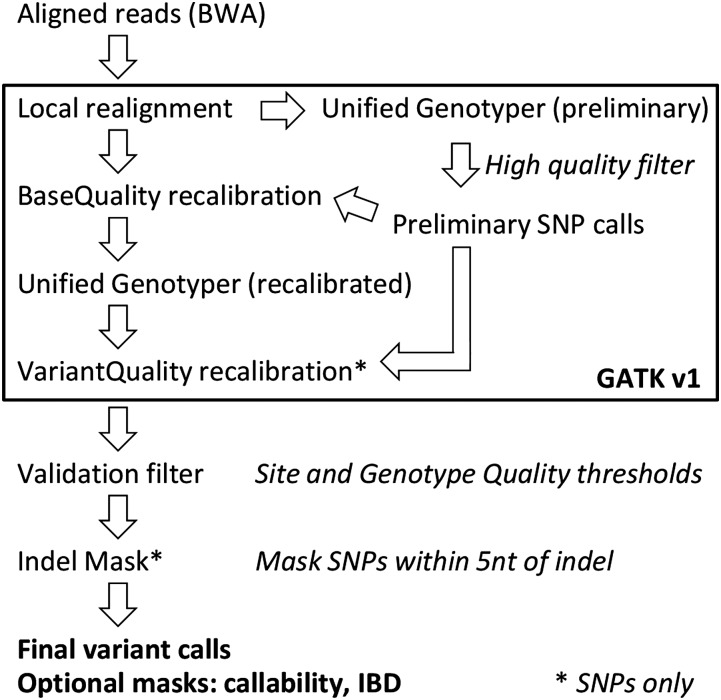
Variant call pipeline. Genetic variants were determined using a pipeline similar to the Genome Analysis Tool Kit (GATK) Best Practices recommendations (https://www.broadinstitute.org/gatk/guide/best-practices) and supplemented with additional filtering steps. First, reads were mapped to the *D. melanogaster* reference genome with the Burrows-Wheeler Alignment Tool (BWA). After merging files for all lines, GATK (version 1) was used to locally realign reads near indels and to create and filter a preliminary set of single-nucleotide polymorphism (SNP) calls. These preliminary SNP calls were used for GATK base quality and variant quality recalibration. After this GATK pipeline, variants were further filtered using variant recalibration site filter and genotype quality filters. SNPs also were filtered when located near an indel call in the same line. See File S1 for details. IBD, identity by descent.

We resequenced the genomes of 84+1 inbred lines of *D. melanogaster* sampled from five globally diverse populations (Greenberg *et al.* 2010) to characterize the genetic variants represented in this collection. Genomes were sequenced at an average 12.5× depth per line (Figure S1 and Table S2), and an average of 92% of the reads from each line mapped to the reference *D. melanogaster* genome. Both SNPs and small indels were called using a pipeline (Figure 1) based on GATK v1 (McKenna *et al.* 2010; Depristo *et al.* 2011). We followed the best practices guidelines of GATK to improve variant calls. Notably, the Base Quality recalibration step reduced the number of euchromatic variant sites (mapped to X, 2L, 2R, 3L, 3R, and 4) by nearly 50%, primarily caused by a decrease in the number of heterozygous SNP calls.

After calling variants with GATK, we immediately observed that the distribution of heterozygous genotype calls was highly nonrandom across the genome of each line. The number of segregating sites within a line was expected to be low because of the 12 generations of sib-pair inbreeding for these lines, but we found contiguous stretches in the genome with a high frequency of heterozygous calls in many lines. These blocks of heterozygosity have also been found in the *Drosophila* Genetic Reference Panel lines albeit to a smaller extent (Table S4 in Mackay *et al.* 2012) and are a known feature of *D. melanogaster* populations, particularly those of African origin (Langley *et al.* 2011). We defined “heterozygous blocks” as genomic regions that harbored an excess of contiguous heterozygous SNP calls. These blocks are seen along all chromosomes, except the X chromosome, where they are relatively rare, and chromosome four, where they are absent. On average there are 6.9 blocks per line. Overall, the average size of a “het block” was 5.4 Mb, though their distribution is bimodal (first mode ~300 kb, second mode ~24 Mb). Although a minority (37%) of the “het blocks” exceeds 2 Mb in length, these large blocks encompass more than 2.7 Gb of the total 2.9 Gb cumulative span, and some “het blocks” extend the length of a full chromosome arm. Notably, the third chromosome and the Zimbabwe populations harbor an excess of these large blocks. The “het block” intervals are available in bed file format (Table S1).

To assess the false-positive rate for the variant calls, and to examine whether additional sites or calls should be filtered out, we validated the SNP and small indel calls in two ways. First, we resequenced a single line (ZW155) to 100× depth. SNPs and small indels were called from aligned reads using a simple read-count ratio to avoid systematic genotyping error. Variant sites were identified as sites with >100 reads for which >10% of reads supported an alternate allele from the reference genome; heterozygous sites were called when two different alleles each had support from >10% of reads. Overall, there was very high agreement between the “10×” GATK and “100×” read-count ratio genotype calls for ZW155 SNPs, greater than 99% for homozygous sites (Figure S2). The agreement for heterozygous SNPs was lower (81%) overall and clearly different within “het blocks” (93%) compared with outside of “het blocks” (30%).

As a second method of validation we generated ddRAD libraries (Peterson *et al.* 2012) for 12 lines. SNPs were called in a similar manner to the “100×” ZW155 validation set using read-count ratios at variant sites with >100× coverage in the ddRAD libraries. The number of validated sites per line was much lower because of the reduced representation of the ddRAD libraries, but the validation rate for SNP homozygous sites was again greater than 99% (Figure S2). The validation of SNP heterozygous sites was initially much lower at 62%, and did not improve within “het blocks.” This discrepancy is most easily reconciled by the fact that reduced representation libraries under-represented one chromosome as a result of variants that alter a restriction site or fragment size (Arnold *et al.* 2013; Davey *et al.* 2013; Gautier *et al.* 2013).

**Figure 2:**
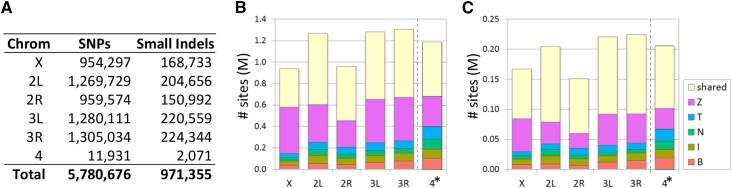
Variant count summary. (A) A total of 5.78 M SNP sites and 971 k small indel sites were discovered in the final panel of 84+1 Global Diversity lines. About half the variant sites per chromosome are shared among more than one population, with the Zimbabwe population contributing the majority of populations-specific variant sites for both (B) single-nucleotide polymorphisms (SNPs) and (C) small indels. The ZW184 line has suspect provenance, and is excluded from B and C (this line is the “+1” in our designation of 84+1 lines). *Chromosome 4 counts are ×10,000 in panels B and C.

We investigated whether the variant quality score (VQS) per site or the genotype quality score (GQ) for individual genotype calls correlated with validation rate. Using the “100×” ZW155 validation set, we found that GQ clearly correlated with validation (Figure S3) but VQS did not (data not shown). Genotype calls with a greater GQ validated at a greater rate. We did find that, following variant quality score recalibration, the sites with the lowest VQS-recalibrated also had lower validation rates, especially heterozygous sites. We used a combination of GQ score, VQS recalibrated-flag, and genotype to filter out classes of genotype calls with validation rates below 90%, including all heterozygous calls outside of “het blocks.” This filtering reduced the number of nonreference SNP genotypes by 12% and the number of variant SNP sites by 5%. Finally, we masked SNP genotype calls if the SNP was within 5 nt of an indel call in the same line. There are more than 5.75 M euchromatic SNPs in the final set of variant sites (Figure 2 and Table S3), with 97% of genotypes called across 84+1 lines.

To validate small indel calls, we used the same “100×” ZW155 deep resequencing data (Figure S4). Coverage of indels in the ddRAD dataset was too low and yielded uncertain representation of heterozygous sites to use for validation. Similar to the SNP calls, we used a combination of GQ score and genotype to filter out classes of genotype calls with validation rates below 75%, including all heterozygous calls outside of “het blocks.” A lower validation threshold was used for small indels because the only heterozygous calls retained (GQ = 99, inside “het block”) had a validation rate of ~75%, which set a maximum for homozygous calls as well to retain the same density of variant genotypes across lines. In total we identified nearly 1M euchromatic small indels (Figure 2 and Table S3), with 80% of genotypes called across 84+1 lines.

The impact of the variant call pipeline was quite different between SNPs and small indels, as well as within and outside of “het blocks.” The base quality score recalibration reduced the number of heterozygous calls similarly across all chromosomes and lines such that “het blocks” retained about half the number of preliminary heterozygous calls whereas regions outside of “het blocks” retained very few heterozygous genotype calls with lower GQ scores. In contrast, base quality score recalibration had minimal effect on homozygous calls. The number of heterozygous calls for a given line and chromosome is variable, with some lines having a comparable number of heterozygous and homozygous calls and others having very few heterozygous calls (Figure S5). These patterns indicate the presence or absence, respectively, of large “het blocks.” The pattern for small indels is similar, including the reduction in heterozygous calls across all lines in the base recalibration step and the prevalence of heterozygous calls for certain lines and chromosomes indicating the presence of ‘het blocks’, although the filtering step reduced the number of calls more significantly than for SNPs (Figure S6).

#### Gene annotation:

**Figure 3:**
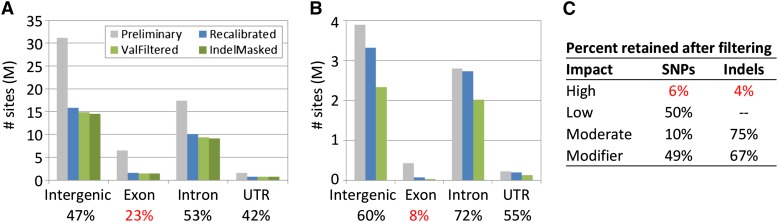
Gene annotation. The number of variant sites falling within exons was disproportionately reduced during the variant call pipeline for both (A) single-nucleotide polymorphisms (SNPs) and (B) small indels compared with variant site locations, particularly due to the GATK base quality recalibration step. The percent of variant sites retained after filtering is shown below the gene annotation category. (C) Similarly, SNP and small indel sites annotated as high impact were disproportionately reduced during the variant call pipeline compared to lower impact sites.

We next investigated the distribution of variant calls with respect to gene annotation. Variants with high predicted impact and located in exons were least likely to be retained in the variant call pipeline (Figure 3), indicating that as call quality increased due to recalibration and filtering, the false-positive rate of high impact sites decreased. Additionally, we observe a clear mod-3 pattern in small indels located in coding regions, which is magnified by the recalibration and filtering steps (Figure S7). These patterns of change in the distribution of variant annotations indicate the improved quality of the variant calls at each step of the pipeline.

#### Genomic regions inferred to be IBD:

Investigation of genetic IBD revealed relatively few genomic regions containing signatures of recent common ancestry. As observed previously in *D. melanogaster* population genomic data (Langley *et al.* 2012), most regions possessing candidate IBD stretches were restricted to the lowly recombining regions of pericentromeric regions. After we excluded these regions, 30 chromosomal stretches that were shared between single pairs of individuals within the same population remained. The median size of these stretches was estimated to be 5.64 Mb, and they were observed on all chromosome arms other than the 4th. The 30 genomic regions found IBD in a pair of lines are available in bed file format (Table S1).

#### Large inversions are common and are associated with heterozygous blocks:

The identification of genomic regions with segregating variation within lines (“het blocks”) despite 12 generations of sib-pair inbreeding suggests that this heterozygosity is being actively maintained. One explanation would be the presence of large chromosomal inversions, known to be common in *D. melanogaster*—especially in equatorial populations (Lemeunier and Aulard 1992). Large inversions protect regions of the genome from recombination and, when recessive deleterious alleles are present between the inversion breakpoints on both segregating chromosomes, can prevent complete inbreeding of lab stocks. The hypothesis that blocks of heterozygosity are the result of the presence of recessive lethal alleles and the presence of large inversions leads to a clear and testable prediction: heterozygous blocks should be associated with the presence of *heterozygous* inversions.

We developed a bioinformatic pipeline to detect candidate inversions from the mapped whole-genome sequencing reads. Candidate inversions were assayed by PCR across the predicted breakpoints and sequence-verified; this validation process indicated a high false-positive rate for inversion prediction, but also did confirm the presence of at least 10 large inversions across the Global Diversity Lines.

We independently identified and validated all of the eight large *D. melanogaster* inversions with known molecular breakpoints (Wesley and Eanes 1994; Andolfatto *et al.* 1999; Matzkin *et al.* 2005; Corbett-Detig *et al.* 2012), including four common and cosmopolitan inversions (In(2L)t, In(2R)NS, In(3L)P, and In(3R)P); two rare cosmopolitan inversions (In(3R)K and In(3R)Mo); and two recurrent endemic inversions (In(X)A and In(X)Be); see File S2 for breakpoint sequences. We identified at least one line carrying each of the known large inversions; most inversions were found in lines from more than one population, except the rarer X-linked inversions which were each found in a single African line (Table S4).

We also identified the molecular breakpoints of two additional inversions (File S2). One of the inversions matches well the cytogenetic limits for In(3L)62D:68A ‘Ok,’ which has been described as a recurrent endemic inversion in Africa (Lemeunier and Aulard 1992), a fact that agrees well with the exclusive presence of this inversion in the Zimbabwe population. The other inversion we identified on chromosome 3R does not match perfectly the cytogenetic limits of previously described inversions but is located in the proximity of several candidates (Lemeunier and Aulard 1992).

For most large inversions, a line often had reads indicating both the presence of the inversion chromosome and the reference chromosome, indicating both chromosomes are segregating in the line. In total we identified 83 instances of heterozygous inversions segregating in our lines and 10 instances of homozygous inversions (Table 1 and Table S4). Of the 83 heterozygous inversions, 81 (98%) are located within heterozygous blocks (Table 1 and Figure S8); none of the 10 homozygous inversions are associated with heterozygous blocks. This strongly supports the hypothesis that the lingering blocks of heterozygosity result from the presence of recessive deleterious alleles in linkage with inversions.

**Table 1:** Large inversions explain many regions of heterozygosity in the Global Diversity Lines

Inversion	Breakpoint Source	Homozygous Inversions		Heterozygous Inversions	
		All	In HetBlock	All	In HetBlock
In(2L)t	*^a^*	3	0	20	20 (100%)
In(2R)NS	*^a^*	0	–	9	7 (78%)
In(3L)P	*^a^**^,^**^b^*	3	0	13	13 (100%)
**In(3L)62D;68A**	*^a^*	0	–	10	10 (100%)
In(3R)Mo	*^b^*	2	0	5	5 (100%)
**In(3R)13-72**	*^a^*	1	0	0	–
In(3R)K	*^a^**^,^**^b^*	0	–	5	5 (100%)
In(3R)P	*^c^*	0	–	20	20 (100%)
In(X)Be	*^a^**^,^**^b^*	1	0	0	–
X(A)	*^b^*	0	–	1	1 (100%)

Bold: Molecular breakpoint first characterized in this study.

a This study.

b Corbett-Detig *et al.* 2012.

c Genbank.

The explanatory power of the seven inversions found as heterozygous varies between populations. In the Zimbabwe population these seven inversions are sufficient to explain 90% of large heterozygous blocks. Outside of Africa, these seven inversions account for a smaller percentage of heterozygous blocks: 58% in the Tasmanian population, 57% in the Beijing population, 43% in the Netherlands population, and 32% in the Ithaca population. That only seven inversions could account for such a large fraction of heterozygous blocks (57% across the whole dataset) is quite surprising. After all, there are hundreds of inversions segregating in *D. melanogaster* (Lemeunier and Aulard 1992), including four rare cosmopolitan inversions and eight endemic recurrent inversions that do not have characterized molecular breakpoints. Our work suggests that if all inversions segregating in our set of lines were identified, they could explain all large blocks of heterozygosity found in our lines. However, these inversions may be difficult to identify using short reads, as careful investigation of the remaining candidate inversions called by our bioinformatics pipeline do not align with a large fraction of unexplained blocks of heterozygosity.

The presence of large inversions and associated heterozygous blocks in the inbred Global Diversity Lines implies that most individual flies in the stock may be heterozygous, because homozygous flies may have low chance of survival or be infertile. We developed PCR-based genotyping assays for inversions and for linked SNPs and confirmed that individual fly genotypes are typically heterozygous within inversion-associated heterozygous blocks (data not shown). Furthermore, to directly test whether heterozygous blocks contain recessive deleterious alleles, we designed a genetic approach to force selected chromosomes to be homozygous (see File S1 part 12). For five of six chromosomes tested, we found that homozygotes for either of the chromosomes segregating in the original stock were significantly underrepresented or entirely absent relative to heterozygous sibs.

There was one exception to the observation that inversions are heterozygous in individual flies and do not support a healthy homozygous stock. We found that one inversion, In(3L)62D:68A, is often homozygous in Zimbabwe line ZW155 (1 of 5 males, 4 of 10 females), and that homozygous flies are fertile. The noninverted chromosome appears to harbor a recessive lethal allele, as it was never found homozygous in individual flies from this line. Furthermore, the inversion chromosome from this line generated close to the expected ratio of homozygous viable progeny in the genetic test, although derived homozygous stocks appear weak after a few generations. Surprisingly, given that flies homozygous for the In(3L)62D:68A chromosome in the ZW155 line appear viable and fertile, the In(3L)62D:68A inversion was sampled 10 times in the Zimbabwe lines and is never found homozygous despite 12 generations of sib-pair inbreeding. Thus we suspect that the In(3L)62D:68A inversion chromosome has sufficiently low fitness to avoid homozygosity.

### Population genetic-based tests of the data

The quality of the sequence data indicates that it is suitable for molecular population genetic analyses. Our initial work highlights strong population differences among the five populations, as well as differences between autosomes and the X chromosome within populations. Patterns of nucleotide diversity and LD are consistent with Zimbabwe having the largest effective population size, while non-African populations are significantly less diverse and more similar to one another than to Zimbabwe. Notable differences in allele frequency exist among combinations of populations, but also at putatively functional sites that are monomorphic in all but one population, where a high frequency derived allele is found. In a companion paper we will investigate questions related to neutral and selective forces leading to population differentiation, as well as autosomal and sex chromosome differences.

#### Population structure:

**Figure 4:**
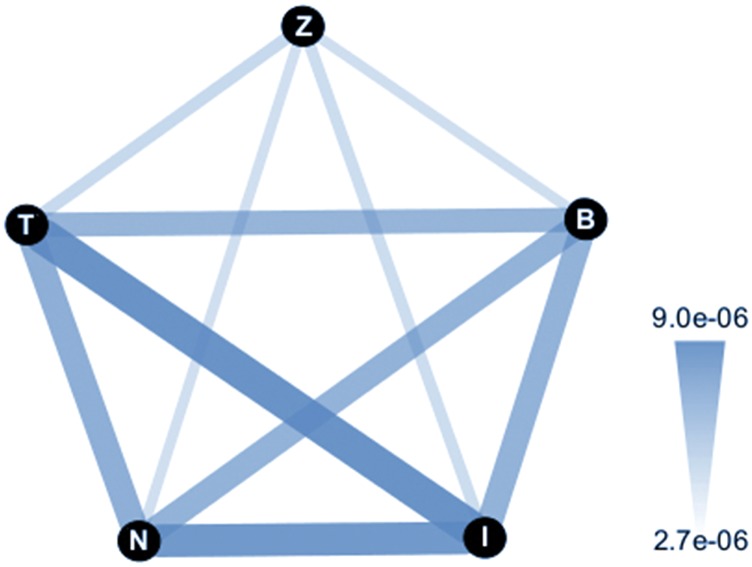
Population distance network for the five populations as measured by genome-wide *F_ST_* . Nodes represent each of the five populations (B: Beijing; I: Ithaca; N: Netherlands; T: Tasmania; Z: Zimbabwe), with edges representing the estimated distances measured by *F_ST_* between all pairs of populations. Increased edge width and transparency corresponds with increased migration/gene flow (and decreased *F_ST_* /differentiation).

As a first look into the extent of population structure between samples, we calculated genome-wide *F_ST_* using the SNP data (Figure 4). Consistent with an out of African migration (Begun and Aquadro 1993; Glinka *et al.* 2003; Haddrill *et al.* 2005; Ometto *et al.* 2005; Li and Stephan 2006; Thornton and Andolfatto 2006; Laurent *et al.* 2011), Zimbabwe is the most differentiated population, as can be seen by the narrow and more transparent edges in the network (Figure 4; average *F_ST_* autosome: 0.08; X chromosome: 0.139), with the four non-African populations more closely related to each other. These levels of *F_ST_* are in general agreement with reports from smaller datasets from the same populations (or proximal geographically). Interestingly, after Zimbabwe, Beijing is the next most differentiated. A previous survey also showed that other Asian strains were among the more differentiated within world-wide samples (Schlötterer *et al.* 2006). Although the average *F_ST_* for Beijing is not as large as some of these other Asian estimates (average *F_ST_* autosome: 0.05; X chromosome: 0.08), they provide additional evidence that Asia may harbor some of the more divergent *D. melanogaster* populations globally.

**Figure 5:**
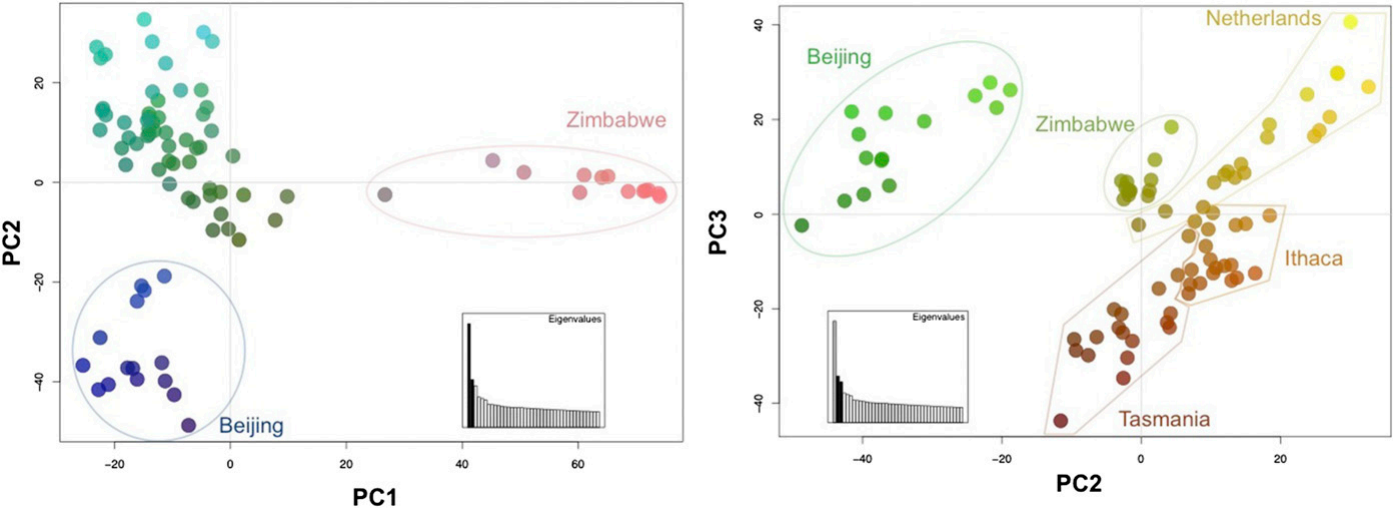
Population structure revealed by principal on autosomal “neutral” sites. Principal component clustering plots demonstrate the genetic structure present among the five populations. Left panel displays the strong separation of the Zimbabwe and Beijing populations from the remaining three populations based on the first two principal components. The right panel displays further separation of the Netherland, Tasmania, and Ithaca populations based on principal components two and three. Although less structured, clear separation is still observed for these latter three populations.

Principal component analysis performed on the genome-wide “neutral” sites, which should best reflect demographic processes, reveals notable population structure among all populations (Figure 5). Plotting the first two principal components cleanly separates not only Zimbabwe but also Beijing. However one line annotated as having an African origin (ZW184) does not group with the other Zimbabwe lines; we have excluded this line from subsequent population genetic analyses because its Zimbabwe origin is suspect, although it remains included in the global set of lines for modeling and mapping applications. Plotting the additional principal components (or removing the Zimbabwe lines, data not shown) reveals that even the Ithaca, Tasmania, and Netherlands population samples can be clearly separated.

#### Combined site frequency spectra (SFS):

**Figure 6:**
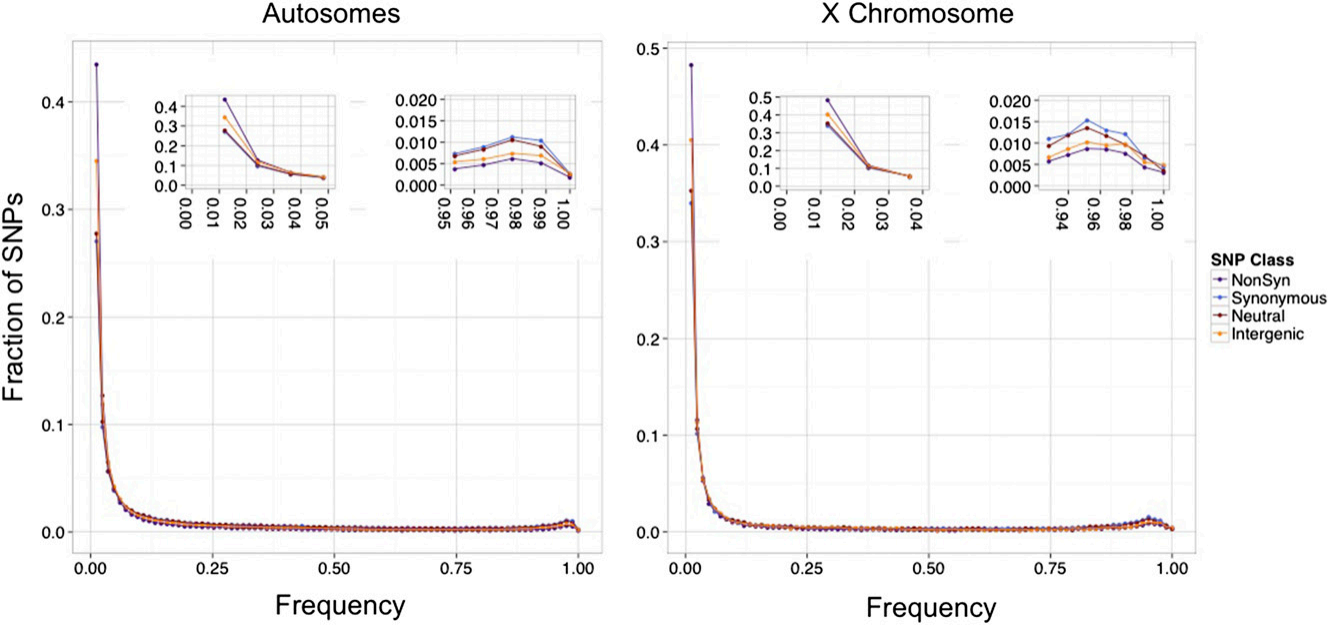
Site frequency spectra for four single-nucleotide polymorphisms (SNP) classes. Unfolded (polarized for ancestral state) SFS are shown for the four SNP classes separated for the autosomes (left) and the X chromosome (right). Insets display the two extreme ends of the distributions where differences between the SNP classes are most pronounced.

Another data quality check examined the unfolded SFS for four SNP classes combined across the full dataset and partitioned into an X chromosome set and an autosomal set: (1) nonsynonymous, (2) synonymous, but not fourfold degenerate, (3) intergenic, and (4) a “neutral” class comprised of fourfold degenerate and small intronic SNPs (Figure 6). The number of SNPs (~167,000) was balanced to match the limiting counts from the ‘neutral’ set.

The shapes of the SFS are consistent with the validation results (shown previously), showing the expected selection-driven enrichment of low frequency SNPs and characteristic decrease in abundance among more frequent variants. Among the rarest variants, there is a roughly 20% excess abundance of nonsynonymous SNPs (compared with synonymous SNPs), consistent with the role of purifying selection working to eliminate deleterious mutations from the population. The right tail of the SFS reflects the abundance of high frequency derived SNPs, and the excess (compared to a neutral expectation) seen for all classes of SNPs may reflect SNPs that have risen to high frequency either neutrally or due to selection (either because of direct selection or linkage to selected variants), motivating a more complete follow-up analysis.

#### Population-specific SFS and private variants:

Partitioning the SFS into population-specific SFS further highlights population differences (Figure S9). For example, the larger effective population size of the Zimbabwe sample is apparent caused by the overall greater number of variant sites, both for the autosomes and the X chromosome. In particular, most of the contribution in the singleton class is provided by the Zimbabwe lines (Figure S9). In addition, the abundance of high frequency−derived alleles varies depending on both the class of site and the population being examined. These differences are not unexpected given the complex interactions of demographic and selective forces, and are currently being investigated.

**Figure 7:**
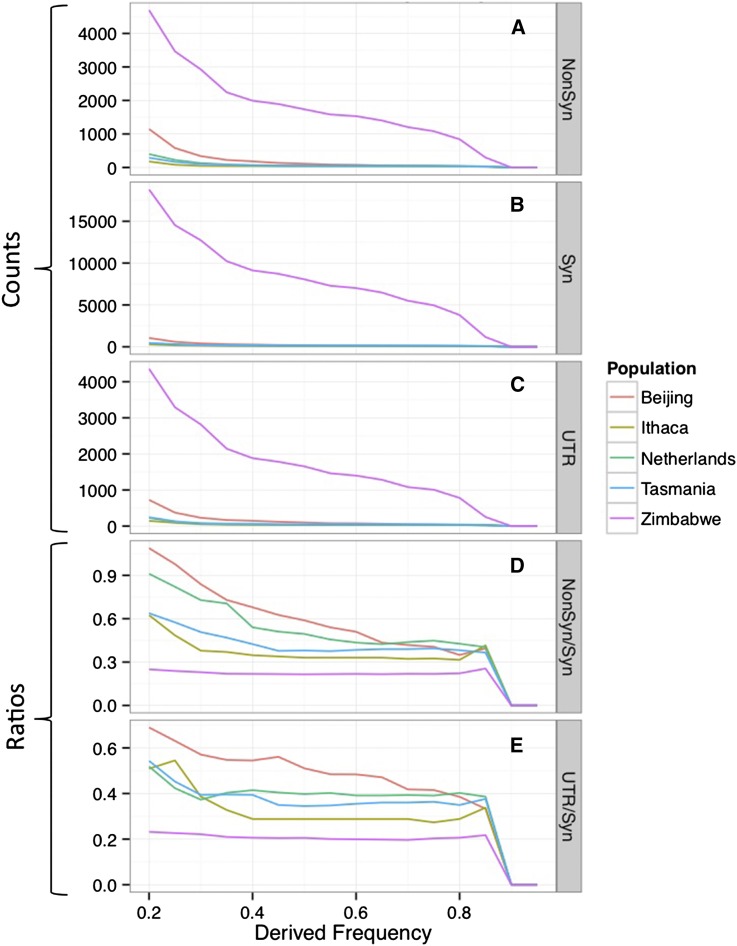
Summary of the Number of Potentially Functional Population-specific single-nucleotide polymorphisms. High-frequency derived allele counts for genic (A and B) and UTR regions (C) at frequencies between 20% and 100% are plotted for five populations. All sites in the plot are based on the IBD and callability-masked SNP dataset, have <20% missing data, and carry ≥70% posterior probability for their ancestral state. Panels (D) and (E) attempt to normalize the nonsynonymous and untranslated region (UTR) counts by showing the ratio to the putatively more neutral synonymous counts. IBD, identity by descent.

A major interest of the field is lineage-specific differentiation and the extent to which such differences might be driven by local adaptation. As a first look into this question we extracted all SNPs that were private to a single population and that also had a relatively high allele frequency (≥20%). Figure 7, A−C plots the counts for these alleles stratified by allele frequency bins. The finding of thousands of population-specific nonsynonymous, synonymous and UTR SNPs is highly unlikely, given a simple island model with the observed *F_ST_* (these counts ranged from 1387 for Ithaca to 52,079 for Zimbabwe). These initial observations suggest a role for natural selection-driving patterns of differentiation and motivate a more thorough analysis of local adaptation detectable with these data.

#### Population-specific diversity and linkage disequilibrium (LD) estimates:

**Figure 8:**
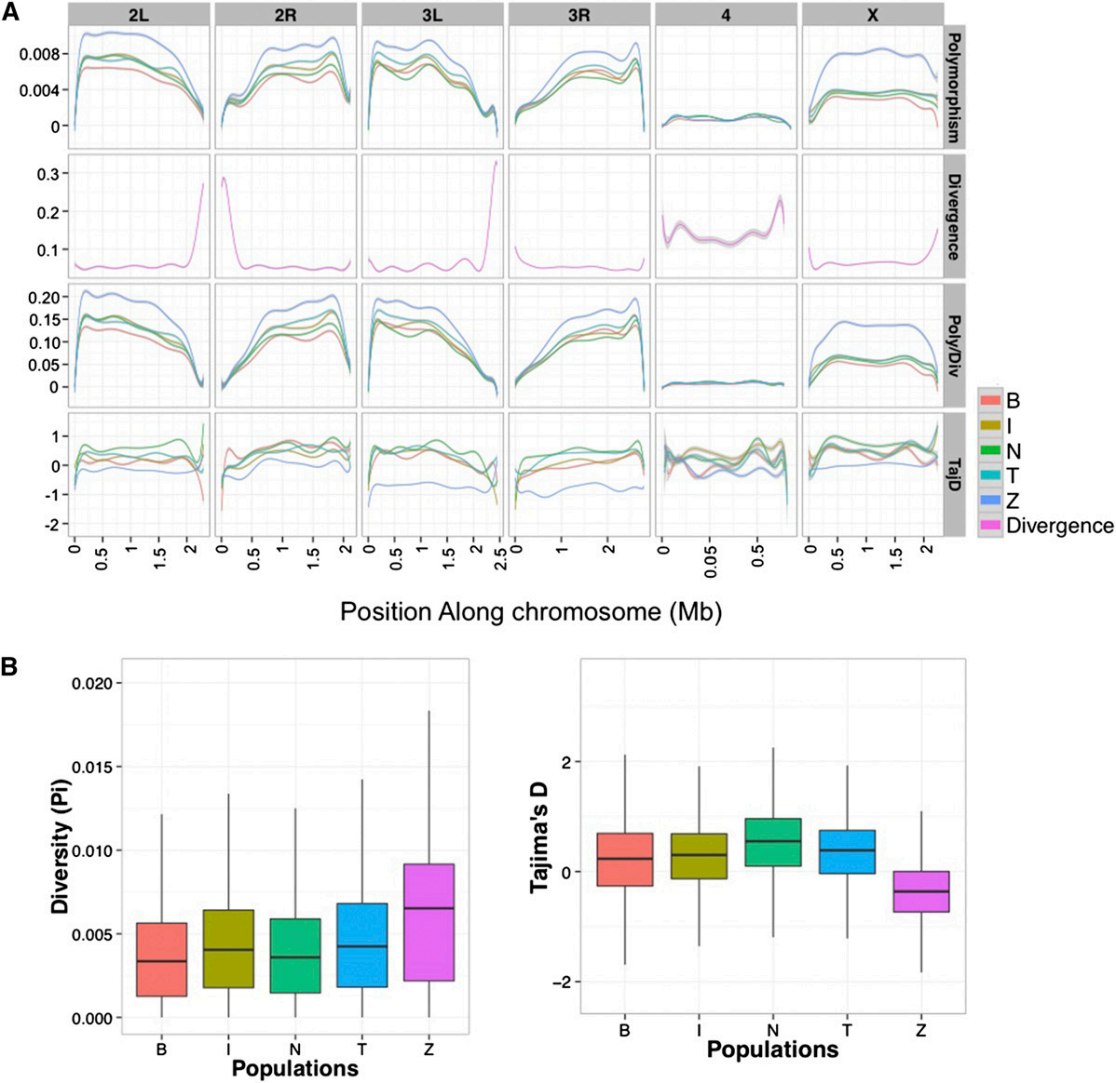
Genome-wide diversity summaries. (A) Sliding window plots display summary statistics for the single-nucleotide polymorphism data along all four chromosome arms. The window size for chromosomes other than the 4th are 10 kb, with stride length equal to 5 kb; Windows for the small 4th chromosome are 500 bp with stride length equal 250 bp. Population abbreviations are: B = Beijing, I = Ithaca, N = Netherlands, T = Tasmania, and Z = Zimbabwe. “Polymorphism” refers to the average pairwise nucleotide difference (π), “Poly/Div” refers to the polymorphism divided by divergence, and “TajD” refers to Tajimas *D*. (B) Boxplots summarize the diversity data shown in (A) for all chromosomes. Heterogeneity in genome-wide nucleotide diversity (π) is observed across all populations (one-way analysis of variance, *F*_4, 146050_ = 2583.8, *P* << 0.01; all pairwise comparisons contribute significantly to this result {all Tukey post-hoc comparisons *P* < < 0.01}) . The most notable difference is between the non-African populations’ and the Zimbabwe’s X chromosomes. Non-African X chromosomes display 37–46% less diversity within the X chromosome as compared with the normally recombining autosomes (Wilcoxon *P* << 0.01), whereas the Zimbabwe sample has a slight excess (2%). As a result, the autosomal-chromosome X comparison for Zimbabwe is nominally significant in the opposite direction (Wilcoxon *P* = 0.011). Within population comparisons of Tajima’s *D* values (*D*) on the X *vs.* autosomes are also significantly different (Wilcoxon *P* << 0.01 for all contrasts), as are most comparisons across populations (one-sample Wilcoxon *P* << 0.01). Tukey post-hoc comparisons indicate that only the Ithaca-Beijing X chromosome and the Tasmania-Ithaca autosome comparisons are nonsignificant (*P* > 0.05). Zimbabwe stands out as having a significantly negative mean autosomal *D*, whereas for the X chromosome it is not different from zero (Wilcoxon *P* > 0.05).

As indicated by the SFS previously, significant heterogeneity in genome-wide nucleotide diversity is observed across all populations (Figure 8). Median genome-wide diversity levels (π) range from ~0.3% (Beijing) to ~0.6% (Zimbabwe); Watterson’s θ ranges from ~0.03% to ~0.07%. Consistent with previous studies, broad-scale patterns of within-genome diversity covary with local recombination intensity and display reduced diversity near the telomeres and centromeres (Figure 8A). In addition, the X chromosomes of all populations—except for Zimbabwe —display a marked reduction in diversity levels as compared with the normally recombining autosomes (excluding chromosome 4).

Within-population comparisons of Tajima’s *D* values (*D*) on the X *vs.* autosomes also consistently demonstrated significant differences between the chromosomes (Figure 8). Each of the four non-African populations has a significantly positive mean *D* value for both the autosomes and the X chromosome, consistent with past bottlenecks. Zimbabwe stands out as having significantly negative mean autosomal *D* values, and a mean *D* for chromosome X that is not different from zero, possibly indicating a recent population expansion (Glinka *et al.* 2003; Ometto *et al.* 2005). Historical population size differences can also be observed in differences in linkage disequilibrium (LD) over physical distance (Figure S10; McVean 2002): Zimbabwe has significantly less LD than the other populations (excluding chromosome 4), with Beijing and Netherlands possessing the highest levels of LD and Ithaca and Tasmania displaying intermediate levels. For all samples, the bulk of LD is lost within 100-150 bps, which is consistent with previous observations (*i.e.*, Huang *et al.* 2014).

The aforementioned collection of preliminary population genetic analysis serve to show that the collection of Global Diversity Lines have genetic attributes consistent with known history of *Drosophila* phylogeography, and they serve as a reassuring parity check attesting to the accuracy of SNP genotype calling. More detailed analyses aimed at inferring demographic parameters for this data set will be submitted as an independent manuscript elsewhere.

## Discussion

An excellent starting point for developing a “systems biology”–based analysis of natural variation is to generate a reference set of inbred fully sequenced lines having maximal genetic diversity (Ayroles *et al.* 2009). Toward this end, and to advance quantitative approaches relating genomic and phenotypic variation, we have characterized genomic variation among *D. melanogaster* sampled from five disparate populations. Most immediately, these data will provide a foundation for modeling complex traits such as metabolic regulation, a dynamic phenotype that varies between these populations (Greenberg *et al.* 2010, 2011; Scheitz *et al.* 2013), and which is amendable to increasingly numerous and sensitive “omic” assays. Additionally, this collection of variants—and the available inbred lines—will broadly benefit a wide range of screens and assays seeking to understand geographically localized adaptations. For example, the whole-genome sequencing dataset has also been used to characterize *Wolbachia* and mitochondrial haplotypes (Early and Clark 2013), repeat sequences (Wei *et al.* 2014), and immune gene evolution (A. Early, J. R. Arguello, M. Cardoso-Moreira, S. Gottipati, J. K. Grenier, and A. G. Clark, unpublished data) in the Global Diversity Lines.

Extensive effort has been invested into variant calling and empirical validation. The validation procedures that we have followed, involving the combination of orthogonally generated data, revealed notable context-dependent differences that do not allow for simple genome-wide variant call thresholding (*i.e.*, within and outside of heterozygous blocks, or for different genotype calls). In total this new data set is comprised of ~5.78 M SNPs and nearly 1 M small indels, providing a rich reservoir of naturally occurring variants with multiple mutations in every gene.

The general agreement between our initial population genetic analysis and previous population genomic studies (Begun and Aquadro 1993; Langley *et al.* 2012) would seem to confirm that our data are of sufficient quality to allow for drawing population genetic inferences. The pronounced population structure among shared variants, particularly on the X chromosome, highlights the necessity of accounting for the underlying demographic processes that have led to genetic and phenotypic differences among these lines. As expected, our African sample carries the signatures of being ancestral to all of the non-African samples (high diversity and low LD; Begun and Aquadro 1993; Glinka *et al.* 2003; Haddrill *et al.* 2005; Ometto *et al.* 2005; Li and Stephan 2006; Thornton and Andolfatto 2006; Laurent *et al.* 2011). In addition, Zimbabwe carries an excess of singletons (highlighted by the negative Tajima’s *D* values), which has been observed previously (Glinka *et al.* 2003; Ometto *et al.* 2005). Regarding the non-African samples, the Asian sample was observed as the second most divergent population. Although there had previously been suspicion of an independent migration from Africa to Asia (David *et al.* 1976), more recent data and demographic modeling argued against it (Baudry *et al.* 2004; Schlötterer *et al.* 2006; Laurent *et al.* 2011). In line with a unique out-of-Africa origin, the Beijing sample is more closely related to the other non-African samples than to Zimbabwe. The range of diversity estimates for non-African genomes (0–1.5%), with a median below 0.5% is also consistent with previous reports (Ometto *et al.* 2005; Laurent *et al.* 2011; Langley *et al.* 2012). However, we tend to see somewhat elevated intermediate allele frequencies (positive Tajima’s D) compared to some reports, possibly because this is the first global-scale genome-wide estimate, or possibly because of the laboratory inbreeding. But regardless of this issue, it is very clear that we find numerous population-specific variants—particularly derived alleles that are segregating at high frequency—highlighting the exciting opportunities to investigate cases of population-specific differences and local adaptation.

Our experimental and bioinformatics investigation of the relationship between heterozygous blocks and inversions has provided an important focus on the high frequency and chromosome-scale impact of large inversions in *D. melanogaster*. The *de novo* generation and subsequent tolerance of inversions is increasingly recognized to be a major feature of this species’ genome (Schlötterer *et al.* 2006; Corbett-Detig and Hartl 2012; Langley *et al.* 2012). The basic biology underlying this phenomenon is of fundamental interest but is also relevant for mapping and quantitative genetic modeling efforts within this species. For example, it is important to determine what portion of phenotypic variance in adaptive traits is mediated by genetic variation captured in these inversions. To fully understand the impact of the past demographic and selection histories of *D. melanogaster* in influencing the genetic basis of complex traits, it will be essential to characterize the population dynamics of inversions, including the age, geographic distribution, and impact of individual inversions and the modes of selection that maintain and modulate their frequency in natural populations. In any case, the wide geographic sampling of these lines makes them valuable for the investigation of global demography, inference of past action of diverse forms of natural selection, analysis of gene family evolution, and relating these patterns of variation and their inferred historical causes to measured phenotypic differences among the lines, and they serve as a complement to the already valuable sets of reference lines of *D. melanogaster* drawn from other natural populations.

## 

## Supplementary Material

Supporting Information
