# Supplementary material for: Global Diversity Lines–A Five-Continent Reference Panel of Sequenced Drosophila melanogaster Strains
Source: G3 (Bethesda). 2015 Feb 11;5(4):593–603. doi: 10.1534/g3.114.015883 (PMC4390575; doi:10.1534/g3.114.015883)
Supplement: Supporting Information [file supp_5_4_593__index.html]

Global Diversity Lines–A Five-Continent Reference Panel of Sequenced Drosophila melanogaster Strains — Supporting Information 

# Global Diversity Lines–A Five-Continent Reference Panel of Sequenced *Drosophila melanogaster* Strains

## Supporting Information for Grenier *et al.*, 2015

**Files in this Data Supplement:**

- Supporting Information - Files S1-S2, Tables S1-S4, and Figures S1-S10 (PDF, 2 MB)
- File S1 - Expanded Materials and Methods (PDF, 229 KB)
- File S2 - Inversion breakpoint sequences. (PDF, 128 KB)
- Table S1 - Data Files (PDF, 127 KB)
- Table S2 - Read Counts by Line and by Chromosome. (PDF, 155 KB)
- Table S3 - Variant Calls by Line and Chromosome. (PDF, 172 KB)
- Table S4 - Genotypes of Known Large Inversions. (PDF, 195 KB)
- Figure S1 - Read Counts and Depth of Coverage by Chromosome. (PDF, 255 KB)
- Figure S2 - Summary of SNP Validation. (PDF, 165 KB)
- Figure S3 - SNP Validation Rate Correlates with Genotype Quality Score. (PDF, 551 KB)
- Figure S4 - Small Indel Validation Rate Correlates with Genotype Quality Score. (PDF, 330 KB)
- Figure S5 - SNP Genotype Counts Per Line. (PDF, 461 KB)
- Figure S6 - Small Indel Genotype Counts Per Line. (PDF, 502 KB)
- Figure S7 - Small Indel Length Distribution. (PDF, 245 KB)
- Figure S8 - Large Known Inversions and Residual Heterozygosity. (PDF, 309 KB)
- Figure S9 - Population-Specific Site Frequency Spectra for Four Classes of SNPs. (PDF, 646 KB)
- Figure S10 - Decay of Linkage Disequilibrium Over Distance. (PDF, 452 KB)
