# Supplementary material for: Global Diversity Lines–A Five-Continent Reference Panel of Sequenced Drosophila melanogaster Strains
Source: G3 (Bethesda). 2015 Feb 11;5(4):593–603. doi: 10.1534/g3.114.015883 (PMC4390575; doi:10.1534/g3.114.015883)
Supplement: Supporting Information [file supp_g3.114.015883_015883SI.pdf]

G3 · Genes | Genomes | Genetics

Global Diversity Lines - A five-continent reference panel of sequenced *Drosophila melanogaster* strains.

Jennifer K. Grenier*[,§,], J. Roman Arguello*[,§,1], Margarida Cardoso Moreira[§,1], Srikanth Gottipati[§,2],Jaaved Mohammed[†], Sean R. Hackett[§,3], Rachel Boughton[§], Anthony J. Greenberg[§,†], and Andrew G. Clark[§,†,4]

[§]Department of Molecular Biology and Genetics and [†]Department of Biological Statistics and Computational Biology, Cornell University, Ithaca, NY, [1]Center for Integrative Genomics, University of Lausanne, Lausanne, Switzerland, [2]Translational Medicine and Think Team, Otsuka Pharmaceutical Development and Commercialization, Inc., Princeton, NJ, [3]Quantitative and Computational Biology, Princeton University, Princeton, NJ.

* These authors contributed equally to this work.

[4]Corresponsing author: Department of Molecular Biology and Genetics, Cornell University, Ithaca, NY 14853. E-mail: ac347@cornell.edu

DOI: 10.1534/g3.114.015883

File S1

Expanded Materials and Methods

1. Global Diversity Lines

The Global Diversity Lines consist of 84+1 lines drawn from the initial set of 92 *D. melanogaster* lines inbred (sib-pair matings) for 12 generations from existing isofemale lines (Greenberg *et al.* 2010). The Global Diversity Lines were sampled from 5 populations: Beijing, China (15 lines; Begun and Aquadro 1995); Ithaca, NY USA (19 lines; Hill-Burns and Lazzaro 2004); Netherlands, Europe (18 lines; Bochdanovits and De Jong 2003); Tasmania, Australia (19 lines; Hoffman 2003); and Zimbabwe, Africa (14 lines; Ballard; Begun and Aquadro 1993). Seven of the original 92 lines were excluded from the final set because whole-genome sequencing indicated evidence of contamination (excessive heterozygosity or identity to another line). One additional line (ZW184) is included in the variant call set but excluded from all population-based analyses because it does not cluster with other Zimbabwe lines and may not have an African origin.

2. Genome Sequence Generation

Genomic DNA was extracted from ~50 non-virgin females per line with the Qiagen DNeasy Blood and Tissue kit. Each line was sequenced to a minimum 9x (average 12.5x) coverage on the Illumina HiSeq-2000 platform by BGI. In total, the whole-genome sequencing generated 1.7 B paired-end 100 nt reads (average 20.2 M per line). One line (ZW155) was independently sequenced to >100x depth from 3 additional WGS Illumina libraries made from the same genomic DNA stock by BGI.

3. Genome Alignments of Short Read Data

For SNP and small indel variant detection, raw sequence reads were mapped to the *D. melanogaster* reference (5.34) using BWA aln and BWA sampe (v0.5.9; (Li and Durbin 2009) with default parameters. Paired-end reads were reannotated with Samtools fixmate (v0.1.19; (Li *et al.* 2009) prior to merging bam files for all lines. To address alignment consistency between lines near small indels, the merged bam file was locally realigned using GATK v1.2 RealignerTargetCreator and IndelRealigner (McKenna *et al.* 2010; DePristo *et al.* 2011). The frequency of PCR duplicates in the paired-end sequences was so low (<<1%) that duplicate reads did not need to be removed.

Inversions were detected as part of a larger effort to identify structural variation in the Global Diversity Lines (M. Cardoso-Moreira, J. R. Arguello, D. Riccardi, S. Gotipatti, J. K. Grenier and A. G. Clark, unpublished). For this effort the raw sequence reads were mapped to the *D. melanogaster* reference (r5.34) using two different aligners, Novoalign (v2.07.11;

www.novocraft.com) and Mosaik (v1.1.0021; Lee *et al.* 2014). Default parameters were used with Novoalign with one exception: the penalty for gap extension was lowered (i.e. -x 6). Mosaik was run using Mosaik Jump (-hs 15), Mosaik Aligner (-hs 15 -mm 15 -mhp 100 -act 35 -bw 35) and Mosaik Sort (-rmm). See the section "Identification of Inversions" below for a description of the pipeline for calling inversion breakpoints.

4. SNP and Small Indel Calls

SNP and small indel calling was a multi-step process using GATK (v1.2; McKenna *et al.* 2010; DePristo *et al.* 2011) best practices. First, a preliminary run of the GATK UnifiedGenotyper generated 'preliminary' SNP calls that were subsequently processed and used as a 'truth set' for GATK Base Quality Score Recalibration (BQSR). Second, the GATK UnifiedGenotyper was run again on the BSQR merged bam file to call both SNPs and small indels (below). The resulting SNP VCF file was then used for GATK Variant Quality Score Recalibration (VQSR), using the filtered SNPs from the preliminary run as the training set.

4a. Base Recalibration

The 'truth set' used for the Base Recalibration was constructed with a set of high confidence SNP calls generated from the first pass GATK run across the full initial set of 92 lines. We designated SNP calls as 'high confidence' if they met the following criteria: 1) site quality score ≥ 30, 2) cumulative read depth greater than 30 and less than 190, and 3) If heterozygous in all variant lines, variant read frequency consistent with binomial expectation ($p \leq 0.05$). In addition, we examined the quality of the SNPs with respect to genomic annotations and the transition/transversion ratio (generated with SNPeff; Cingolani *et al.*)). These filters resulted in over 1 million 'high quality' SNPs with which to examine SNP-call covariates, and thus to recalibrate GATK's SNP calling model. The recalibrated model was then applied to the complete data set. We carried out the same procedure independently to identify 'high confidence' SNPs from the deeply sequenced (100x) ZW155 line. Although the total SNP set was smaller than for the 92 lines' set, the results in Base recalibration were minimal.

Examples of the GATK commands used for the Base Recalibration steps (using chr3L):

1) Run CountCovariates with standard covariats for each chromosome

```
java -Xmx16g -jar GenomeAnalysisTK.jar -nt 8 -R Dmel_r5.34.fasta -knownSites
Filtered_Truth_SNPs_3L.vcf -I Dmel_3L.realigned.bam -recalFile Dmel_filtered_cov_3L.csv -
T CountCovariates --standard_covs
```

2) Run TableRecalibration for each chromosome:

```
java -Xmx16g -jar GenomeAnalysisTK.jar -R Dmel_r5.34.fasta -I Dmel_3L.realigned.bam -T
TableRecalibration -recalFile Dmel_filtered_cov_3L.csv -o Dmel_filtered_recal_3L.bam
```

3) Re-genotyped with GATK's Unified Genotyper:

This was done with the same parameters as the initial 'first pass' genotyping but, due to memory requirements, were processed in chromosome segments and later concatenated:

```
java -Xmx16g -jar GenomeAnalysisTK.jar -R Dmel_r5.34.fasta -T UnifiedGenotyper -I
Dmel_filtered_recal_3L.bam -L 3L:20000001-23011544 -o Dmel_filtered_recal_3L.bam_5.vcf -
glm BOTH -stand_emit_conf 4 -stand_call_conf 10 -A DepthOfCoverage --output_mode
EMIT_ALL_SITES -dcov 100 -nt 8
```

4b. Variant Recalibration

SNP calls generated by GATK's Unified Genotyper were further refined using the variant quality score recalibration (VQSR). It assigns a well-calibrated probability to each variant call in a raw call set based on a truth set and uses this score to filter the raw calls. UG generated SNP calls which have more than 2 homozygous variant calls, a Phred-score greater than 20, and a mapping quality greater than 20 were used as a truth set.

1) Select all SNP calls from UG

```
java -Xmx4g -jar GenomeAnalysisTK.jar -R dmel-all-r5.34.fasta -T SelectVariants --variant
$VCF -o $VCF.snp_ALL.vcf -selectType SNP -restrictAllelesTo ALL
```

2) Generate a truth set

```
java -Xmx4g -jar GenomeAnalysisTK.jar -R dmel-all-chromosome-r5.34.fasta -T
SelectVariants --variant ${VCF}.snp_ALL.vcf -o ${VCF}.snp_ALL.filtered.vcf -select
"vc.getHomVarCount() > 2 && QUAL > 20.0 && MQ > 20.0"
```

3) Use VQSR to compute variant scores

```
java -Xmx4g -jar GenomeAnalysisTK.jar -T VariantRecalibrator -R dmel-all-chromosome-
r5.34.fasta -input $VCF.snp_ALL.vcf --qualThreshold 20.0 --percentBadVariants 0.03 --
maxGaussians 10 --mode SNP -
resource:GATK_FILTERED_${CHROM}_SNPS_ALL,known=false,training=true,truth=true,prior=10.0
${VCF}.snp_ALL.filtered.vcf -an QD -an HaplotypeScore -an MQRankSum -an ReadPosRankSum -
an FS -an MQ -an DP -an InbreedingCoeff -recalFile $VCF.variantRecal_after_baseRecal -
tranchesFile $VCF.tranches -rscriptFile $VCF.plots.R
```

4) Re-annotation of variant sites based on VQSR scores

```
java -Xmx4g -jar GenomeAnalysisTK.jar -T ApplyRecalibration -R dmel-all-chromosome-
r5.34.fasta -input $VCF.snp_ALL.vcf --ts_filter_level 99.0 -tranchesFile $VCF.tranches -
recalFile $VCF.variantRecal_after_baseRecal -o $VCF.snp_ALL.vcf.variantRecal.vcf
```

Sites with VQSLOD>99.9 were annotated as 'VQSLOD-verylow' and sites with VQSLOD>99 were annotated as 'VQSLOD-low'.

5. Defining Heterozygous Blocks

To investigate the 'blockiness' of heterozygous runs, the VCF file's genotypes at each variant site were converted into a binary sequence for each individual fly line (0 = homozygous, 1 = heterozygous). Within windows sliding along chromosomes, the observed number of consecutive heterozygous sites was compared with the expected number, where the state of each variant site was assumed to be independent outcomes of a multinomial distribution (the observed to expected ratio):

$$B = (Het^C / W) / ((Het^T / W) \times (Hom^T / W))$$

where *Het*^{C} is the number of consecutive heterozygous sites within the window of size *W*, *Het*^{T} is the total number of heterozygous sites within the window, and *Hom*^{T} is the total number of homozygous sites.

Two window sizes (20 SNPs or 2,000 SNPs) were tested to determine whether window size affected the ability to localize the ends of heterozygous blocks. The overlap between blocks of enriched heterozygosity was easily observable for the two window sizes, with the 20 SNP window producing more isolated 'spikes', as expected. Because there was good agreement between the two window sizes, we opted to use the 20 SNP window. Empirically, it was clear that windows having *B* values greater than 0.25 marked outlier regions. These small regions were then iteratively merged to form the larger heterozygous blocks. Adjacent regions were merged into a single block when separated by no more than 350 kb; the minimum size to retain a heterozygous block (after merging) was 200 kb. These regions were summarized in BED format and used in downstream analyses.

6. Variant Call Validation and Filtering

SNP calls were validated using two strategies. First, one line (ZW155) was independently sequenced to 100x depth by BGI (using 3 additional Illumina libraries distinct from the 10x coverage library). SNPs were called in this dataset by aligning paired-end 100 nt reads to the *D. melanogaster* reference genome (v5.34) with BWA (v0.5.9; (Li and Durbin 2009) using default parameters and then generating a base-count file at each variant position with coverage > 100 (Galaxy samtools_mpileup v0.0.1); homozygous genotypes were called at sites with >90% of reads having the same base, and homozygous genotypes were called at sites with 2 base identities each represented in >10% of reads.

Second, ddRAD libraries (Peterson *et al.* 2012) were generated for a subset of 12 lines (4 lines from Zimbabwe and 2 from each other source population). For each line, genomic DNA was digested with *EcoR*I and *Taq*$^{\alpha}$I and ligated to barcoded Illumina adaptors. The ddRAD libraries were separately size-selected on an agarose gel and amplified with TruSeq-compatible primers before pooling for Illumina sequencing (100 nt, single end reads) on a Hi-Seq2000. The ddRAD reads were processed to require and trim off the sample-specific barcode and an anchoring *EcoR*I site (AATTC) before mapping to the *D. melanogaster* reference genome (v5.34) with BWA aln and samse (v0.5.9; (Li and Durbin 2009) using default parameters. Similar to the 10x whole-genome coverage dataset, bam files were merged and realigned in indel intervals using GATK RealignerTargetCreator and IndelRealigner. Similar to the ZW155 100x validation dataset, SNP genotypes for each line were determined by generating a base-count file at each variant position with coverage > 100 using pileup (Galaxy); homozygous genotypes were called at sites with >90% of reads having the same base and homozygous genotypes were called at sites with 2 bases each represented in >10% of reads.

SNP calls for all lines in the original 10x dataset were filtered based on minimum validation rates for each genotype category and site type (PASS, VQSLOD-low, VQSLOD-verylow). The GQ at which each genotype category and site type exceeded 90% was used as a minimum cutoff for inclusion in the final SNP dataset:

Site Type	REF	ALT	HET (in block)
PASS	NA	NA	30
VQSLOD-low	NA	30	30
VQSLOD-verylow	7	30	99

In addition to SNP calls with GQ below the cutoff for each genotype category and site type above, all heterozygous SNP calls outside of heterozygous blocks were removed from the final SNP dataset. Finally, SNP calls within 5 nt of a small indel call (GQ>25) in the same line were also removed from the final dataset. Note that the GQ cutoff for small indel calls used here is more conservative than the final small indel call set (below) to minimize false-positive SNP calls adjacent to small indels.

Small indel calls were validated with the ZW155 100x dataset described above, using a similar strategy. Small indels in the 100x dataset were called independently with GATK UnifiedGenotyper (v1.2) and indel identity and genotype were compared to the ZW155 10x calls. A minimum validation rate of 75% was used to establish a minimum GQ for each genotype category, based on the validation rate of heterozygous indel calls with GQ≥7 for homozygous REF calls at indel sites, GQ≥30 for homozygous ALT indels and GQ=99 for heterozygous indels within heterozygous blocks. Small indel calls with GQ below these cutoffs were removed from the final dataset, as well as all heterozygous indel calls outside heterozygous blocks.

7. SNP Annotations Using Genomic Features

SNPs were annotated with respect to FlyBase genomic features using the SNPeff pipeline (v2.0.3; Cingolani *et al.*). VCF files generated from the above steps were provided to SNPeff, along with the reference genome (dm5.34) and FlyBase annotations using the following commands for each chromosome:

```
java -jar snpEff.jar eff -i vcf dm5.34 INPUT_2R.vcf -s 2R_SNPeff.html >
2R_SNPeff_summary.out
```

8. Identifying Regions of IBD

Germline (Gusev *et al.* 2009) was used to identify putative regions of genetic identity by decent (IBD) between all pairs of the 92 lines (non-defaulting settings: -min_m 1, -err_hom 1, -w_extend). Germline does not allow for missing data, so the 'full data set' of 92 lines was reduced to the subset of sites with all 184 alleles genotyped. This necessarily reduced the genetic variation in the samples, and thus represents conservative (overestimated) segments of putative IBD.

Our goals with the IBD analyses were twofold. First, we aimed to separate segments of candidate IBD that are more likely to have arisen from sampling closely related individuals from those segments of high identity that either have arisen by chance or as a result of overall low diversity. Second, measures of putative IBD are also useful for providing additional information regarding particular pairs of lines that stand out as problematic due to either unexpected shared identity (label switching) or unexpected amounts of putative IBD (contamination).

To identify the segments of IBD that would be retained within our masking files, we first ignored regions that fell within the lowly recombining regions of the pericentromeric and telomeric regions, as well as the fourth chromosome (Langley *et al.* 2012). We based the limits of the low recombination regions using the "*Drosophila melanogaster* Recombination Rate Calculator Version 2.3" (Fiston-Lavier *et al.* 2010), leaving the following chromosome segments for analyses:

Chrom	Region
X	2,222,391 – 20,054,556
2L	464,654 – 15,063,839
2R	9,551,429 – 20,635,011
3L	1,979,673 – 12,286,842
3R	12,949,344 – 25,978,664

The lowly recombining regions excluded from the above segments contained the greatest density of putative IBD segments, with many recurring both within and between populations. If IBD segments overlapped the low recombination boundaries, only segments having greater than 75% of their total length outside the low recombining regions were considered further.

Between- and within-population IBD segments that were identified by Germline (IBD^B *and* IBD^W, respectively) were separated, and the largest stretch of IBD^B was taken as an estimate for the extent of IBD observable by chance (3.8 Mb); IBD^W segments less than 3.8 Mb were not considered further. In total, 30 segments shared between individuals within the same populations were identified. Segments that retained IBD were masked in one randomly selected line for population genetic analyses.

9. Genome Callability

The following criteria were used to identify regions of poor mapping quality: a depth of coverage between 510 and 2040 inclusive (between one half and twice the average per-site depth including all lines) and that no more than 20% of covering reads have mapping quality zero. We found that about 88% of the reference genome was callable. Uncallable intervals are recorded the a bed file 'whole_genome_masked_intervals.bed'.

10. Identification of Inversions

We developed a pipeline aimed at identifying the breakpoints of inversions segregating in our dataset that consisted of two main steps. The first was a discovery step where bioinformatic tools (described below) were used to generate an initial set of candidate inversions. The second step consisted of an empirical evaluation of the initial set of candidate inversions by PCR and generation of breakpoint sequences using Sanger sequencing.

We created the initial set of candidate inversions by running two independent pipelines designed to detect inversions: Delly (i.e. invy; Rausch *et al.* 2012) and an in-house pipeline designed around BLAT (Kent 2002). The two pipelines identify inversions using complementary approaches: Delly identifies inversion calls based on paired-end information whereas the Blat-based pipeline identifies inversions based on split-read information. We ran Delly's invy module (v0.0.9) on each line using the alignments created by Novoalign (v2.07.11). Delly was run with default parameters, but we limited the detection of inversions to reads with a mapping quality score ≥ 20. The in-house pipeline based on BLAT is a simple extension of the pipeline previously developed by our group (Cardoso-Moreira *et al.* 2012). Briefly, the pipeline starts with the set of reads that Mosaik (v1.1.0021) failed to align. Those unaligned reads were re-aligned using BLAT (v3.4, -oneOff=1). Inversions were detected by selecting those reads that show a 'split signature', i.e. one read leads to two non-overlapping alignments, each on a different strand. We further required that the breakpoints detected using this split-read signature were located ≥ 30 bp away from the limits of the reads. To all calls made by Delly and the BLAT-based pipeline we applied the following filters: 1) for each genome inversion breakpoints had to be supported by at least 3 reads; 2) the inversion breakpoints could not overlap known transposable elements (annotated in Flybase; St Pierre *et al.* 2014) or identified by running RepeatMasker (Smit *et al.* 1996) on the 100 bp flanking each of the putative breakpoints); and 3) the candidate inversions had to be ≥ 1 Mb in size. In total, *each* pipeline identified a unique set of 109 candidate inversions with only 12 overlapping between the two sets. The lack of overlap was expected because the two pipelines use different signatures in the sequence data to identify inversions. Critically, all 8 *D. melanogaster* inversions with known breakpoints were independently identified by our approach.

From the set of 218 inversions predicted by the two pipelines, we designed primers to confirm both of the inversion breakpoints of a subset of 43. This subset includes 7 inversions with already mapped breakpoints and is heavily biased toward inversions predicted in multiple lines (as opposed to being private to one genome). Out of the 43 inversions tested we only obtained clear PCR bands for 17 (40%). However, it should be noted that we also only obtained clear PCR bands for 3 of the 7 inversions tested that already have mapped breakpoints. These results suggest that amplifying inversion breakpoints can be challenging, and that our approach is likely to have a high false negative rate in addition to a high false positive rate. In order to confirm the specificity of the PCR amplifications we attempted to sequence using Sanger sequencing 15 of the 17 breakpoints. We successfully sequenced 12, but of these only 6 proved to be true inversions. The remaining sequences suggested mis-

priming during PCR or the presence of structural variation at the breakpoints but not of inversions. After these efforts we generated breakpoint sequences for 5 of the 8 inversions with already known breakpoints and for two inversions with previously unknown molecular breakpoints (File S1). One of the inversions matches well the cytogenetic limits for In(3L)62D:68A described by Lemeunier and Aulard 1992 as a recurrent endemic. The other inversion with previously unknown molecular breakpoints, In(3R)13-72, does not match perfectly the cytogenetic limits of any inversion described by Lemeunier and Aulard 1992 but is located in the proximity to several inversions described. It should be noted that all 10 inversions with molecularly mapped breakpoints possess relatively simple breakpoint structures (Corbett-Detig *et al.* 2012). They were identified by our pipeline which, by design, attempts to exclude regions associated with transposable elements and other types of repeats, which are often associated with the genesis of inversions (Ranz *et al.* 2007). This suggests a potential significant ascertainment bias associated with the known inversion breakpoints.

11. Genotyping of Inversions

We inspected the genome sequences of the Global Diversity Lines for all 10 inversions with known molecular breakpoints. We used the breakpoint sequences that we generated (File S2) as part of our effort to identify inversions and those made available by Corbett-Detig and colleagues (Corbett-Detig *et al.* 2012). For In(3R)P we used the sequences deposited in Genbank by Matzkin and colleagues (Matzkin *et al.* 2005). Table 1 describes the origin of the breakpoint data used to genotype each of the 10 inversions. We genotyped these inversions *in silico* by mapping the raw genomic sequence reads of each genome against the reference genome sequence and all inversion breakpoints using BWA(Li and Durbin 2009). Inversions were called as being present in a given genome when reads from that genome spanned the inversion sequence breakpoints. We required that at least 2 reads spanned the inversion breakpoint with the latter not located within the last 15 bp of the reads. Inversions were called as homozygous when there were reads matching the inversion breakpoint but not the equivalent region in the reference genome. Inversions were called as heterozygous when there were reads matching both the inversion breakpoint and the equivalent region in the reference genome.

We genotyped individual flies for inversion breakpoints and for the reference chromosomal arrangement using a combination of novel primer designs and previously reported PCR assays for In(2L)t (Andolfatto *et al.* 1999) and In(2R)NS, In(3L)P, In(3R)K, and In(3R)Mo (all from Corbett-Detig *et al.* 2012). We also developed SNP genotyping assays for variant sites segregating within a line carrying an inversion that fall within a restriction site (the REF allele contains the restriction site and the PCR product is cut into two smaller bands).

Inversion	Variant	Primer 1	Primer 2	PCR Product Size ALT	REF
In(2L)t	Inversion breakpoint	GACTCTTTCTGCTTCGATCACTAAG	TATTTTGGTGGCCTGTTTCAG	250	None
	No inversion	TATTTTGGTGGCCTGTTTCAG	AAACACCACCAACGACATCC	none	240
	2L:2420388 T/C SNP	ACTAATCAGAGGCGCTTACATC	CTTGCTGCTATGTCACGCAC	298	HindIII: 184+114
In(2R)NS	Inversion breakpoint	TGGCCTGCTTCTGGTCCTCT	GGCGAGCCATCATTGTTATC	249	None
	No inversion	TGGCCTGCTTCTGGTCCTCT	AGAAGCACGCTGAGGAAATG	none	338
	2R:13048623 C/A SNP	CTGTGATACCCTACGCCGAC	AGCAAGTACGAGTGGAGAAGAC	218	HindIII: 140+77
In(3L) 62D:68A	Inversion breakpoint 1	AGAAGCTCTTTCGCAAATGG	GCATCGCAAGATTGTTTCC	800	None
	Inversion breakpoint 2	GCTGCAATTGTACATCGTTCC	TCACTTGGATTGCTTTGCTTG	750	None
	No inversion	GCATCGCAAGATTGTTTCC	TCACTTGGATTGCTTTGCTTG	none	500
	3L:1,325,296 A/G SNP	CATGGCCAGGTAGAAGAAGC	TGCAATGGATTTGTGACTGG	159	XbaI: 39+120
	3L:5,798,786 A/T SNP	CACGGGTATTCCACTCAAGG	GGATTCACCTGATGATACAACG	292	XbaI: 93+199
In(3L)P	Inversion breakpoint	CGGGAATGGTAGCTAGACCA	GTGAGCTCAACCCATTCGGT	306	None
	No inversion	GCTGATTCGCTTTGTCTTCG	GCCTGAAGTGCTGAAAGTGG	none	272
	3L:9034742 G/C SNP	AATGGATATGCGGATGCAG	TTCGATCAACACCCATAGACC	110	XbaI: 78+32
In(3R)K	Inversion breakpoint	TCTGACCCACTCTCCACTTG	CGAAAACCACAAGTACGCCTT	244	None
	Reference	GGGCATACACGAAAGAAGGTC	AGCCCGTGTGGTAATCGTAG	none	236
	3R:21878478 G/A SNP	TGATTAGGCGTTGAAGCCCTG	AGGGTGTGCGCGATTCTAAG	299	HindIII: 224+74
In(3R)Mo	Inversion breakpoint 1	TTGAAAGGTGATCCCAGATATAAG	TCGCCACAGTGTATGACTGC	278	None
	Inversion breakpoint 2	ACCTCACTGCGGATGAAGAG	TCCATGGCAATACCTTCACA	400	None
	No inversion	TCGCCACAGTGTATGACTGC	TCCATGGCAATACCTTCACA	none	308
	3R:17827239 G/T SNP	TTGCAGCAACAACAAATGCG	TGTTCTTGCCGTGTGCTG	250	BamHI: 161+89

Single-fly genomic DNA was isolated for PCR genotypig according to Gloor *et al* (Gloor *et al.* 1993), using LongLife Proteinase K (GBiosciences). PCR reactions consisted of 2 µL single-fly gDNA, 500 nM each PCR primer, 20 mM Tris pH 8, 50 mM KCl, 1.5mM $MgCl_2$, 0.2mM dNTPs, 5% DMSO, and 0.5ul *Taq* polymerase in 50 µL total volume, with cycling conditions 95 °C/15 min; 95 °C/15 sec – 53 °C/10 sec -72 °C/90 sec] x 40 cycles; 72 °C/5 min. For SNP assays, the PCR product was digested by adding the appropriate restriction enzyme in 1x PCR buffer and incubating at 37 °C for 3 hours; fragment sizes were resolved on 1.5% Agarose gels (1x TAE).

12. Genetic Tests of Chromosome Homozygosity

Individual males were selected from fly stocks and crossed to virgins from a double-balancer strain (Bloomington stock #2475: *w*[*]; T(2;3)*ap*[*Xa*], *ap*[*Xa*]/*CyO*; *TM3*, *Sb*[1]). Multiple sib-pair F1 crosses (*CyO/*+; *TM3/*+) were set up from each parental cross to sample each possible F1 x F1 genotype combination. F1 adults were individually genotyped (see above) after the F2 generation was initiated to determine the identity of the chromosome inherited from the founder male. F2 progeny were scored for dominant markers on both balancer chromosomes (minimum 20 F2 progeny required per cross).

13. Diversity Estimates

Diversity estimates and summary statistics were computed using the VariScan package (v2.0.3; (Vilella *et al.* 2005; Hutter *et al.* 2006). For input to VariScan, VCF files were first filtered using the IBD and genome callability masking files and

then converted to HapMap format. Conversion was accomplished by using VCFtools (v0.1.11; (Danecek *et al.* 2011) to convert the VCF files to TPED format, which was then converted to HapMap format using the Perl script "convert_tped_to_hapamp.pl" available on GitHub (https://gist.github.com/pamag/2069211).

To generate the data for Figure 8A, summaries were calculated for windows sized by the number of polymorphic sites (WindowType = 2), with the size equal to 10,000 for autosomes and chrX, and 500 for chr4 (WidthSW = 10000; WidthSW = 500). The stride size was 5,000 sites for autosomes and chrX, and 250 for chr4 (JumpSW = 5000; JumpSW = 250). Additionally, summary statistics were computed using the total number of mutations (UseMuts = 1), and the minimum number sequences at each site were required to be at least 14 or 15 (NumNuc = 14; NumNuc = 15).

14. F_{ST} and Migration:

F_{ST} between each pair of populations was calculated using the unbiased approach of Weir and Clark (1984), which allows for unequal sampling between populations. To generate Figure 4, *m*, the per generation migration rate, was approximated by using the equilibrium from the Wright Island model , $F_{ST} = 1 / (4N_e m + 1)$, and solving for *m*, using 10^6 as an estimate of the effective population size, N_e.

15. Identifying Small Intronic and 4-fold Degenerate SNPs

For several population genetic analyses (i.e examining population structure or demographic effects), obtaining estimates from sequences that behave the most neutrally (most free of selective pressures) can be informative. In the *D. melanogaster* genomes the two classes of sites that have been shown to behave most neutrally are SNP within short (<=65 bp, bases 8-30) introns (SI) and 4-fold degenerate sites (4D) (Parsch *et al.* 2010). To extract SI sites that are polymorphic within the dataset, all SNPs that were annotated as intronic based on the SNPeff output (above) were outputted in "bed" format file. A second bed file that was composed of SI intronic start/end coordinates based on the genic annotation of reference *D. melanogaster* genome (r5) was generated. Introns that were not 'dedicated' (found to be coding in >=1 isoform) were excluded. These two bed files were intersected using BEDtools (v2.17.0;(Quinlan and Hall 2010), generating a list of SNPs in our dataset that fall within SI. Similarly, a bed file was generated for all 4-fold degenerate positions (as annotated by SNPeff) that are polymorphic within the dataset. Redundant positions that resulted from isoforms were removed.

16. Whole Genome Alignment and Species Divergences

For accurate computation of *k*, a measure of divergence per site, and the Polymorphism / Divergence ratio, we created a custom-built multiple genome alignment using a recent, revised *D. simulans* genome assembly superior to the earlier mosaic assembly of this species (Hu *et al.* 2013). We created a whole-genome alignment of the 5 *melanogaster*-subgroup species using publically accessible genome assemblies for *D. melanogaster* (dm3), *D. simulans* (droSim2), *D. sechellia* (droSec1), *D. erecta* (droEre2) and *D. yakuba* (droYak2). Besides the revised *D. simulans* genome assembly, all other genome assemblies were downloaded from the UCSC genome browser (http://genome.ucsc.edu/). Pairwise alignments of *D. melanogaster* to the other assemblies were created using LASTZ (v1.03.34; Harris 2007). These pairwise alignments were further refined into single-coverage alignments using the chaining and netting utilities as prescribed from the UCSC genome browser. Subsequently, these were later merged into a single whole-genome multiple alignment utilizing the *roast* program of the MULTIZ software package ; (Blanchette *et al.* 2004; updated Jan 21, 2009). This 5-way multiple-alignment file (MAF) is available upon request and the alignment is viewable at http://genome-mirror.cshl.edu/. To access this alignment, users should navigate to the *D. melanogaster* genome assembly (dm3) and enable the "Conservation 5way" track.

We computed divergence per site (*k*), the average number of nucleotide substitutions per site between the *D. melanogaster* and *D. simulans,* by first counting the number of nucleotide differences between the two species conditioned on the multiple alignment. Estimates of *k* were computed using the same window sizes as the measure of nucleotide diversity. In each window, sites without an aligned base in the *D. simulans* assembly were filtered. The ratio of divergence per window length (*D*) were then corrected for multiple substitutions using the Jukes-Cantor correction (Jukes and Cantor 1969), $k=-(3/4)\ln(1 - (4/3)D)$.

References

Andolfatto, P., J. D. Wall, and M. Kreitman, 1999 Unusual haplotype structure at the proximal breakpoint of In(2L)t in a natural population of Drosophila melanogaster. Genetics 153: 1297–311.

Arnold, B., R. B. Corbett-Detig, D. Hartl, and K. Bomblies, 2013 RADseq underestimates diversity and introduces genealogical biases due to nonrandom haplotype sampling. Mol. Ecol. 22: 3179–90.

Ballard, W. Isofemale D. melanogaster lines ('ZH') collected near Victoria Falls, Tasmania.

Begun, D. J., and C. F. Aquadro, 1993 African and North American populations of Drosophila melanogaster are very different at the DNA level. Nature 365: 548–50.

Begun, D. J., and C. F. Aquadro, 1995 Molecular Variation at the vermilion Locus in Geographically Diverse Populations of Drosophila melanogaster and D. simulans. Genetics 140: 1019–1032.

Blanchette, M., W. J. Kent, C. Riemer, L. Elnitski, A. F. A. Smit *et al.*, 2004 Aligning multiple genomic sequences with the threaded blockset aligner. Genome Res. 14: 708–15.

Bochdanovits, Z., and G. De Jong, 2003 Temperature dependent larval resource allocation shaping adult body size in Drosophila melanogaster. J. Evol. Biol. 16: 1159–67.

Cardoso-Moreira, M., J. R. Arguello, and A. G. Clark, 2012 Mutation spectrum of Drosophila CNVs revealed by breakpoint sequencing. Genome Biol. 13: R119.

Cingolani, P., A. Platts, L. L. Wang, M. Coon, T. Nguyen *et al.*, 2012 A program for annotating and predicting the effects of single nucleotide polymorphisms, SnpEff: SNPs in the genome of Drosophila melanogaster strain w1118; iso-2; iso-3. Fly (Austin). 6: 80–92.

Corbett-Detig, R. B., C. Cardeno, and C. H. Langley, 2012 Sequence-based detection and breakpoint assembly of polymorphic inversions. Genetics 192: 131–7.

Danecek, P., A. Auton, G. Abecasis, C. A. Albers, E. Banks *et al.*, 2011 The variant call format and VCFtools. Bioinformatics 27: 2156–8.

Davey, J. W., T. Cezard, P. Fuentes-Utrilla, C. Eland, K. Gharbi *et al.*, 2013 Special features of RAD Sequencing data: implications for genotyping. Mol. Ecol. 22: 3151–64.

DePristo, M. A., E. Banks, R. Poplin, K. V Garimella, J. R. Maguire *et al.*, 2011 A framework for variation discovery and genotyping using next-generation DNA sequencing data. Nat. Genet. 43: 491–8.

Fiston-Lavier, A.-S., N. D. Singh, M. Lipatov, and D. A. Petrov, 2010 Drosophila melanogaster recombination rate calculator. Gene 463: 18–20.

Gautier, M., J. Foucaud, K. Gharbi, T. Cézard, M. Galan *et al.*, 2013 Estimation of population allele frequencies from next-generation sequencing data: pool-versus individual-based genotyping. Mol. Ecol. 22: 3766–79.

Gloor, G. B., C. R. Preston, D. M. Johnson-Schlitz, N. A. Nassif, R. W. Phillis *et al.*, 1993 Type I repressors of P element mobility. Genetics 135: 81–95.

Greenberg, A. J., S. R. Hackett, L. G. Harshman, and A. G. Clark, 2010 A hierarchical Bayesian model for a novel sparse partial diallel crossing design. Genetics 185: 361–73.

Gusev, A., J. K. Lowe, M. Stoffel, M. J. Daly, D. Altshuler *et al.*, 2009 Whole population, genome-wide mapping of hidden relatedness. Genome Res. 19: 318–26.

Harris, R. S., 2007 Improved pairwise alignment of genomic DNA: Pennsylvania State University.

Hill-Burns, E., and B. Lazzaro, 2004 Isofemale D. melanogaster lines collected in Ithaca, NY.

Hoffman, A., 2003 Isofemale D. melanogaster lines from Tasmania, Australia.

Hu, T. T., M. B. Eisen, K. R. Thornton, and P. Andolfatto, 2013 A second-generation assembly of the Drosophila simulans genome provides new insights into patterns of lineage-specific divergence. Genome Res. 23: 89–98.

Hutter, S., A. J. Vilella, and J. Rozas, 2006 Genome-wide DNA polymorphism analyses using VariScan. BMC Bioinformatics 7: 409.

Jukes, T., and C. Cantor, 1969 Evolution of protein molecules, pp. 21–132 in *Mammalian Protein Metabolism*, edited by H. Munro.

Kent, W. J., 2002 BLAT--the BLAST-like alignment tool. Genome Res. 12: 656–64.

Langley, C. H., K. Stevens, C. Cardeno, Y. C. G. Lee, D. R. Schrider *et al.*, 2012 Genomic variation in natural populations of Drosophila melanogaster. Genetics 192: 533–98.

Lee, W.-P., M. P. Stromberg, A. Ward, C. Stewart, E. P. Garrison *et al.*, 2014 MOSAIK: a hash-based algorithm for accurate next-generation sequencing short-read mapping. PLoS One 9: e90581.

Lemeunier, F., and S. Aulard, 1992 Inversion Polymorphism in Drosophila melanogaster, pp. 339–406 in *Drosophila Inversion Polymorphism*, edited by C. B. Krimbas and J. R. Powell. CRC Press.

Li, H., and R. Durbin, 2009 Fast and accurate short read alignment with Burrows-Wheeler transform. Bioinformatics 25: 1754–60.

Li, H., B. Handsaker, A. Wysoker, T. Fennell, J. Ruan *et al.*, 2009 The Sequence Alignment/Map format and SAMtools. Bioinformatics 25: 2078–9.

Matzkin, L. M., T. J. S. Merritt, C.-T. Zhu, and W. F. Eanes, 2005 The structure and population genetics of the breakpoints associated with the cosmopolitan chromosomal inversion In(3R)Payne in Drosophila melanogaster. Genetics 170: 1143–52.

McKenna, A., M. Hanna, E. Banks, A. Sivachenko, K. Cibulskis *et al.*, 2010 The Genome Analysis Toolkit: a MapReduce framework for analyzing next-generation DNA sequencing data. Genome Res. 20: 1297–303.

Parsch, J., S. Novozhilov, S. S. Saminadin-Peter, K. M. Wong, and P. Andolfatto, 2010 On the utility of short intron sequences as a reference for the detection of positive and negative selection in Drosophila. Mol. Biol. Evol. 27: 1226–34.

Peterson, B. K., J. N. Weber, E. H. Kay, H. S. Fisher, and H. E. Hoekstra, 2012 Double digest RADseq: an inexpensive method for de novo SNP discovery and genotyping in model and non-model species. PLoS One 7: e37135.

Quinlan, A. R., and I. M. Hall, 2010 BEDTools: a flexible suite of utilities for comparing genomic features. Bioinformatics 26: 841–2.

Ranz, J. M., D. Maurin, Y. S. Chan, M. von Grotthuss, L. W. Hillier *et al.*, 2007 Principles of genome evolution in the Drosophila melanogaster species group. PLoS Biol. 5: e152.

Rausch, T., T. Zichner, A. Schlattl, A. M. Stütz, V. Benes *et al.*, 2012 DELLY: structural variant discovery by integrated paired-end and split-read analysis. Bioinformatics 28: i333–i339.

Sezgin, E., D. D. Duvernell, L. M. Matzkin, Y. Duan, C.-T. Zhu *et al.*, 2004 Single-locus latitudinal clines and their relationship to temperate adaptation in metabolic genes and derived alleles in Drosophila melanogaster. Genetics 168: 923–31.

Smit, A., R. Hubley, and P. Green, 1996 RepeatMasker Open-3.0.

St Pierre, S. E., L. Ponting, R. Stefancsik, and P. McQuilton, 2014 FlyBase 102--advanced approaches to interrogating FlyBase. Nucleic Acids Res. 42: D780–8.

Vilella, A. J., A. Blanco-Garcia, S. Hutter, and J. Rozas, 2005 VariScan: Analysis of evolutionary patterns from large-scale DNA sequence polymorphism data. Bioinformatics 21: 2791–3.

Weir, B., and C. C. Clark, 1984 Estimating F-Statistics for the Analysis of Population Structure. Evolution (N. Y). 38: 1358–1370.

File S2

Inversion breakpoint sequences

>In(3R)13-72_proximal
GTCCTCGCTCTCTGTCTTGGTTTCTGTCTTGGCATTCTTGCTGGACGCCCCTCCGCCATTCTCCTCCAGC
CACTTTTCAAAGACTCCAATGGCCGCATTGATGTTCTTCAGCTTCTCCTCCGCCTTGGTCACTAGAATGG
TAGGTTTAGTGCTGGATTCAACGCATCCCTTGGGCACAAGCACTCACTTGTCAGGACTCTTTTGGCCCGG
CCAAGCAAACCGCGCACCGTCAGCTTGCGATACTGCATGTCGTGACTCTCCAGCAGCTTCAACGTCTCTA
ACGCCTTCTCCATGATGACACCACGGAGATGACCTTCAGCTTGAACAGTTTGGATCTGGATAATATTCGT
CCGCCGTCAGGAATGGAATCGCTGAACAGCTGCTACCAGGATCACTCCCAGCCTAGTTCTCTGCGCCAGC
CGATGCCCAGCAAGAGTCCACGCTTCGCGCGAAAAATGTTTCCAGCCAATTTGGTAGCCAGACGAGCACT
GGGTCACTTAGCTGGCAGTGCGGAGAGCGTGAACTCCAGCTGCAACCTGCTGGACAACATCAAGCCTCCG
TCACTGATGGATGAGCTGCTGGACTCGATGATCAGTGTGGACAGCATTCAGTCTGAGGTGGCCGAGGGCG
AGCAGGACTGCAGCATGGCCACCACCATTTCCGTGTCCAACTATGAGACGGCTGCTTGCGATGATCAGAC
GATGACCGTGCTCCAAAGTTGTTTCGACGAGGATGAGGATGCCACAATGAATGACTACAGCTCGGCCGAG
TCCACACCCAAGCACGGATCTACTCCATCACCCAATCGTCGTTCCCTCACACCGAAAGAC

>In(3R)13-72_distal
CAACTAGCCCATCGTTCACCGCTAAGCCCAAAGCTTTTTGAGTTCCATGTTAATAAACAACACAAACAAA
TTATAACAATTTTCGTATTGATTGGGGAAAATATATCATAATAAATATGATTGCAAATATAACGACAGCA
ATGTCATATTACATGCTTTAATATGCAGAGCGGCGTTTAATTAGGTAATTAATACTTAAGATCGTTTATT
TCCTCTCAATGTTATCAGTTTCTCGTGGCGCTGACGACATGGCTAAGTATCGATTATTTTCTCGTGGGTT
TTGTTTTGCTCCGGTGCTACGACAACAAACCGGTTTCCAAACATAGAGTCACCCAGTTATTTTGTGGTGC
TTATCTAATTGGCCACGCTCAGCTCTGCTCAGTGCCTCTCAGTGGTGCTCATTTTGCGTCTTCCTCCCAT
GATTAAGTGCCATATGCCGTCGAGTTGGAGACGGCTACGCAAAATCAGTCGTATTCTAGCGCTGACAGGA
AGCAGACAGATACTCACCCAAGTATTAGCAACCAAAGGAGCAGCAATGGCAGAGGGAGATTCGAAATTTG
CCGGGCTGGAGTAGATTTTCCGCCAGAGCTCGCGCTTCGAACGCAATCATGGTCGAGTTTGTCATAATGG
AGGCACTCGTTCCATTAAATAATAGTCTTTGCGGCTCCAATCTA

>In(3L)62D:68A_proximal
AGAAGCTCTTTCGCAAATGGATTTTCGGGTTCGTCATTTAACTACAATATTTTGTGGGCCTTTCAAGGTT
CGTCTATTGTCCTGAAGGAAACTACGCAGAAGGCTTAAGTTTATTGTTTATTATGTGCGTTTTATGGCAC
GTTGTGCTTGTTTGCTTTTCCATTGCGTATTTAGAAGTTTATTAATCATACCTTTTACTACGACGACTTT
ATGAACATAAATTCTTGAAATTTGGAATTCCGATGAAACGAAAACAATACCTTTTTGTGAAGTTCAGGTA
AAGTAGAAAGAGTTCAATATCACAAAGAGCTGAACGATGACAGAAAGAGGTTTAAAGGATTTAGGCTTCA
GATACTTTTTGTTCATTTTTCAAGCTGAGGCATATAACCTGCTACTTTAAGTATTTTAATGGTTAGGCGC
CCTACCGACCTAATCTGTTCTTAATTTGTAAATTTTTGATCACTCGCCAACTCAGTCGCTTGCCATTAAA
ATCCCATTAAGTTTGAAGCTCAGACATATGAAAATTGAAAATTAAGTATACTGAAAGCTTTTTCATGCGC
AATACTAGTATACTAGACAATCAGCACATACATATGTACTTTAAAACATAAATATGTAAAGCATACTTAT
TCCACTCAAACAAAACTTTAATCAAATTAATCAAATTGCGCGCAAAAGAAGCTGTGAAAAACAAGTAAAA
AGTAAATGAGAACGCTAGAAAAAGTTCAAATTTTCAAAAGTGCGGCCGTTTTTTGGGCGTGGCAAGAAAG
AATCCGGAAACAATCTTGCGATGC

>In(3L)62D:68A_distal
GCTGCAATTGTACATCGTTCCATGTAATTTACTATAAATAACATTATATCTAATCGGACCGGGATTCAGG
CGCCTCCGAACCGTATTAGTTGCATTCATTGGCTGCAATTTGCATAATGATGCTGACTTTCGGGGGCACA
GACATCAAGCCGGGCTCAAGTCCCAATTATGTCTGCTACATTTTGTTTAATGCCCTCAATTTACCAGCTC
AAAGTTATCTCTGCAGCATGGAAAACTCCCCAGGCAAAGTCGAGTGAATTGCAATCCGTCGGTTGCCCAG
GATATTCGGCCAATTAGTTTCCTTGTGTGGGCTGCTTCTGCAGTAGACCCCCCCCCCCCTTCCTCCTCGC
ATTCGTCACACGGCACGTATGCGCAAGGTAAAACAATTTACGCTCAACTGGTTTCTAGCCCCATGCAACA

GGTTATTCAAAAATAATATACAAATCTATGCATAAAGCTCAAGTATTCTTTTGTTAAAATCCCTTTTTAA
CGAATTATTTTACCTACGGGTTATAGGTTTAAAAAACAAAAGTATTTGTGGGGGGGTGTAATATGACCTG
GGTCAAAACATAACAGCGAAACACTCCAAGCTTACAAAGCATTTATACAAATTTTTTTTTAGCAAAATTT
AACAATTAGCTTCAATTTCTGTTGGAATTTTTAAGTTTTAACCCATATTCTTAAAACCAAACGATCAGCT
AAATCTCTAAACTTGCAAGCAAAGCAATCCAAGTGA

>In(2R)NS
GCCACTTCGATTCTGCAAAGTCCTGTGTTCTACTGCCCCATCGCTAGCCATTCGAAGCTCAATATTTACT
CTGCCCCACAAAGAGGCGGGAAAGCCAAAGACGTGTTCGTGGCCCCAAACAAACAGACTGTCAACAGTTT
AAAGTTTTCATTGGGGCGAGCCATCATTGTTATCACTACTTAAATGGACCAGGATGAGGATCCATTTGTG
ATTGAGCGACGTCGCAATCGGCTGCGCGTGGAGACAGTTAGAGCGCAGCTCAATGCTCTTCTGGCACAAC
CCATCTTCCCCAGAGGCTTCAGCTTCAAGTATCCCAACAGCGAGGCGGCCCAATTAGCCACGAGTCATAC
TCAAAGTGCCGTGGAGACCATGAAGGCGGCCATAGAGGACCAGAAGCAGGCCAAAAAAGATCGCAACAAG
CGAAAACGGAATGCCTAATCTAACTTAAAGCTGTTTTATTGAATAGGAGATACAAAGCTATAGCCTTGGA
TGTTTCCATCAATGCTTTGAAACAAAGTATCGCACACCTCCATCGGTCAATGAGCGAGCGTGCTTGGCCA
ATAGTGTGGAAACGGACAAGTTTGGGGTGGTAAAGCACCTAGTTATAAAAACGAAAATCAAATCAAAAAC
AATCTAGATATGTGTTCATCAATACTTACTCTATGTACGGTTCCATTTCCTCCATCGTCCATCTCTTCTT
AGTCTTAAATAGCGCCCGCATGCGATCGCTTATATTGGTGGGCAGTAACTCCTCCGCCAGGGAACGTATG
CAAGGCTGCGCTCCCTCCTTGTCGCAAATGCCCAGGCCACGTAGATACTTTAAGTCGCAGGACATGCCCT
CGGGCAGTGCCTCCTGCCAGGTGCGCATGAACTCCTCGTTGCGAAAGCGCAAACCCGGCTGAAGAATGTT
CTGTGCCACAATGCGGGAGACCAAGGACTCCTGGTACTCAAACTTGCCCG

>In(3L)P
GCTGGTTTAATGGCTTCGACTGTATCAGACGCTGGATTTCTTCCAGATCCTGGGATAGAGTGGGTGGTGG
AGCCAAGGTAGCTGGCGGGCCCGGCGCCAACAGACCAACAGTTGGAGGCACCAACTGAGGAGACCACAGT
GCCAGGGATCCACTGGCCAGGGTAATCAGGAGAAGCCAATCCAGAGCGATGGCCATATCTGTAAGGTGGT
GACCGAAAGAAGACTAACAAAATTAAAGTCGCAGAAGCTTTTATATCCATCCATCAAGATTTGTTATTAC
TGTCTACTGATAGCGTCTTCCTGAATTTGGCGCAGCGTTTCTGTTGACCATTGTCGTCATCTGATAAGCG
CTTTTCGATCTGTTTATGGGTCTACCCAATTTTAAATCCCCCACGGAAGTCAGAGTTTTGGGATAAACTG
GCGGGTTGGTAAACATTTGGAGAGAAAGTATGCGAATTTATAACATGCTAAGCTTTATTTTCCAAGTGAA
TCACCAACAATCCGGATGGACCACATAGAACCGCAAACGAACACTTAATAACATTAGGATTTACATAGCT
CCTCATACATACACAAAGCAAACCCAAACTGGGGATTTATAGGATTCATCTTTTTTCGATTTTAAGAGAT
GATGACAAGTTAGTTATCCTTGCGAAATGGGCTTTGGAAAACGGATGATCGCAGTTAATGTTAATGAAAG
TTATGTCTTAGGTGGGCAGTGGCCACCACACTCGGTCAGTTGGCCATTTGGGCATTTGCCGAGTTGTGGG
GGGAGTGGGGTGTGCTGGTATTTTCTGGCCAAAAAGCTGGCCGAAGACAAAGCGAATCAGC

>In(3R)K_proximal
CTCATTTACCCGTGGTCAGCTAAAATGTCACCCATGGCAATGGTAACAGAGAAAAGCTCAAAGCTGGTCA
TAAACTTTATACTTGTTCATACTGTATGTATGAGACGTATGAATTGCAGAGTTGAGTGAATATTTAAATT
CAACAGTTGGGCATGCAAATTGCTCAGAATTCTCAAATATTTGCTTTTAATTTCCATGTCATGCATATTT
TGTGCAGCATTTAAATGAATGCATTTAAATGATTGCAACAGTTCCTTAAAATTTTGAAATGACTCACTTA
ACTAAGCTTAACTACAAGGCCAGGCCAATACGGTTTGGCACTGATTAAAATTTTGTTAAAAACTTTTAAG
TCCTGATGCGATCTACATTATGTGAAAAAATAAATAAAAGTTTTTATGGTTATGCAAAAAATGGGACACT
GGAGATGATTATCGGTTTGTTTGTCTGTGCCGCTTTTTCAGTTTTTTATATCCAACTCCGAGCCATAACA
AAACAATTTTGTTTACCCTCTGTTGTCTGACCCACTCTCCACTTGGATACTCAAACAGGCGACGCTGAAA
ACAGCCCGATGCCTCGGGGAAATCTCTGGGAAACTAGGAAAATGTCGGAACGGGATGTAATAGCTGGCTG
AGCTGAATGAATTTGCCCCATTTATCCACTAGAAACCAATCGAGATTGTTTAGCCGGCTTCTCCCAATTG
TAATGAAGAAAACTCGAGAATCGTTTTCAACATGGAAAAATGCGTACTTGTGGTTTTCGTCCTGATTGTG
GTAATGGGAAGAGTATATCTCTATGAAAGTGTCTTTTTCTTTGACCTTCTTTCGTGTATGCCC

>In(X)Be
GCTGCGTCTACTTGCTGTTGTTGTTGTTGATTTTCATCGGCTTTAATGCCTTTGTCTATTTTGGGTTTCT
GTTTATGGCGTGGGTTTTTCTCAATTGCATTTTTGGGCGCGCTCTCGACTGCAGCAGCAAAAGCACAGTG
GCGGGCAGAGAGATAGCAGCGTTTAAGAAATTAAGAAATATGCATATAGAAAATCGCCTAAAAACTGAAA
GAATTTAATACCCTAAAGAGGTGCTGTTTGATTCATTTTTAAATGTATAAATTGGATACGCTTTGCTATT
ATTTTGTCCTATTTTTCATCTTTAACGTGATACACATTGATTATATTAGTTCTAGACCATGAAATTCTCA
AATTTTAAAGACGTACGCTTTAAATGTACCATGTTAATTAGTTATTCTCTGGCAAAATGTAACAAAATTG
GAAACTACCATATTGACGGGCATTGTAAGTGTCGTATGTGTGAGTGTGGAAATACCAGAATTACCTAAAT
AACAACAAGAATAGCCGTGCACTTATAAAGCAATGTTTGTAATCGGATCGCATTGGCATGTTGGCCAGTT
GAGTTCAGTTCTCAATTGCTGGCCAAAAGTTTTTGGCCCCTCGAGTGTCTGACTGTGGACACGTTCTTCT
TTTTGAGTGGCCTCCTGATGGTGGTGATAGCTCTAAGAGCAATGGAAAGGTACGACTCTTTGGACTTAAG
CAACATGCACGGTGTCATTTAGACGGGTTTTGGAAACAAAGATTATTACTTCCTATTGACCCATTTGCAG
AACCAAAGGAAAGCTCAATGTGTCCCTGA

Table S1 Data Files

Contents	File Type	File Name(s)
Mapped reads	bam	<line>_GDL_WGS.bam*
Variant sites and genotypes	vcf	GDL_Chr<#>_SNPs.vcf*<br>GDL_Chr<#>_Indels.vcf*
Uncallable genome intervals	bed	GDL_uncallable_intervals.bed*
Regions IBD between populations	bed	GDL_IBD_regions.bed*
Heterozygous blocks	bed	GDL_HetBlocks.bed*
Multi-species alignment	MAF	view at http://genome-mirror.cshl.edu/

* SRA #SRP050151

Table S2 Read Counts by Line and by Chromosome

	**X**		**2L**		**2R**		**3L**		**3R**		**4**		**Other**	**unmapped**	
Line	Mapped Reads	*Avg DoC*	Mapped Reads	*Avg DoC*	Mapped Reads	*Avg DoC*	Mapped Reads	*Avg DoC*	Mapped Reads	*Avg DoC*	Mapped Reads	*Avg DoC*	Mapped Reads	# Reads	All reads
**Beijing, China**															
B04	3,036,688	*13.5*	3,294,733	*14.2*	3,020,949	*14.1*	3,469,105	*14.0*	4,012,220	*14.2*	212,663	*15.7*	3,690,254	1,725,808	22,462,420
B05	2,975,969	*13.2*	3,199,230	*13.8*	3,017,967	*14.1*	3,391,466	*13.7*	3,955,448	*14.0*	190,030	*14.0*	3,696,277	1,802,635	22,229,022
B10	3,006,384	*13.3*	3,249,286	*14.0*	2,992,388	*14.0*	3,420,409	*13.8*	3,958,669	*14.0*	201,815	*14.9*	4,363,567	1,583,002	22,775,520
B11	2,874,138	*12.7*	3,102,107	*13.3*	2,881,317	*13.5*	3,273,001	*13.2*	3,799,545	*13.5*	178,789	*13.2*	3,467,321	947,860	20,524,078
B12	3,117,248	*13.8*	3,387,218	*14.6*	3,107,037	*14.5*	3,588,896	*14.5*	4,128,763	*14.7*	215,253	*15.9*	3,988,871	1,042,874	22,576,160
B14	2,866,674	*12.7*	3,100,647	*13.3*	2,903,760	*13.6*	3,263,524	*13.2*	3,815,092	*13.5*	172,172	*12.7*	3,821,257	992,134	20,935,260
B23	2,680,424	*11.9*	2,885,414	*12.4*	2,703,854	*12.7*	3,052,229	*12.3*	3,565,008	*12.6*	163,975	*12.1*	2,938,173	1,500,197	19,489,274
B28	2,969,053	*13.1*	3,182,360	*13.7*	2,978,186	*13.9*	3,361,246	*13.6*	3,956,731	*14.0*	191,390	*14.1*	3,543,061	1,613,391	21,795,418
B38	2,967,684	*13.1*	3,188,412	*13.7*	2,985,929	*14.0*	3,376,369	*13.6*	3,911,689	*13.9*	185,567	*13.7*	3,511,111	1,002,299	21,129,060
B42	2,888,902	*12.8*	3,095,855	*13.3*	2,914,346	*13.6*	3,288,477	*13.3*	3,821,217	*13.6*	175,070	*12.9*	3,451,671	1,300,870	20,936,408
B43	2,836,306	*12.6*	3,050,177	*13.1*	2,857,044	*13.4*	3,231,038	*13.0*	3,754,890	*13.3*	173,324	*12.8*	3,407,559	1,351,016	20,661,354
B51	2,718,890	*12.0*	2,909,627	*12.5*	2,709,346	*12.7*	3,080,824	*12.4*	3,563,731	*12.6*	162,530	*12.0*	3,464,387	1,470,791	20,080,126
B52	2,980,418	*13.2*	3,201,375	*13.8*	3,039,420	*14.2*	3,400,459	*13.7*	3,979,801	*14.1*	170,940	*12.6*	3,250,693	1,045,958	21,069,064
B54	3,081,805	*13.7*	3,311,988	*14.3*	3,064,437	*14.4*	3,522,141	*14.2*	4,054,104	*14.4*	211,039	*15.6*	4,056,469	1,066,665	22,368,648
B59	2,297,710	*10.2*	2,658,126	*11.5*	2,250,237	*10.6*	2,774,998	*11.2*	3,013,778	*10.7*	219,460	*16.2*	3,974,267	321,486	17,510,062
**Ithaca, NY; North America**															
I01	3,226,159	*14.3*	3,508,949	*15.1*	3,214,396	*15.0*	3,713,192	*15.0*	4,260,913	*15.1*	250,279	*18.5*	4,353,981	2,120,995	24,648,864
I02	3,250,803	*14.4*	3,518,042	*15.1*	3,174,403	*14.9*	3,751,124	*15.1*	4,229,956	*15.0*	224,964	*16.6*	5,098,103	1,359,359	24,606,754
I03	3,337,483	*14.8*	3,622,081	*15.6*	3,286,862	*15.4*	3,833,662	*15.5*	4,370,088	*15.5*	233,148	*17.2*	4,186,246	1,638,022	24,507,592
I04	2,414,568	*10.7*	2,613,311	*11.2*	2,411,398	*11.3*	2,747,824	*11.1*	3,195,403	*11.3*	147,092	*10.8*	3,039,884	1,586,796	18,156,276
I06	2,296,416	*10.2*	2,683,927	*11.6*	2,312,681	*10.9*	2,795,719	*11.3*	3,079,207	*11.0*	191,818	*14.2*	3,429,044	1,300,110	18,088,922
I07	2,953,381	*13.1*	3,194,076	*13.7*	2,973,494	*13.9*	3,369,990	*13.6*	3,920,546	*13.9*	178,112	*13.1*	3,698,312	1,606,341	21,894,252
I13	3,341,466	*14.8*	3,614,755	*15.6*	3,274,832	*15.3*	3,826,004	*15.4*	4,364,808	*15.5*	233,200	*17.2*	4,884,146	2,490,161	26,029,372
I16	2,188,470	*9.7*	2,515,281	*10.9*	2,148,538	*10.1*	2,653,293	*10.7*	2,877,838	*10.3*	195,850	*14.5*	3,537,036	882,012	16,998,318
I17	3,480,184	*15.4*	3,771,246	*16.2*	3,381,971	*15.8*	3,978,544	*16.0*	4,500,611	*16.0*	251,956	*18.6*	4,348,931	2,175,781	25,889,224
I22	3,078,020	*13.6*	3,334,031	*14.3*	3,086,353	*14.4*	3,525,120	*14.2*	4,071,864	*14.5*	200,487	*14.8*	3,727,401	1,825,552	22,848,828
I23	3,400,043	*15.1*	3,681,958	*15.8*	3,378,539	*15.8*	3,914,561	*15.8*	4,500,820	*16.0*	220,345	*16.2*	3,971,172	1,726,272	24,793,710
I24	3,051,144	*13.5*	3,279,481	*14.1*	3,069,162	*14.4*	3,470,800	*14.0*	4,005,999	*14.2*	183,884	*13.5*	3,794,857	1,454,109	22,309,436
I26	2,336,786	*10.4*	2,691,791	*11.6*	2,344,720	*11.0*	2,796,824	*11.3*	3,125,576	*11.1*	189,769	*14.0*	3,421,484	320,876	17,227,826
I29	3,476,520	*15.4*	3,782,781	*16.3*	3,509,444	*16.4*	3,990,293	*16.1*	4,611,204	*16.4*	223,300	*16.4*	3,599,386	1,130,154	24,323,082
I31	2,685,643	*11.9*	2,864,483	*12.3*	2,683,780	*12.6*	3,015,219	*12.2*	3,520,315	*12.5*	159,047	*11.7*	3,065,260	1,450,553	19,444,300
I33	2,631,419	*11.7*	2,805,240	*12.1*	2,654,000	*12.4*	2,978,415	*12.0*	3,476,429	*12.3*	155,529	*11.5*	2,808,188	807,922	18,317,142

Table S2 Read Counts by Line and by Chromosome *cont.*

	X		2L		2R		3L		3R		4		Other	Unmapped	
Line	Mapped Reads	*Avg DoC*	Mapped Reads	*Avg DoC*	Mapped Reads	*Avg DoC*	Mapped Reads	*Avg DoC*	Mapped Reads	*Avg DoC*	Mapped Reads	*Avg DoC*	Mapped Reads	# Reads	All reads
**Ithaca, NY; North America** *cont.*															
I34	2,605,581	*11.5*	2,798,179	*12.0*	2,631,284	*12.3*	2,957,573	*11.9*	3,473,913	*12.3*	149,338	*11.0*	3,103,483	1,429,141	19,148,492
I35	2,650,204	*11.8*	2,889,464	*12.4*	2,688,141	*12.6*	3,038,748	*12.3*	3,541,389	*12.6*	163,575	*12.0*	3,050,198	1,098,427	19,120,146
I38	3,046,933	*13.5*	3,274,762	*14.1*	3,065,928	*14.3*	3,461,098	*14.0*	4,060,443	*14.4*	180,612	*13.3*	3,419,112	924,514	21,433,402
**Netherlands, Europe**															
N01	2,672,621	*11.9*	2,879,436	*12.4*	2,585,067	*12.1*	3,057,652	*12.3*	3,451,466	*12.3*	188,812	*13.9*	3,150,412	1,231,938	19,217,404
N02	2,885,047	*12.8*	3,099,663	*13.4*	2,793,423	*13.1*	3,280,875	*13.3*	3,719,541	*13.2*	201,465	*14.8*	3,417,667	1,246,813	20,644,494
N03	2,643,251	*11.7*	2,836,132	*12.2*	2,549,983	*11.9*	3,029,124	*12.2*	3,403,174	*12.1*	179,145	*13.2*	3,342,778	1,643,029	19,626,616
N04	2,714,598	*12.0*	2,906,524	*12.5*	2,616,973	*12.3*	3,115,407	*12.6*	3,504,246	*12.5*	188,258	*13.9*	3,241,832	1,581,186	19,869,024
N07	2,614,345	*11.6*	2,794,792	*12.0*	2,526,219	*11.8*	2,972,442	*12.0*	3,377,459	*12.0*	183,773	*13.5*	3,306,395	2,623,703	20,399,128
N10	2,762,048	*12.2*	2,966,830	*12.8*	2,671,331	*12.5*	3,141,768	*12.7*	3,567,745	*12.7*	194,015	*14.3*	3,365,200	2,774,081	21,443,018
N11	2,561,775	*11.4*	2,739,439	*11.8*	2,455,598	*11.5*	2,910,080	*11.7*	3,275,421	*11.6*	177,038	*13.0*	3,008,986	2,430,285	19,558,622
N13	2,007,332	*8.9*	2,178,327	*9.4*	1,944,961	*9.1*	2,302,586	*9.3*	2,589,275	*9.2*	139,667	*10.3*	2,953,696	2,888,632	17,004,476
N14	2,556,803	*11.3*	2,778,505	*12.0*	2,493,331	*11.7*	2,960,652	*11.9*	3,324,314	*11.8*	179,060	*13.2*	3,346,798	2,598,831	20,238,294
N15	2,140,351	*9.5*	2,305,988	*9.9*	2,060,129	*9.6*	2,443,834	*9.9*	2,764,036	*9.8*	153,206	*11.3*	2,792,190	2,385,130	17,044,864
N16	2,657,923	*11.8*	2,879,808	*12.4*	2,590,886	*12.1*	3,025,148	*12.2*	3,467,536	*12.3*	196,403	*14.5*	3,510,830	2,694,080	21,022,614
N17	2,720,721	*12.1*	2,912,528	*12.5*	2,633,445	*12.3*	3,104,319	*12.5*	3,538,345	*12.6*	190,261	*14.0*	3,546,928	1,188,533	19,835,080
N18	2,706,424	*12.0*	2,860,701	*12.3*	2,572,061	*12.1*	3,041,970	*12.3*	3,435,187	*12.2*	193,772	*14.3*	3,509,259	1,133,088	19,452,462
N19	2,545,409	*11.3*	2,721,587	*11.7*	2,472,318	*11.6*	2,918,159	*11.8*	3,292,900	*11.7*	178,014	*13.1*	3,378,758	1,801,793	19,308,938
N22	2,594,926	*11.5*	2,763,158	*11.9*	2,527,215	*11.8*	2,960,637	*12.0*	3,371,385	*12.0*	181,036	*13.3*	3,638,679	1,377,154	19,414,190
N23	2,680,173	*11.9*	2,872,055	*12.4*	2,592,838	*12.1*	3,072,860	*12.4*	3,463,906	*12.3*	191,237	*14.1*	3,806,792	1,297,673	19,977,534
N25	2,483,169	*11.0*	2,653,241	*11.4*	2,380,018	*11.2*	2,814,972	*11.4*	3,182,260	*11.3*	170,859	*12.6*	2,986,125	1,695,424	18,366,068
N29	2,394,677	*10.6*	2,560,181	*11.0*	2,313,070	*10.8*	2,736,354	*11.1*	3,096,088	*11.0*	171,944	*12.7*	2,879,955	876,081	17,028,350
N30	2,601,721	*11.5*	2,810,593	*12.1*	2,524,842	*11.8*	2,962,690	*12.0*	3,343,543	*11.9*	185,376	*13.7*	3,098,593	1,311,580	18,838,938
**Tasmania, Australia**															
T01	2,609,342	*11.6*	2,806,398	*12.1*	2,538,444	*11.9*	2,963,810	*12.0*	3,377,590	*12.0*	187,856	*13.8*	3,187,477	1,596,685	19,267,602
T04	2,695,615	*11.9*	2,944,937	*12.7*	2,642,729	*12.4*	3,143,618	*12.7*	3,525,917	*12.5*	203,238	*15.0*	3,247,738	2,074,542	20,478,334
T05	2,212,527	*9.8*	2,393,906	*10.3*	2,163,829	*10.1*	2,536,623	*10.2*	2,890,343	*10.2*	155,773	*11.4*	2,921,670	2,073,635	17,348,306
T07	2,730,524	*12.1*	2,949,876	*12.7*	2,662,556	*12.4*	3,135,924	*12.6*	3,559,569	*12.6*	197,232	*14.5*	3,791,203	1,892,646	20,919,530
T09	2,614,884	*11.6*	2,836,286	*12.2*	2,575,365	*12.1*	3,007,487	*12.1*	3,433,549	*12.2*	191,110	*14.1*	3,399,748	1,592,453	19,650,882
T10	2,694,315	*11.9*	2,879,062	*12.4*	2,612,489	*12.2*	3,073,461	*12.4*	3,497,154	*12.4*	183,994	*13.5*	3,323,085	1,997,484	20,261,044
T14A	2,824,044	*12.5*	3,028,138	*13.0*	2,748,202	*12.8*	3,230,063	*13.0*	3,659,572	*13.0*	191,699	*14.1*	3,814,940	2,365,782	21,862,440
T22A	2,413,591	*10.7*	2,608,848	*11.2*	2,350,302	*11.0*	2,757,057	*11.1*	3,145,979	*11.1*	172,482	*12.6*	2,861,882	2,157,661	18,467,802
T23	2,084,131	*9.2*	2,255,754	*9.7*	2,026,514	*9.5*	2,399,362	*9.7*	2,720,426	*9.7*	138,360	*10.2*	2,615,391	2,707,958	16,947,896
T24	2,541,912	*11.3*	2,717,973	*11.7*	2,465,687	*11.5*	2,889,107	*11.7*	3,274,862	*11.6*	176,156	*13.0*	3,260,393	1,429,964	18,756,054
T25A	2,423,463	*10.7*	2,608,506	*11.2*	2,355,569	*11.0*	2,763,855	*11.1*	3,138,458	*11.1*	168,925	*12.4*	2,966,167	1,311,455	17,736,398

Table S2 Read Counts by Line and by Chromosome *cont.*

	X		2L		2R		3L		3R		4		Other	Unmapped	
Line	Mapped Reads	*Avg DoC*	Mapped Reads	*Avg DoC*	Mapped Reads	*Avg DoC*	Mapped Reads	*Avg DoC*	Mapped Reads	*Avg DoC*	Mapped Reads	*Avg DoC*	Mapped Reads	# Reads	All reads
**Tasmania, Australia** *cont.*															
T29A	2,726,239	*12.1*	2,934,365	*12.6*	2,632,961	*12.3*	3,106,046	*12.5*	3,539,226	*12.6*	193,836	*14.3*	3,228,284	2,408,727	20,769,684
T30A	2,750,016	*12.2*	2,970,764	*12.8*	2,668,337	*12.5*	3,133,105	*12.6*	3,558,955	*12.6*	193,316	*14.2*	3,331,261	1,208,494	19,814,248
T35	2,488,591	*11.0*	2,685,736	*11.6*	2,416,500	*11.3*	2,850,237	*11.5*	3,231,543	*11.5*	177,540	*13.1*	3,733,324	1,465,527	19,048,998
T36B	2,743,172	*12.2*	2,965,658	*12.8*	2,670,885	*12.5*	3,151,340	*12.7*	3,545,922	*12.6*	196,666	*14.5*	3,273,345	1,543,780	20,090,768
T39	2,725,523	*12.1*	2,924,030	*12.6*	2,637,324	*12.3*	3,111,731	*12.6*	3,523,461	*12.5*	190,848	*14.1*	3,437,056	1,411,785	19,961,758
T43A	2,154,291	*9.5*	2,317,445	*10.0*	2,078,454	*9.7*	2,459,230	*9.9*	2,775,729	*9.8*	152,223	*11.2*	2,621,449	2,902,111	17,460,932
T45B	2,330,496	*10.3*	2,529,899	*10.9*	2,337,976	*10.9*	2,693,901	*10.9*	3,090,043	*11.0*	149,005	*11.0*	2,992,793	1,560,437	17,684,550
**Zimbabwe, Africa**															
ZH23	2,575,459	*11.4*	2,766,549	*11.9*	2,533,300	*11.8*	2,959,179	*11.9*	3,383,858	*12.0*	188,712	*13.9*	3,439,165	1,804,756	19,650,978
ZH26	2,589,418	*11.4*	2,837,108	*12.2*	2,563,883	*12.0*	3,003,891	*12.1*	3,409,195	*12.1*	189,569	*13.9*	3,725,403	2,194,373	20,512,840
ZH33	2,649,472	*11.7*	2,856,989	*12.3*	2,727,304	*12.8*	3,031,176	*12.2*	3,579,097	*12.7*	151,893	*11.2*	3,130,759	1,104,706	19,231,396
ZH42	2,972,582	*13.2*	3,242,876	*13.9*	3,017,990	*14.1*	3,428,694	*13.8*	3,983,790	*14.1*	184,733	*13.6*	3,508,896	1,233,925	21,573,486
ZS10	2,559,281	*11.3*	2,790,297	*12.0*	2,603,958	*12.2*	2,946,016	*11.9*	3,430,725	*12.2*	159,830	*11.8*	3,152,941	1,067,284	18,710,332
ZW09	2,659,983	*11.8*	2,939,827	*12.6*	2,696,341	*12.6*	3,095,530	*12.5*	3,580,265	*12.7*	176,672	*13.0*	3,181,496	1,258,388	19,588,502
ZW139	2,729,548	*12.1*	3,024,063	*13.0*	2,795,696	*13.1*	3,222,144	*13.0*	3,708,748	*13.1*	177,553	*13.1*	3,033,429	1,308,819	20,000,000
ZW140	2,737,445	*12.1*	3,018,753	*13.0*	2,771,506	*13.0*	3,211,076	*12.9*	3,697,881	*13.1*	177,059	*13.0*	3,170,461	1,552,769	20,336,950
ZW142	2,751,245	*12.2*	3,015,481	*13.0*	2,758,944	*12.9*	3,212,675	*13.0*	3,692,034	*13.1*	181,030	*13.3*	3,415,925	972,666	20,000,000
ZW144	2,574,503	*11.4*	2,814,264	*12.1*	2,673,337	*12.5*	2,971,541	*12.0*	3,514,858	*12.5*	141,552	*10.4*	2,652,985	1,081,288	18,424,328
ZW155	2,693,520	*11.9*	2,959,209	*12.7*	2,721,907	*12.7*	3,130,876	*12.6*	3,619,366	*12.8*	172,586	*12.7*	3,013,541	1,878,607	20,189,612
ZW177	2,682,984	*11.9*	2,941,812	*12.6*	2,721,627	*12.7*	3,121,164	*12.6*	3,577,353	*12.7*	177,947	*13.1*	3,269,893	1,770,160	20,262,940
ZW184	2,730,523	*12.1*	3,001,890	*12.9*	2,806,159	*13.2*	3,158,435	*12.8*	3,673,716	*13.1*	162,684	*12.0*	3,122,647	1,721,408	20,377,462
ZW185	2,677,725	*11.8*	2,906,698	*12.5*	2,740,756	*12.8*	3,070,819	*12.4*	3,607,400	*12.8*	168,127	*12.4*	2,912,457	1,390,146	19,474,128

Table S3 Variant Calls by Line and Chromosome

	X		2L		2R		3L		3R		4	
**Line**	**SNP**	***indel***	**SNP**	***indel***	**SNP**	***indel***	**SNP**	***indel***	**SNP**	***indel***	**SNP**	***Indel***
**Beijing, China**												
B04	96,318	*15,163*	140,046	*16,680*	123,435	*14,886*	139,281	*17,262*	129,823	*16,793*	825	*151*
B05	95,028	*13,580*	139,781	*15,351*	122,472	*13,782*	184,280	*23,033*	231,795	*36,114*	972	*139*
B10	96,583	*14,577*	139,105	*16,331*	124,632	*14,634*	140,353	*17,013*	177,103	*25,965*	1,031	*140*
B11	95,292	*12,667*	232,757	*29,217*	122,749	*12,780*	157,161	*19,884*	149,582	*19,042*	1,006	*114*
B12	96,931	*16,690*	246,150	*36,044*	123,565	*15,796*	138,549	*18,681*	140,716	*19,204*	911	*164*
B14	96,301	*12,623*	135,733	*13,748*	120,988	*12,608*	211,642	*30,884*	133,540	*15,284*	960	*116*
B23	93,915	*10,864*	138,564	*12,614*	122,560	*11,525*	225,754	*31,648*	210,687	*29,811*	907	*102*
B28	93,806	*12,479*	136,764	*14,247*	124,054	*13,005*	137,809	*14,556*	169,706	*25,171*	1,355	*147*
B38	95,178	*13,547*	135,447	*14,608*	122,236	*13,470*	202,217	*29,036*	188,075	*25,737*	930	*144*
B42	94,959	*12,785*	139,773	*14,230*	119,990	*12,852*	211,057	*29,178*	222,683	*30,807*	965	*148*
B43	95,024	*13,013*	137,936	*14,379*	118,903	*12,615*	213,921	*31,549*	200,852	*30,459*	974	*108*
B51	96,095	*11,967*	139,731	*12,931*	121,593	*11,616*	214,229	*30,990*	226,099	*31,692*	1,077	*138*
B52	94,071	*13,040*	137,891	*14,637*	124,429	*13,773*	220,342	*33,634*	192,926	*30,074*	892	*131*
B54	94,300	*15,674*	241,173	*33,728*	140,194	*19,329*	140,953	*18,656*	131,375	*17,647*	811	*139*
B59	86,094	*10,758*	200,582	*24,230*	145,049	*17,271*	134,297	*14,567*	147,570	*18,390*	897	*156*
**Ithaca, NY; North America**												
I01	88,664	*15,306*	249,227	*36,836*	189,734	*26,653*	144,946	*19,608*	155,496	*22,870*	913	*156*
I02	84,777	*15,814*	144,695	*19,048*	124,388	*16,335*	145,946	*21,597*	131,528	*18,608*	1,490	*321*
I03	84,162	*15,804*	143,713	*19,679*	120,766	*16,658*	141,783	*21,769*	187,773	*33,602*	1,624	*324*
I04	84,050	*7,852*	238,388	*26,262*	123,078	*9,474*	144,657	*10,613*	137,724	*10,518*	1,749	*176*
I06	82,358	*10,372*	237,733	*32,024*	127,834	*13,201*	155,295	*19,345*	131,383	*13,424*	1,324	*211*
I07	87,118	*11,914*	153,777	*17,297*	140,445	*17,193*	143,051	*15,811*	130,871	*15,312*	646	*91*
I13	85,410	*15,973*	156,026	*21,284*	132,284	*18,184*	142,227	*20,899*	134,053	*19,408*	775	*158*
I16	83,851	*10,177*	152,314	*15,977*	125,186	*11,469*	138,317	*14,306*	133,596	*12,813*	597	*119*
I17	84,606	*17,490*	146,275	*21,586*	126,100	*18,204*	165,328	*28,785*	211,233	*34,725*	1,883	*381*
I22	83,199	*13,527*	238,724	*33,614*	139,837	*18,175*	155,382	*21,570*	136,559	*17,555*	1,739	*261*
I23	85,522	*15,824*	177,750	*25,895*	137,867	*19,900*	181,574	*32,124*	171,608	*30,330*	1,745	*309*
I24	84,033	*12,846*	144,222	*16,064*	122,238	*13,860*	152,145	*19,237*	228,070	*35,924*	1,755	*258*
I26	82,050	*10,364*	171,419	*19,891*	132,505	*14,893*	147,016	*16,357*	218,586	*31,455*	1,753	*285*
I29	80,354	*16,008*	152,322	*22,276*	127,821	*18,993*	148,117	*23,215*	141,891	*22,349*	1,758	*336*
I31	85,686	*9,491*	147,215	*13,061*	121,298	*10,815*	139,516	*12,305*	133,632	*12,361*	623	*85*
I33	85,856	*8,722*	145,161	*12,128*	123,325	*10,357*	140,330	*11,427*	135,450	*12,108*	774	*77*
I34	84,360	*8,586*	145,356	*11,711*	123,620	*10,562*	138,855	*11,298*	132,567	*12,013*	1,749	*138*
I35	85,892	*10,011*	145,090	*13,108*	135,593	*14,410*	149,116	*15,333*	141,055	*14,825*	1,571	*212*
I38	84,257	*12,565*	144,793	*16,263*	126,387	*14,638*	140,211	*16,710*	132,167	*16,504*	376	*44*
**Netherlands, Europe**												
N01	84,069	*11,700*	135,973	*13,886*	118,272	*11,584*	136,113	*14,884*	130,973	*14,050*	1,053	*175*
N02	82,245	*12,656*	168,631	*21,948*	127,955	*15,404*	148,378	*19,396*	126,962	*15,498*	1,411	*258*
N03	81,964	*11,087*	149,558	*16,299*	128,168	*13,039*	137,960	*14,661*	131,503	*13,508*	1,751	*284*
N04	81,134	*11,783*	133,540	*14,019*	119,473	*12,324*	138,218	*15,596*	128,168	*14,230*	998	*155*
N07	81,946	*9,800*	246,489	*31,202*	123,149	*11,124*	135,300	*12,981*	130,656	*12,574*	1,801	*298*
N10	77,723	*9,766*	243,257	*33,545*	121,777	*11,897*	136,496	*13,899*	120,623	*11,468*	990	*153*
N11	83,581	*9,272*	140,051	*11,616*	121,243	*9,563*	144,441	*14,302*	210,892	*27,181*	809	*123*
N13	84,220	*4,947*	132,988	*5,999*	121,860	*5,407*	148,311	*9,046*	199,752	*20,535*	774	*86*
N14	84,593	*9,790*	152,629	*15,504*	172,495	*19,528*	137,079	*12,694*	130,165	*11,543*	1,332	*203*

Table S3 Variant Calls by Line and Chromosome *cont.*

	X		2L		2R		3L		3R		4	
**Line**	**SNP**	***indel***	**SNP**	***indel***	**SNP**	***indel***	**SNP**	***indel***	**SNP**	***indel***	**SNP**	***Indel***
**Netherlands, Europe** *cont.*												
N15	83,057	*5,568*	133,749	*7,117*	119,336	*6,022*	141,062	*7,777*	130,485	*7,068*	1,031	*124*
N16	82,112	*10,205*	138,845	*14,043*	125,061	*12,141*	179,650	*22,051*	134,816	*13,746*	1,345	*220*
N17	81,425	*11,925*	243,849	*32,932*	128,610	*14,260*	161,846	*22,448*	135,199	*16,049*	1,828	*304*
N18	83,648	*11,477*	144,991	*15,536*	122,774	*12,124*	140,244	*15,194*	130,026	*13,852*	1,343	*231*
N19	82,514	*10,083*	191,707	*16,895*	120,820	*10,827*	139,118	*14,282*	143,492	*15,257*	814	*121*
N22	81,720	*11,059*	195,296	*17,918*	120,232	*11,334*	139,630	*14,285*	151,230	*19,237*	1,423	*230*
N23	80,426	*11,353*	239,092	*31,771*	132,963	*14,816*	137,440	*15,303*	130,077	*13,672*	1,444	*272*
N25	80,597	*9,298*	142,909	*13,581*	130,935	*12,652*	141,292	*13,627*	139,295	*13,858*	1,501	*213*
N29	83,842	*9,376*	232,943	*28,224*	126,212	*11,076*	138,198	*12,380*	131,252	*11,722*	1,023	*146*
N30	82,121	*11,022*	136,796	*13,046*	122,019	*11,717*	138,389	*14,423*	131,620	*13,112*	599	*96*
**Tasmania, Australia**												
T01	88,545	*11,392*	145,059	*14,143*	122,432	*11,745*	151,573	*15,887*	136,473	*14,146*	1,103	*173*
T04	86,848	*11,101*	142,673	*13,912*	126,325	*12,060*	224,878	*33,979*	220,759	*31,879*	791	*126*
T05	87,228	*7,063*	144,979	*9,173*	124,532	*7,592*	155,479	*11,684*	140,118	*9,476*	1,438	*196*
T07	86,441	*11,323*	251,811	*34,275*	195,476	*26,509*	145,557	*15,383*	138,655	*14,549*	1,806	*306*
T09	86,890	*10,866*	158,923	*15,885*	128,359	*12,492*	218,547	*30,102*	143,221	*14,744*	1,163	*204*
T10	87,170	*10,909*	145,668	*14,084*	125,080	*11,760*	226,060	*34,057*	143,116	*15,564*	1,329	*212*
T14A	85,205	*11,828*	150,028	*15,715*	124,610	*12,898*	157,477	*19,788*	147,875	*17,411*	1,320	*236*
T22A	87,191	*8,733*	141,364	*10,596*	175,803	*18,811*	144,421	*11,516*	139,996	*10,908*	1,998	*312*
T23	86,362	*5,907*	138,913	*8,270*	129,441	*7,433*	130,381	*7,042*	115,406	*5,876*	564	*63*
T24	87,618	*10,481*	148,354	*13,305*	123,271	*10,754*	151,850	*15,397*	217,293	*29,344*	771	*126*
T25A	87,383	*9,420*	243,607	*29,635*	180,890	*20,908*	145,664	*12,846*	170,346	*14,275*	1,456	*209*
T29A	86,144	*11,723*	148,499	*14,880*	145,675	*13,984*	169,758	*17,687*	135,739	*14,168*	1,743	*287*
T30	87,253	*12,997*	148,957	*15,922*	125,375	*12,949*	217,955	*32,672*	219,363	*32,895*	1,777	*310*
T35	88,072	*10,311*	230,647	*27,284*	191,412	*23,869*	177,848	*16,575*	139,212	*12,766*	1,753	*240*
T36B	86,850	*12,201*	158,913	*18,006*	139,412	*16,064*	228,587	*35,919*	210,741	*30,101*	1,462	*271*
T39	86,450	*12,058*	147,384	*14,957*	144,614	*14,283*	147,689	*16,784*	192,150	*24,956*	803	*138*
T43A	87,807	*6,382*	152,276	*8,213*	176,200	*16,618*	151,405	*9,296*	195,388	*19,437*	985	*108*
T45B	88,761	*7,603*	151,777	*10,567*	122,916	*8,584*	146,190	*11,022*	217,157	*26,461*	1,508	*132*
**Zimbabwe, Africa**												
ZH23	171,276	*19,994*	204,521	*18,998*	146,959	*13,563*	203,705	*21,230*	253,696	*35,044*	1,720	*286*
ZH26	145,667	*14,911*	255,846	*28,755*	118,051	*10,202*	250,722	*33,502*	250,140	*33,002*	1,624	*249*
ZH33	153,164	*16,046*	220,937	*18,399*	160,023	*14,369*	257,568	*32,256*	235,131	*31,661*	1,657	*138*
ZH42	177,872	*26,159*	303,456	*39,819*	165,397	*20,274*	285,542	*42,409*	277,232	*42,613*	1,692	*261*
ZS10	173,895	*18,068*	296,140	*33,740*	223,932	*25,838*	201,038	*18,420*	271,514	*34,181*	1,663	*158*
ZW09	184,492	*22,328*	310,275	*37,906*	169,181	*18,243*	268,603	*35,164*	269,233	*34,219*	1,852	*266*
ZW139	183,555	*22,781*	220,721	*21,472*	178,604	*18,201*	288,917	*40,851*	285,369	*39,726*	1,881	*243*
ZW140	182,107	*22,612*	224,337	*22,359*	173,788	*17,491*	289,355	*40,001*	285,229	*38,391*	1,796	*247*
ZW142	185,220	*24,332*	211,945	*22,398*	173,675	*18,481*	282,061	*38,771*	274,969	*37,558*	1,718	*267*
ZW144	183,630	*19,141*	222,452	*19,041*	170,538	*15,981*	281,612	*34,034*	281,479	*35,442*	1,684	*139*
ZW155	175,922	*20,577*	217,761	*20,034*	167,308	*15,895*	283,317	*39,027*	210,343	*23,406*	1,620	*210*
ZW177	185,795	*22,245*	226,623	*22,010*	172,763	*17,103*	286,148	*38,347*	284,316	*37,910*	1,750	*237*
ZW184	153,937	*17,836*	150,410	*14,736*	118,980	*12,130*	141,061	*14,189*	134,517	*14,362*	608	*82*
ZW185	181,366	*19,617*	305,132	*35,499*	174,455	*17,357*	255,225	*30,194*	250,399	*29,334*	1,693	*193*

Table S4 Genotypes of Known Large Inversions

Chrom	2L	2R	3L	3L	3R	3R	3R	3R	X	X
Inv Name	In(2L)t	In(2R)NS	In(3L)P	In(3L) 62D:68A	In(3R)K	In(3R)P	In(3R)Mo	In(3R) 13-72	X(Be)	X(A)
Ref.	*1*	*2*	*2*	*3*	*2*	*4*	*2*	*3*	*2*	*2*
**Beijing, China**										
B04	0/0	0/0	0/0	0/0	0/0	0/0	0/0	0/0	0/0	0/0
B05	0/0	0/0	**1/1**	0/0	0/0	**0/1**	0/0	0/0	0/0	0/0
B10	0/0	0/0	0/0	0/0	0/0	0/0	0/0	0/0	0/0	0/0
B11	**0/1**	0/0	0/0	0/0	0/0	0/0	0/0	0/0	0/0	0/0
B12	**0/1**	0/0	0/0	0/0	0/0	0/0	0/0	0/0	0/0	0/0
B14	0/0	0/0	**0/1**	0/0	0/0	0/0	0/0	0/0	0/0	0/0
B23	0/0	0/0	**0/1**	0/0	0/0	**0/1**	0/0	0/0	0/0	0/0
B28	0/0	0/0	0/0	0/0	0/0	0/0	**0/1**	0/0	0/0	0/0
B38	0/0	0/0	**0/1**	0/0	0/0	0/0	0/0	0/0	0/0	0/0
B42	0/0	0/0	**0/1**	0/0	0/0	**0/1**	0/0	0/0	0/0	0/0
B43	0/0	0/0	**0/1**	0/0	0/0	0/0	0/0	0/0	0/0	0/0
B51	0/0	0/0	**0/1**	0/0	0/0	**0/1**	0/0	0/0	0/0	0/0
B52	0/0	0/0	**0/1**	0/0	0/0	0/0	0/0	0/0	0/0	0/0
B54	**0/1**	0/0	0/0	0/0	0/0	0/0	0/0	0/0	0/0	0/0
B59	**0/1**	0/0	0/0	0/0	0/0	0/0	0/0	0/0	0/0	0/0
**Ithaca, NY; North America**										
I01	**0/1**	**0/1**	0/0	0/0	0/0	0/0	**0/1**	0/0	0/0	0/0
I02	0/0	0/0	0/0	0/0	0/0	0/0	**1/1**	0/0	0/0	0/0
I03	0/0	0/0	0/0	0/0	0/0	0/0	**0/1**	0/0	0/0	0/0
I04	**0/1**	0/0	0/0	0/0	0/0	0/0	0/0	0/0	0/0	0/0
I06	**0/1**	0/0	0/0	0/0	0/0	0/0	0/0	0/0	0/0	0/0
I07	0/0	0/0	0/0	0/0	0/0	0/0	0/0	0/0	0/0	0/0
I13	0/0	0/0	0/0	0/0	0/0	0/0	0/0	0/0	0/0	0/0
I16	0/0	0/0	0/0	0/0	0/0	0/0	0/0	0/0	0/0	0/0
I17	0/0	0/0	0/0	0/0	**0/1**	0/0	0/0	0/0	0/0	0/0
I22	**0/1**	0/0	0/0	0/0	0/0	0/0	0/0	**1/1**	0/0	0/0
I23	0/0	0/0	0/0	0/0	0/0	0/0	0/0	0/0	0/0	0/0
I24	0/0	0/0	0/0	0/0	0/0	**0/1**	0/0	0/0	0/0	0/0
I26	0/0	0/0	0/0	0/0	**0/1**	0/0	0/0	0/0	0/0	0/0
I29	0/0	0/0	0/0	0/0	0/0	0/0	0/0	0/0	0/0	0/0
I31	0/0	0/0	0/0	0/0	0/0	0/0	0/0	0/0	0/0	0/0
I33	0/0	0/0	0/0	0/0	0/0	0/0	0/0	0/0	0/0	0/0
I34	0/0	0/0	0/0	0/0	0/0	0/0	0/0	0/0	0/0	0/0
I35	0/0	0/0	0/0	0/0	0/0	0/0	0/0	0/0	0/0	0/0
I38	0/0	0/0	0/0	0/0	0/0	0/0	0/0	0/0	0/0	0/0
**Netherlands, Europe**										
N01	0/0	0/0	0/0	0/0	0/0	0/0	0/0	0/0	0/0	0/0
N02	0/0	0/0	0/0	0/0	0/0	0/0	0/0	0/0	0/0	0/0
N03	0/0	0/0	0/0	0/0	0/0	0/0	0/0	0/0	0/0	0/0
N04	0/0	0/0	0/0	0/0	0/0	0/0	0/0	0/0	0/0	0/0
N07	**0/1**	0/0	0/0	0/0	0/0	0/0	0/0	0/0	0/0	0/0
N10	**0/1**	0/0	0/0	0/0	0/0	0/0	0/0	0/0	0/0	0/0
N11	0/0	0/0	0/0	0/0	0/0	**0/1**	0/0	0/0	0/0	0/0
N13	0/0	0/0	0/0	0/0	0/0	0/0	0/0	0/0	0/0	0/0
N14	0/0	0/0	0/0	0/0	0/0	0/0	0/0	0/0	0/0	0/0

Table S4 Genotype of Known Large Inversions *cont.*

Chrom	2L	2R	3L	3L	3R	3R	3R	3R	X	X
Inv Name	In(2L)t	In(2R)NS	In(3L)P	In(3L) 62D:68A	In(3R)K	In(3R)P	In(3R)Mo	In(3R) 13-72	X(Be)	X(A)
Ref.	*1*	*2*	*2*	*3*	*2*	*4*	*2*	*3*	*2*	*2*
**Netherlands, Europe** *cont.*										
N15	0/0	0/0	0/0	0/0	0/0	0/0	0/0	0/0	0/0	0/0
N16	0/0	0/0	**0/1**	0/0	0/0	0/0	0/0	0/0	0/0	0/0
N17	**0/1**	0/0	0/0	0/0	0/0	0/0	0/0	0/0	0/0	0/0
N18	0/0	0/0	0/0	0/0	0/0	0/0	**1/1**	0/0	0/0	0/0
N19	**1/1**	0/0	0/0	0/0	0/0	0/0	0/0	0/0	0/0	0/0
N22	**1/1**	0/0	0/0	0/0	0/0	0/0	**0/1**	0/0	0/0	0/0
N23	**0/1**	0/0	0/0	0/0	0/0	0/0	0/0	0/0	0/0	0/0
N25	0/0	0/0	0/0	0/0	0/0	0/0	0/0	0/0	0/0	0/0
N29	**0/1**	0/0	0/0	0/0	0/0	0/0	0/0	0/0	0/0	0/0
N30	0/0	0/0	0/0	0/0	0/0	0/0	0/0	0/0	0/0	0/0
**Tasmania, Australia**										
T01	0/0	0/0	0/0	0/0	0/0	0/0	0/0	0/0	0/0	0/0
T04	0/0	0/0	**0/1**	0/0	0/0	**0/1**	0/0	0/0	0/0	0/0
T05	0/0	0/0	0/0	0/0	0/0	0/0	0/0	0/0	0/0	0/0
T07	**0/1**	**0/1**	0/0	0/0	0/0	0/0	0/0	0/0	0/0	0/0
T09	0/0	0/0	**0/1**	0/0	0/0	0/0	0/0	0/0	0/0	0/0
T10	0/0	0/0	**0/1**	0/0	0/0	0/0	0/0	0/0	0/0	0/0
T14A	0/0	0/0	0/0	0/0	0/0	0/0	0/0	0/0	0/0	0/0
T22A	0/0	**0/1**	0/0	0/0	0/0	0/0	0/0	0/0	0/0	0/0
T23	0/0	0/0	0/0	0/0	0/0	0/0	0/0	0/0	0/0	0/0
T24	0/0	0/0	0/0	0/0	0/0	**0/1**	0/0	0/0	0/0	0/0
T25A	**0/1**	**0/1**	0/0	0/0	0/0	0/0	0/0	0/0	0/0	0/0
T29A	0/0	**0/1**	**1/1**	0/0	0/0	0/0	0/0	0/0	0/0	0/0
T30A	0/0	0/0	**0/1**	0/0	0/0	**0/1**	0/0	0/0	0/0	0/0
T35	0/0	**0/1**	**1/1**	0/0	0/0	0/0	0/0	0/0	0/0	0/0
T36B	0/0	0/0	**0/1**	0/0	0/0	0/0	0/0	0/0	0/0	0/0
T39	0/0	**0/1**	0/0	0/0	0/0	0/0	0/0	0/0	0/0	0/0
T43A	0/0	**0/1**	0/0	0/0	0/0	**0/1**	0/0	0/0	0/0	0/0
T45B	0/0	0/0	0/0	0/0	0/0	**0/1**	0/0	0/0	0/0	0/0
**Zimbabwe, Africa**										
ZH23	**1/1**	0/0	0/0	0/0	0/0	0/0	**0/1**	0/0	**1/1**	0/0
ZH26	**0/1**	0/0	0/0	0/0	**0/1**	**0/1**	0/0	0/0	0/0	**0/1**
ZH33	0/0	0/0	0/0	**0/1**	**0/1**	0/0	0/0	0/0	0/0	0/0
ZH42	**0/1**	0/0	0/0	**0/1**	0/0	**0/1**	0/0	0/0	0/0	0/0
ZS10	**0/1**	**0/1**	0/0	0/0	**0/1**	0/0	0/0	0/0	0/0	0/0
ZW09	**0/1**	0/0	0/0	**0/1**	0/0	**0/1**	0/0	0/0	0/0	0/0
ZW139	0/0	0/0	0/0	**0/1**	0/0	**0/1**	0/0	0/0	0/0	0/0
ZW140	0/0	0/0	0/0	**0/1**	0/0	**0/1**	0/0	0/0	0/0	0/0
ZW142	0/0	0/0	0/0	**0/1**	0/0	**0/1**	0/0	0/0	0/0	0/0
ZW144	0/0	0/0	0/0	**0/1**	0/0	**0/1**	0/0	0/0	0/0	0/0
ZW155	0/0	0/0	0/0	**0/1**	0/0	0/0	0/0	0/0	0/0	0/0
ZW177	0/0	0/0	0/0	**0/1**	0/0	**0/1**	0/0	0/0	0/0	0/0
ZW184	0/0	0/0	0/0	0/0	0/0	0/0	0/0	0/0	0/0	0/0
ZW185	**0/1**	0/0	0/0	**0/1**	0/0	**0/1**	0/0	0/0	0/0	0/0

1. Andolfatto *et al.* 1999 2. Corbett-Detig *et al.* 2012 3. This study 4. Sezgin *et al.* 2004

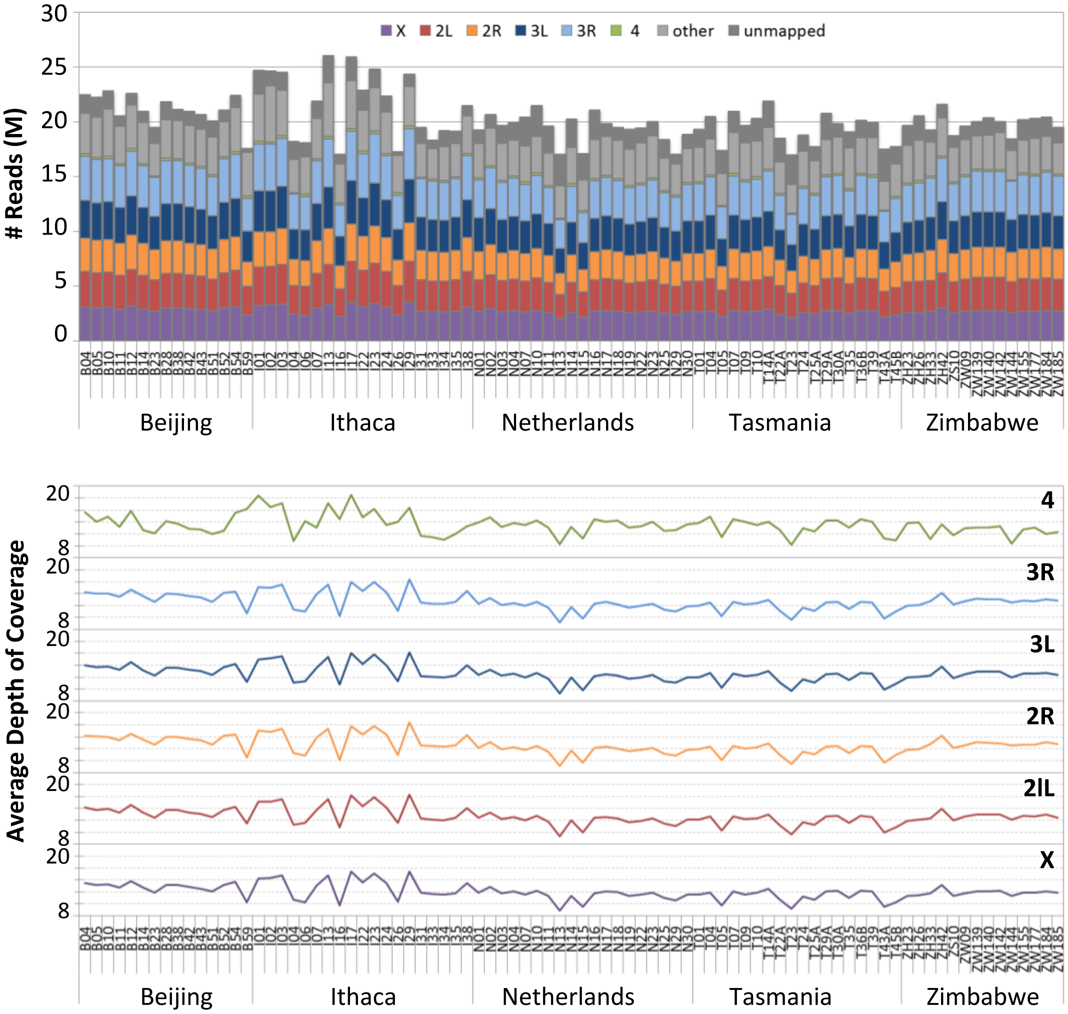

Figure S1 Read Counts and Depth of Coverage by Chromosome
(**Top**) The number of reads mapped to the reference genome is shown for each *Drosophila* line. Contigs other than the 5 major chromosome arms (X, 2L, 2R, 3L, 3R) and chromosome 4 are grouped into the 'other' category. Unmapped reads constituted 8% of the total reads, on average. (**Bottom**) The average depth of coverage is shown for each line, for each of the 5 major chromosome arms (X, 2L, 2R, 3L, 3R) and chromosome 4, with most lines having average depth > 12 for most chromosomes.

ddRAD Validation (12 lines)					
		ddRAD Genotype			
		REF	HET	ALT	*# Calls*
GATK	REF	**99.9%**	0.1%	0%	*333,312*
	HET	25%	**62%**	13%	*13,992*
	ALT	0.1%	0.2%	**99.7%**	*40,245*

100x Validation (ZW155)					
		100x Genotype			
		REF	HET	ALT	*# Calls*
GATK	REF	**99.7%**	0.3%	0%	*3.8M*
	HET	11%	**81%**	8%	*308,299*
	High	*5%*	***93%***	*2%*	*248,046*
	Low	*37%*	***30%***	*33%*	*60,253*
	ALT	0%	0.7%	**99.2%**	*572,859*

Figure S2 Summary of SNP Validation

SNPs were validated using two independent strategies. (**Top**) For 12 lines, genotypes called from ddRAD libraries covering ~1% of the genome matched the WGS SNP genotypes called by the GATK pipeline for >99% of homozygous genotypes. Heterozygous genotype calls from the GATK pipeline validated at a lower rate (62%) likely due to underrepresentation of both chromosome copies in the ddRAD libraries (Arnold *et al.* 2013; Davey *et al.* 2013; Gautier *et al.* 2013). (**Bottom**) For one line, ZW155, independent libraries were constructed and sequenced to 100x depth. Genotypes were called based on read depth per allele (see Methods) and compared to the genotypes called by the GATK pipeline from the ZW155 10x library. Again, calls matched for >99% of homozygous genotypes, but the validation rate was lower for heterozygous calls (81%). For the 100x dataset with full genome coverage, there were sufficient heterozygous calls to divide these into sites that fall in regions with *high* vs *low* heterozygous call frequency for this line. The validation rate was considerably higher in regions of high heterozygosity compared to regions of low heterozygosity, consistent with these being true regions of residual heterozygosity.

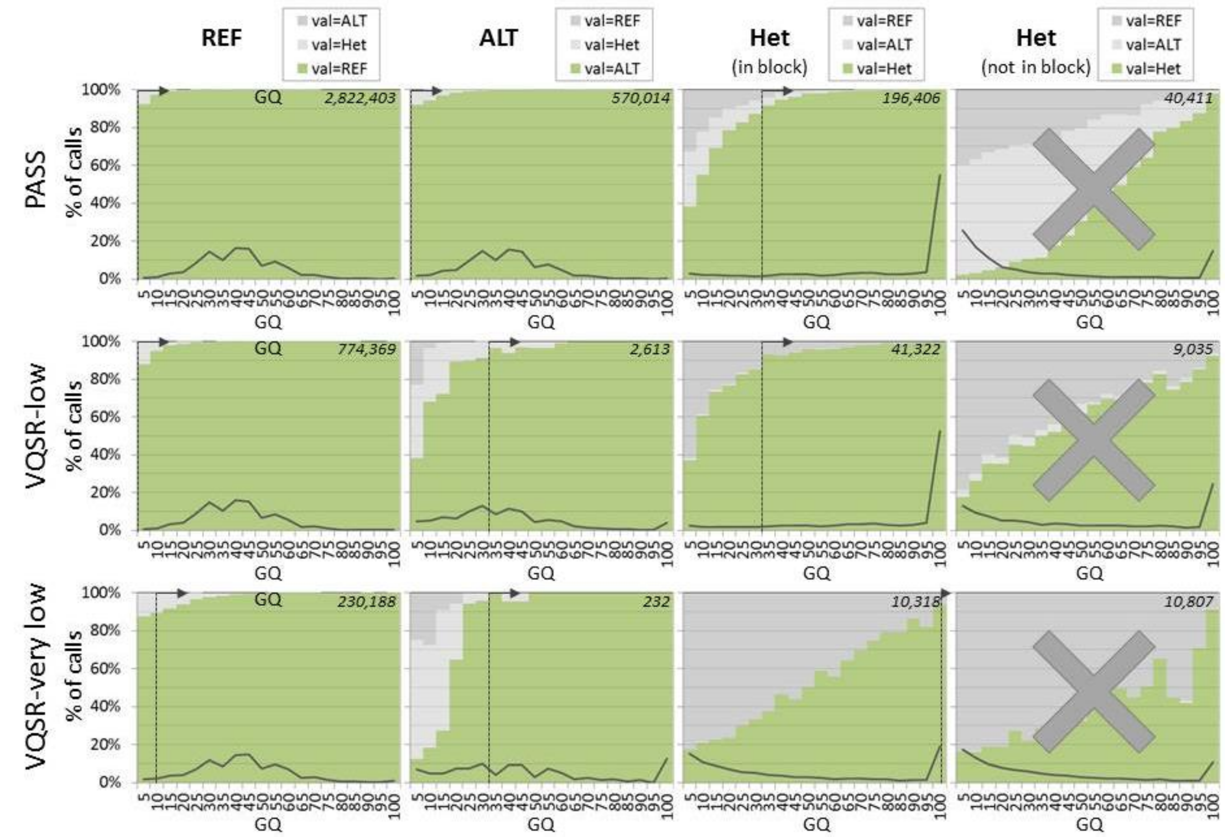

Figure S3 SNP Validation Rate Correlates with Genotype Quality Score

Validation rates were determined for different classes of GATK SNP calls, divided by Variant Quality Recalibration flag (rows: PASS, VQSR-low, VQSR-very low), SNP genotype (columns: REF, ALT, Het-high freq, Het-low freq), and Genotype Quality score (x-axes: GQ) for line ZW155 compared to the 100x validation dataset (val). Green bars indicate the genotypic agreement for each GQ bin; grey bars indicate discordant calls. The relative frequency of each GQ bin for each class of genotype call is shown as a dark grey line, with heterozygous calls having a bimodal GQ distribution. Vertical black lines and arrows indicate the GQ cutoff for each class of genotype call used to filter the SNP dataset. All heterozygous calls in regions of low heterozygous call frequency (assessed per line) were filtered out of the final SNP genotypes.

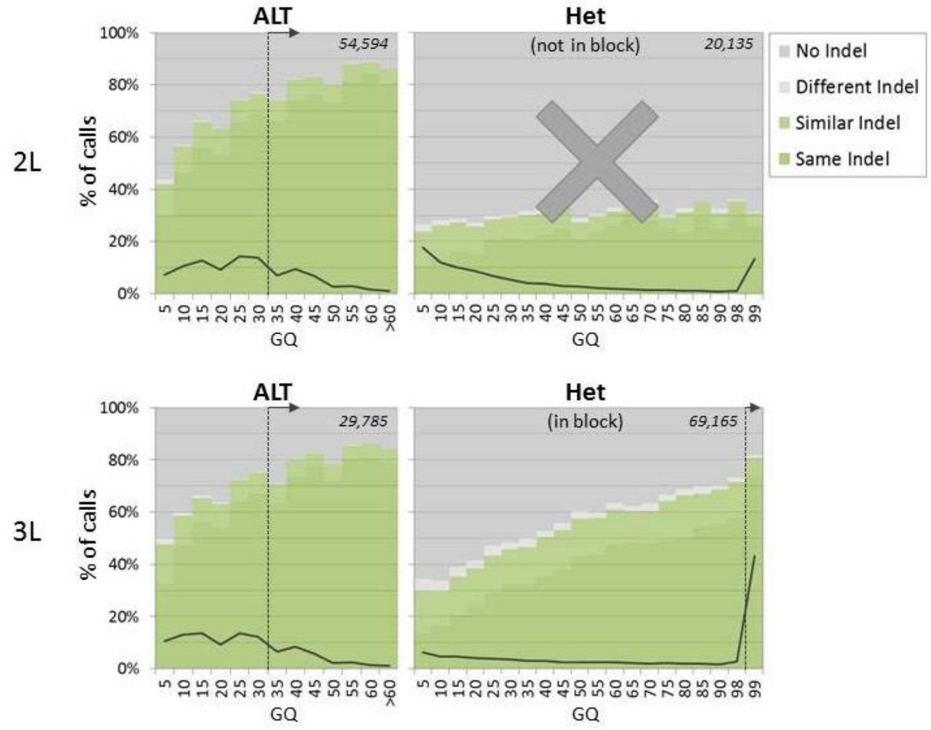

Figure S4 Small Indel Validation Rate Correlates with Genotype Quality Score
Validation rates were determined for different classes of GATK small indel calls, divided by chromosome (rows: 2L, 3L), small indel genotype (columns: ALT, Het), and Genotype Quality score (x-axes: GQ) for line ZW155 compared to the 100x validation dataset. Green bars indicate the genotypic agreement for each GQ bin; grey bars indicate discordant calls. The relative frequency of each GQ bin for each class of genotype call is shown as a dark grey line, with heterozygous calls having a bimodal GQ distribution. Vertical black lines and arrows indicate the GQ cutoff for each class of genotype call used to filter the small indel dataset. All heterozygous calls in regions of low heterozygous call frequency (assessed per line) were filtered out of the final small indel genotypes.

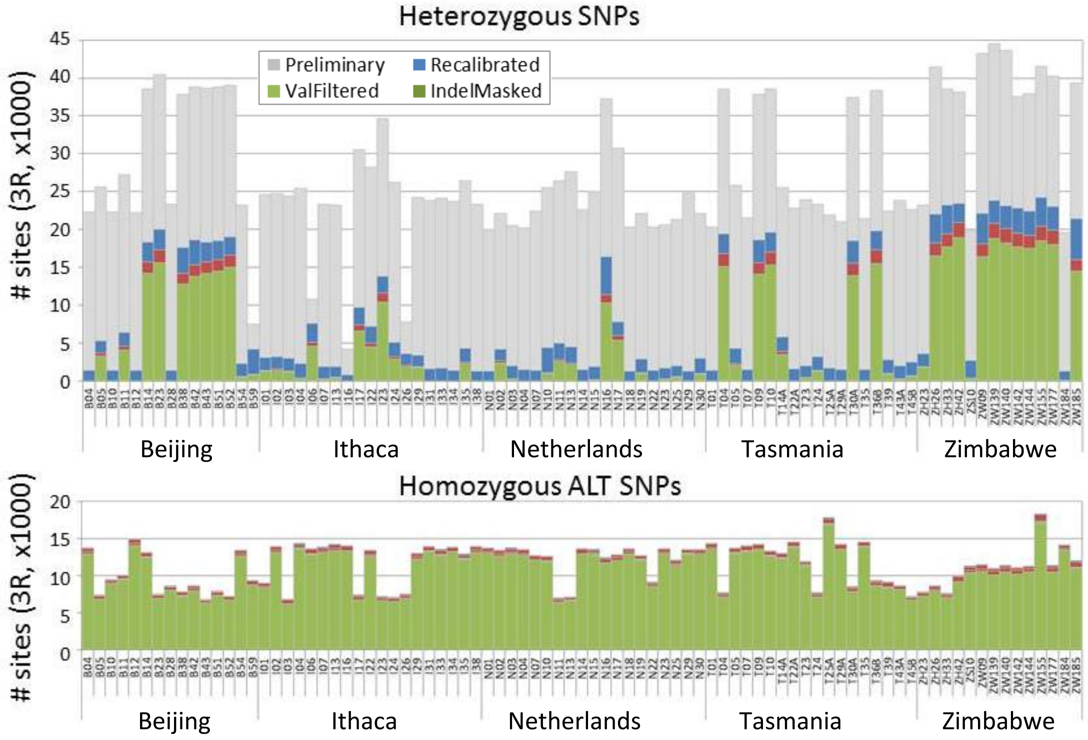

Figure S5 SNP Genotype Counts Per Line

The number of heterozygous (top) and homozygous ALT (bottom) SNP genotype per line is shown for a representative chromosome arm (3R), for each step in the SNP calling pipeline. The most significant change in SNP calls occurred at the Base Quality recalibration step for heterozygous calls, where the number of heterozygous calls dropped by 20,000 for most lines. After Base Quality recalibration, some lines (primarily from the Beijing and Zimbabwe populations) still had a high number of heterozygous calls. The SNP calling pipeline had much less effect on the homozygous ALT calls.

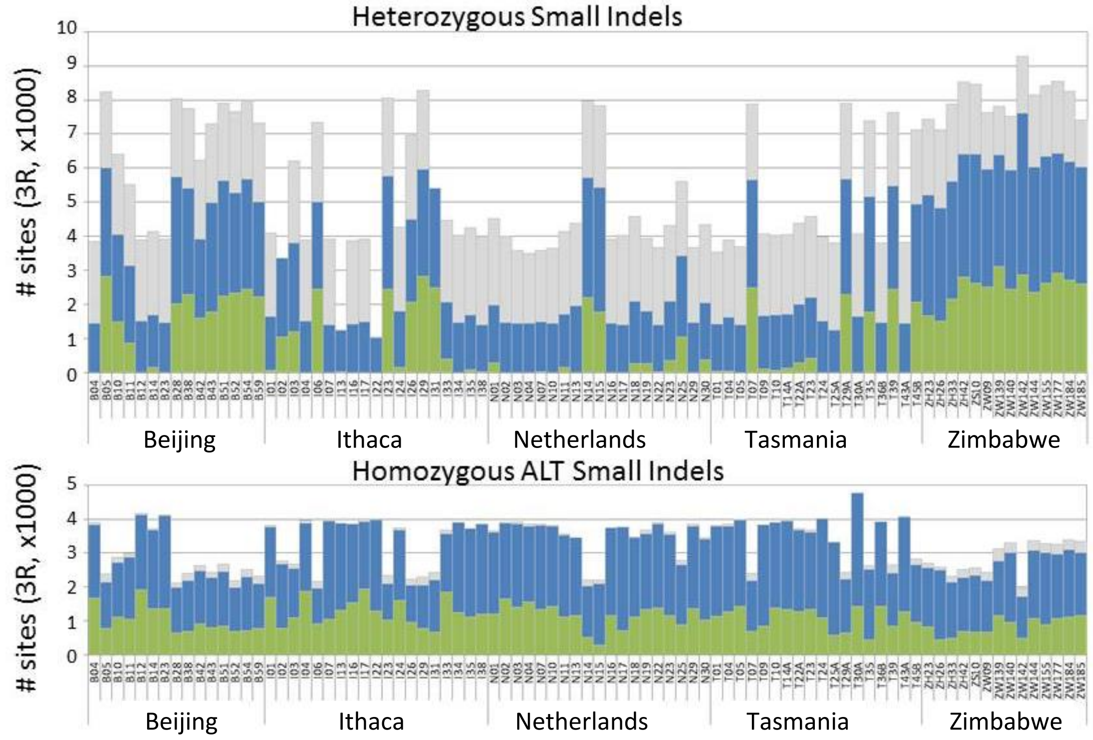

Figure S6 Small Indel Genotype Counts Per Line
The number of heterozygous (top) and homozygous ALT (bottom) small indel calls per line is shown for a representative chromosome arm (3R), for each step in the small indel calling pipeline. The two most significant change in small indel calls occurred at the Base Quality recalibration step for heterozygous calls, where the number of heterozygous calls dropped by 2,000 for most lines, and the Genotype Quality filtering step for all calls, which further reduced the number of small indel calls by 2,000 for most lines. After Base Quality recalibration, some lines (primarily from the Beijing and Zimbabwe populations) still had a high number of heterozygous calls, matching the same pattern seen for SNP calls (see Figure S5). Legend is the same as Figure S5.

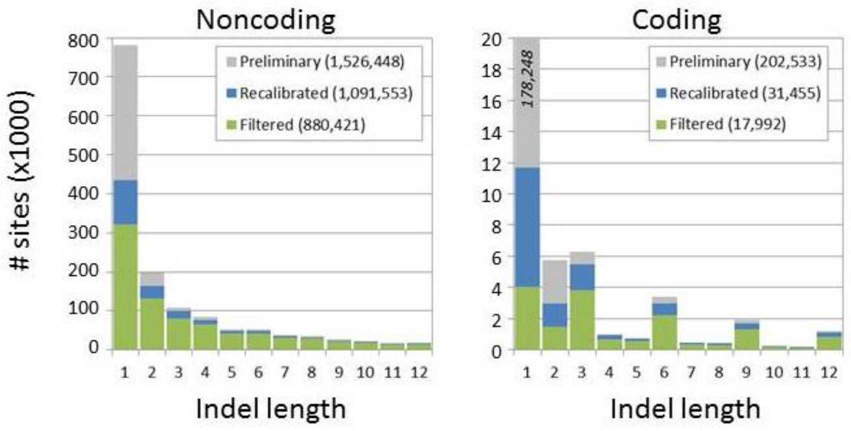

Figure S7   Small Indel Length Distribution
The number of 1-nt and 2-nt small indel variants dropped as calls were improved through the GATK pipeline, although the number of small indels in non-coding regions (left) were less affected than small indel calls within coding regions (right). The largest effect was a >90% reduction in the number of 1-nt small indels called within coding regions at the Base Quality recalibration step. Within coding regions, the selective constraint of maintaining the protein reading frame is evident in the 3nt periodicity in the small indel length distribution.

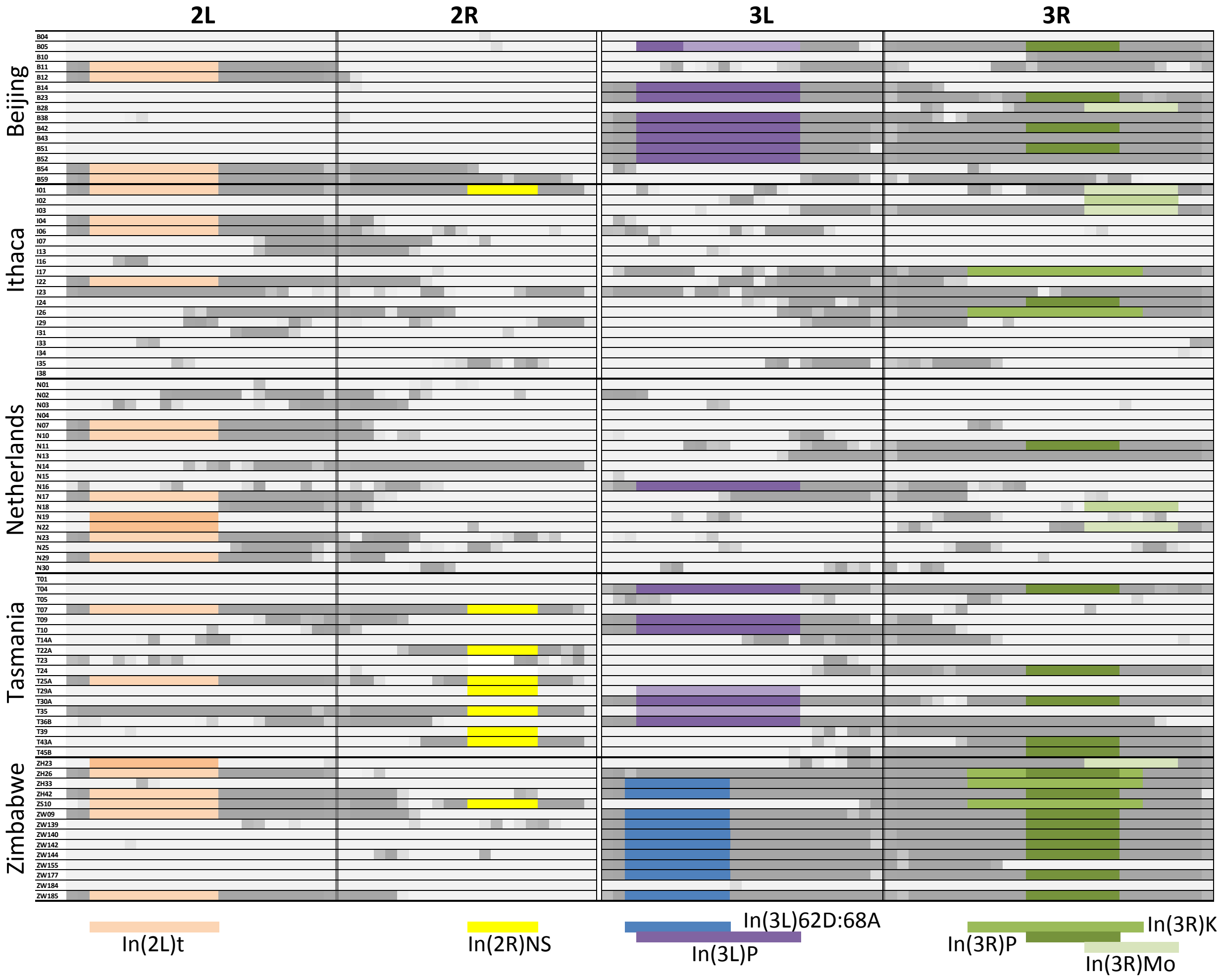

Figure S8 Large Known Inversions and Residual Heterozygosity
Regions with a high frequency of heterozygous genotype calls ('heterozygous blocks') are shown in grey for chromosomes 2 and 3 for each line. The lines from the Beijing and Zimbabwe populations have the highest frequency of heterozygous blocks, especially on chromosome 3. There is a clear correlation between the presence of residual heterozygosity and the presence of a heterozygous large inversion (colored bars). Overall, large inversions explain 57% of the observed heterozygous blocks.

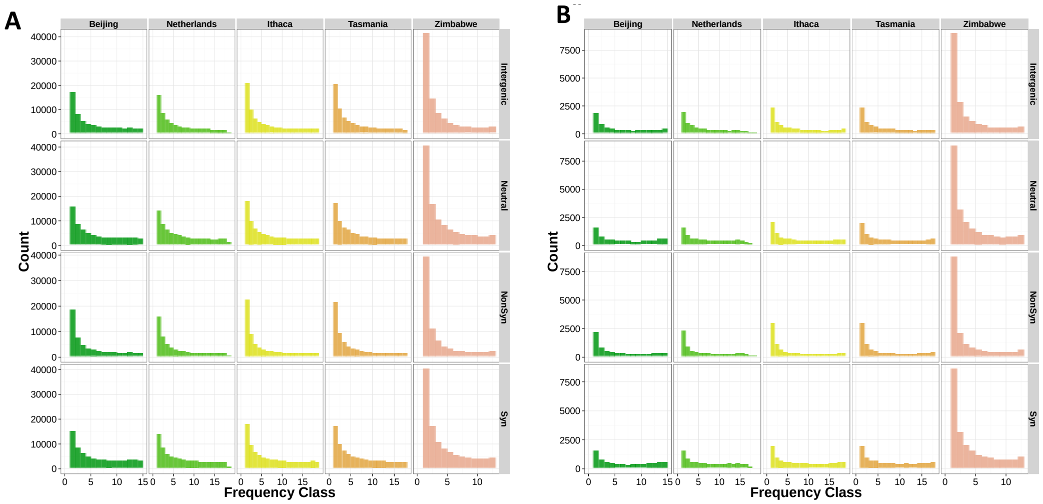

Figure S9   Population-Specific Site Frequency Spectra for Four Classes of SNPs

Unfolded SNP site frequency spectra (SFS) for four classes of SNPs separated by autosomes (**A**) and Chromosome X (**B**) for each population. Note that the X-axis varies between populations due to varying sample sizes. Most differences in SFS reside between the low frequency bins between the African sample and all non-African samples. There are is also a notable X-effect observed in the reduction of low frequency variants for the non-African samples. NonSyn = nonsynonymous sites; Syn = synonymous sites.

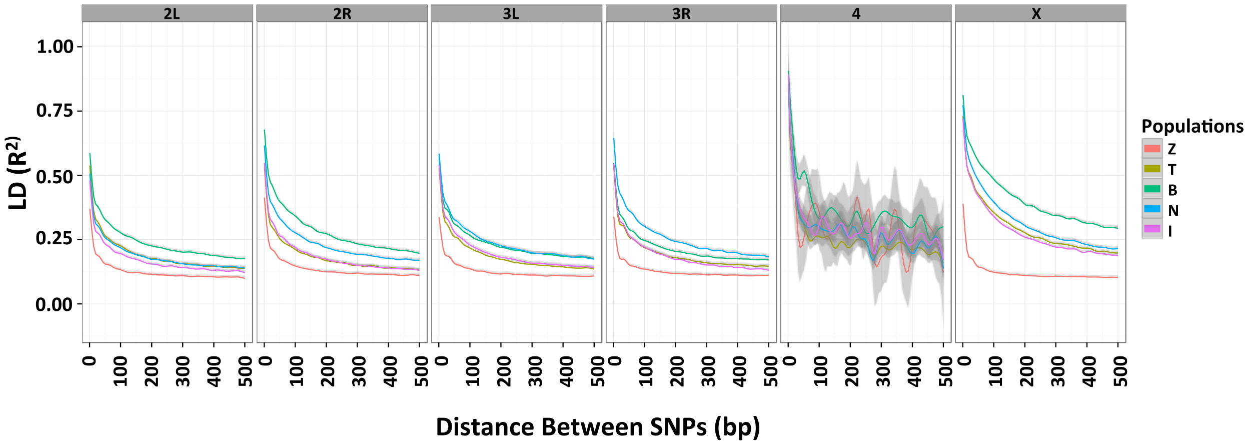

Figure S10 Decay of Linkage Disequilibrium Over Distance

Linkage disequilibrium as measured by R^2 divided by population and by chromosome. Most LD is quickly lost within the first 100-200 bp. The decay is most dramatic in the ancestral African population, particularly on chromosome X.
