# Supplementary material for: Global Diversity Lines–A Five-Continent Reference Panel of Sequenced Drosophila melanogaster Strains
Source: G3 (Bethesda). 2015 Feb 11;5(4):593–603. doi: 10.1534/g3.114.015883 (PMC4390575; doi:10.1534/g3.114.015883)
Supplement: Supporting Information [file supp_g3.114.015883_FileS1.pdf]

File S1

Expanded Materials and Methods

1. Global Diversity Lines

The Global Diversity Lines consist of 84+1 lines drawn from the initial set of 92 *D. melanogaster* lines inbred (sib-pair matings) for 12 generations from existing isofemale lines (Greenberg *et al.* 2010). The Global Diversity Lines were sampled from 5 populations: Beijing, China (15 lines; Begun and Aquadro 1995); Ithaca, NY USA (19 lines; Hill-Burns and Lazzaro 2004); Netherlands, Europe (18 lines; Bochdanovits and De Jong 2003); Tasmania, Australia (19 lines; Hoffman 2003); and Zimbabwe, Africa (14 lines; Ballard; Begun and Aquadro 1993). Seven of the original 92 lines were excluded from the final set because whole-genome sequencing indicated evidence of contamination (excessive heterozygosity or identity to another line). One additional line (ZW184) is included in the variant call set but excluded from all population-based analyses because it does not cluster with other Zimbabwe lines and may not have an African origin.

2. Genome Sequence Generation

Genomic DNA was extracted from ~50 non-virgin females per line with the Qiagen DNeasy Blood and Tissue kit. Each line was sequenced to a minimum 9x (average 12.5x) coverage on the Illumina HiSeq-2000 platform by BGI. In total, the whole-genome sequencing generated 1.7 B paired-end 100 nt reads (average 20.2 M per line). One line (ZW155) was independently sequenced to >100x depth from 3 additional WGS Illumina libraries made from the same genomic DNA stock by BGI.

3. Genome Alignments of Short Read Data

For SNP and small indel variant detection, raw sequence reads were mapped to the *D. melanogaster* reference (5.34) using BWA aln and BWA sampe (v0.5.9; (Li and Durbin 2009) with default parameters. Paired-end reads were reannotated with Samtools fixmate (v0.1.19; (Li *et al.* 2009) prior to merging bam files for all lines. To address alignment consistency between lines near small indels, the merged bam file was locally realigned using GATK v1.2 RealignerTargetCreator and IndelRealigner (McKenna *et al.* 2010; DePristo *et al.* 2011). The frequency of PCR duplicates in the paired-end sequences was so low (<<1%) that duplicate reads did not need to be removed.

Inversions were detected as part of a larger effort to identify structural variation in the Global Diversity Lines (M. Cardoso-Moreira, J. R. Arguello, D. Riccardi, S. Gotipatti, J. K. Grenier and A. G. Clark, unpublished). For this effort the raw sequence reads were mapped to the *D. melanogaster* reference (r5.34) using two different aligners, Novoalign (v2.07.11;

www.novocraft.com) and Mosaik (v1.1.0021; Lee *et al.* 2014). Default parameters were used with Novoalign with one exception: the penalty for gap extension was lowered (i.e. -x 6). Mosaik was run using Mosaik Jump (-hs 15), Mosaik Aligner (-hs 15 -mm 15 -mhp 100 -act 35 -bw 35) and Mosaik Sort (-rmm). See the section "Identification of Inversions" below for a description of the pipeline for calling inversion breakpoints.

4. SNP and Small Indel Calls

SNP and small indel calling was a multi-step process using GATK (v1.2; McKenna *et al.* 2010; DePristo *et al.* 2011) best practices. First, a preliminary run of the GATK UnifiedGenotyper generated 'preliminary' SNP calls that were subsequently processed and used as a 'truth set' for GATK Base Quality Score Recalibration (BQSR). Second, the GATK UnifiedGenotyper was run again on the BSQR merged bam file to call both SNPs and small indels (below). The resulting SNP VCF file was then used for GATK Variant Quality Score Recalibration (VQSR), using the filtered SNPs from the preliminary run as the training set.

4a. Base Recalibration

The 'truth set' used for the Base Recalibration was constructed with a set of high confidence SNP calls generated from the first pass GATK run across the full initial set of 92 lines. We designated SNP calls as 'high confidence' if they met the following criteria: 1) site quality score ≥ 30, 2) cumulative read depth greater than 30 and less than 190, and 3) If heterozygous in all variant lines, variant read frequency consistent with binomial expectation ($p \leq 0.05$). In addition, we examined the quality of the SNPs with respect to genomic annotations and the transition/transversion ratio (generated with SNPeff; Cingolani *et al.*)). These filters resulted in over 1 million 'high quality' SNPs with which to examine SNP-call covariates, and thus to recalibrate GATK's SNP calling model. The recalibrated model was then applied to the complete data set. We carried out the same procedure independently to identify 'high confidence' SNPs from the deeply sequenced (100x) ZW155 line. Although the total SNP set was smaller than for the 92 lines' set, the results in Base recalibration were minimal.

Examples of the GATK commands used for the Base Recalibration steps (using chr3L):

1) Run CountCovariates with standard covariats for each chromosome

```
java -Xmx16g -jar GenomeAnalysisTK.jar -nt 8 -R Dmel_r5.34.fasta -knownSites
Filtered_Truth_SNPs_3L.vcf -I Dmel_3L.realigned.bam -recalFile Dmel_filtered_cov_3L.csv -
T CountCovariates --standard_covs
```

2) Run TableRecalibration for each chromosome:

```
java -Xmx16g -jar GenomeAnalysisTK.jar -R Dmel_r5.34.fasta -I Dmel_3L.realigned.bam -T
TableRecalibration -recalFile Dmel_filtered_cov_3L.csv -o Dmel_filtered_recal_3L.bam
```

3) Re-genotyped with GATK's Unified Genotyper:

This was done with the same parameters as the initial 'first pass' genotyping but, due to memory requirements, were processed in chromosome segments and later concatenated:

```
java -Xmx16g -jar GenomeAnalysisTK.jar -R Dmel_r5.34.fasta -T UnifiedGenotyper -I
Dmel_filtered_recal_3L.bam -L 3L:20000001-23011544 -o Dmel_filtered_recal_3L.bam_5.vcf -
glm BOTH -stand_emit_conf 4 -stand_call_conf 10 -A DepthOfCoverage --output_mode
EMIT_ALL_SITES -dcov 100 -nt 8
```

4b. Variant Recalibration

SNP calls generated by GATK's Unified Genotyper were further refined using the variant quality score recalibration (VQSR). It assigns a well-calibrated probability to each variant call in a raw call set based on a truth set and uses this score to filter the raw calls. UG generated SNP calls which have more than 2 homozygous variant calls, a Phred-score greater than 20, and a mapping quality greater than 20 were used as a truth set.

1) Select all SNP calls from UG

```
java -Xmx4g -jar GenomeAnalysisTK.jar -R dmel-all-r5.34.fasta -T SelectVariants --variant
$VCF -o $VCF.snp_ALL.vcf -selectType SNP -restrictAllelesTo ALL
```

2) Generate a truth set

```
java -Xmx4g -jar GenomeAnalysisTK.jar -R dmel-all-chromosome-r5.34.fasta -T
SelectVariants --variant ${VCF}.snp_ALL.vcf -o ${VCF}.snp_ALL.filtered.vcf -select
"vc.getHomVarCount() > 2 && QUAL > 20.0 && MQ > 20.0"
```

3) Use VQSR to compute variant scores

```
java -Xmx4g -jar GenomeAnalysisTK.jar -T VariantRecalibrator -R dmel-all-chromosome-
r5.34.fasta -input $VCF.snp_ALL.vcf --qualThreshold 20.0 --percentBadVariants 0.03 --
maxGaussians 10 --mode SNP -
resource:GATK_FILTERED_${CHROM}_SNPS_ALL,known=false,training=true,truth=true,prior=10.0
${VCF}.snp_ALL.filtered.vcf -an QD -an HaplotypeScore -an MQRankSum -an ReadPosRankSum -
an FS -an MQ -an DP -an InbreedingCoeff -recalFile $VCF.variantRecal_after_baseRecal -
tranchesFile $VCF.tranches -rscriptFile $VCF.plots.R
```

4) Re-annotation of variant sites based on VQSR scores

```
java -Xmx4g -jar GenomeAnalysisTK.jar -T ApplyRecalibration -R dmel-all-chromosome-
r5.34.fasta -input $VCF.snp_ALL.vcf --ts_filter_level 99.0 -tranchesFile $VCF.tranches -
recalFile $VCF.variantRecal_after_baseRecal -o $VCF.snp_ALL.vcf.variantRecal.vcf
```

Sites with VQSLOD>99.9 were annotated as 'VQSLOD-verylow' and sites with VQSLOD>99 were annotated as 'VQSLOD-low'.

5. Defining Heterozygous Blocks

To investigate the 'blockiness' of heterozygous runs, the VCF file's genotypes at each variant site were converted into a binary sequence for each individual fly line (0 = homozygous, 1 = heterozygous). Within windows sliding along chromosomes, the observed number of consecutive heterozygous sites was compared with the expected number, where the state of each variant site was assumed to be independent outcomes of a multinomial distribution (the observed to expected ratio):

$$B = (Het^C / W) / ((Het^T / W) \times (Hom^T / W))$$

where Het^C is the number of consecutive heterozygous sites within the window of size *W*, Het^T is the total number of heterozygous sites within the window, and Hom^T is the total number of homozygous sites.

Two window sizes (20 SNPs or 2,000 SNPs) were tested to determine whether window size affected the ability to localize the ends of heterozygous blocks. The overlap between blocks of enriched heterozygosity was easily observable for the two window sizes, with the 20 SNP window producing more isolated 'spikes', as expected. Because there was good agreement between the two window sizes, we opted to use the 20 SNP window. Empirically, it was clear that windows having *B* values greater than 0.25 marked outlier regions. These small regions were then iteratively merged to form the larger heterozygous blocks. Adjacent regions were merged into a single block when separated by no more than 350 kb; the minimum size to retain a heterozygous block (after merging) was 200 kb. These regions were summarized in BED format and used in downstream analyses.

6. Variant Call Validation and Filtering

SNP calls were validated using two strategies. First, one line (ZW155) was independently sequenced to 100x depth by BGI (using 3 additional Illumina libraries distinct from the 10x coverage library). SNPs were called in this dataset by aligning paired-end 100 nt reads to the *D. melanogaster* reference genome (v5.34) with BWA (v0.5.9; (Li and Durbin 2009) using default parameters and then generating a base-count file at each variant position with coverage > 100 (Galaxy samtools_mpileup v0.0.1); homozygous genotypes were called at sites with >90% of reads having the same base, and homozygous genotypes were called at sites with 2 base identities each represented in >10% of reads.

Second, ddRAD libraries (Peterson *et al.* 2012) were generated for a subset of 12 lines (4 lines from Zimbabwe and 2 from each other source population). For each line, genomic DNA was digested with *EcoR*I and *Taq*$^{\alpha}$I and ligated to barcoded Illumina adaptors. The ddRAD libraries were separately size-selected on an agarose gel and amplified with TruSeq-compatible primers before pooling for Illumina sequencing (100 nt, single end reads) on a Hi-Seq2000. The ddRAD reads were processed to require and trim off the sample-specific barcode and an anchoring *EcoR*I site (AATTC) before mapping to the *D. melanogaster* reference genome (v5.34) with BWA aln and samse (v0.5.9; (Li and Durbin 2009) using default parameters. Similar to the 10x whole-genome coverage dataset, bam files were merged and realigned in indel intervals using GATK RealignerTargetCreator and IndelRealigner. Similar to the ZW155 100x validation dataset, SNP genotypes for each line were determined by generating a base-count file at each variant position with coverage > 100 using pileup (Galaxy); homozygous genotypes were called at sites with >90% of reads having the same base and homozygous genotypes were called at sites with 2 bases each represented in >10% of reads.

SNP calls for all lines in the original 10x dataset were filtered based on minimum validation rates for each genotype category and site type (PASS, VQSLOD-low, VQSLOD-verylow). The GQ at which each genotype category and site type exceeded 90% was used as a minimum cutoff for inclusion in the final SNP dataset:

Site Type	REF	ALT	HET (in block)
PASS	NA	NA	30
VQSLOD-low	NA	30	30
VQSLOD-verylow	7	30	99

In addition to SNP calls with GQ below the cutoff for each genotype category and site type above, all heterozygous SNP calls outside of heterozygous blocks were removed from the final SNP dataset. Finally, SNP calls within 5 nt of a small indel call (GQ>25) in the same line were also removed from the final dataset. Note that the GQ cutoff for small indel calls used here is more conservative than the final small indel call set (below) to minimize false-positive SNP calls adjacent to small indels.

Small indel calls were validated with the ZW155 100x dataset described above, using a similar strategy. Small indels in the 100x dataset were called independently with GATK UnifiedGenotyper (v1.2) and indel identity and genotype were compared to the ZW155 10x calls. A minimum validation rate of 75% was used to establish a minimum GQ for each genotype category, based on the validation rate of heterozygous indel calls with GQ≥7 for homozygous REF calls at indel sites, GQ≥30 for homozygous ALT indels and GQ=99 for heterozygous indels within heterozygous blocks. Small indel calls with GQ below these cutoffs were removed from the final dataset, as well as all heterozygous indel calls outside heterozygous blocks.

7. SNP Annotations Using Genomic Features

SNPs were annotated with respect to FlyBase genomic features using the SNPeff pipeline (v2.0.3; Cingolani *et al.*). VCF files generated from the above steps were provided to SNPeff, along with the reference genome (dm5.34) and FlyBase annotations using the following commands for each chromosome:

```
java -jar snpEff.jar eff -i vcf dm5.34 INPUT_2R.vcf -s 2R_SNPeff.html >
2R_SNPeff_summary.out
```

8. Identifying Regions of IBD

Germline (Gusev *et al.* 2009) was used to identify putative regions of genetic identity by decent (IBD) between all pairs of the 92 lines (non-defaulting settings: -min_m 1, -err_hom 1, -w_extend). Germline does not allow for missing data, so the 'full data set' of 92 lines was reduced to the subset of sites with all 184 alleles genotyped. This necessarily reduced the genetic variation in the samples, and thus represents conservative (overestimated) segments of putative IBD.

Our goals with the IBD analyses were twofold. First, we aimed to separate segments of candidate IBD that are more likely to have arisen from sampling closely related individuals from those segments of high identity that either have arisen by chance or as a result of overall low diversity. Second, measures of putative IBD are also useful for providing additional information regarding particular pairs of lines that stand out as problematic due to either unexpected shared identity (label switching) or unexpected amounts of putative IBD (contamination).

To identify the segments of IBD that would be retained within our masking files, we first ignored regions that fell within the lowly recombining regions of the pericentromeric and telomeric regions, as well as the fourth chromosome (Langley *et al.* 2012). We based the limits of the low recombination regions using the "*Drosophila melanogaster* Recombination Rate Calculator Version 2.3" (Fiston-Lavier *et al.* 2010), leaving the following chromosome segments for analyses:

Chrom	Region
X	2,222,391 – 20,054,556
2L	464,654 – 15,063,839
2R	9,551,429 – 20,635,011
3L	1,979,673 – 12,286,842
3R	12,949,344 – 25,978,664

The lowly recombining regions excluded from the above segments contained the greatest density of putative IBD segments, with many recurring both within and between populations. If IBD segments overlapped the low recombination boundaries, only segments having greater than 75% of their total length outside the low recombining regions were considered further.

Between- and within-population IBD segments that were identified by Germline (IBD^B and IBD^W, respectively) were separated, and the largest stretch of IBD^B was taken as an estimate for the extent of IBD observable by chance (3.8 Mb); IBD^W segments less than 3.8 Mb were not considered further. In total, 30 segments shared between individuals within the same populations were identified. Segments that retained IBD were masked in one randomly selected line for population genetic analyses.

9. Genome Callability

The following criteria were used to identify regions of poor mapping quality: a depth of coverage between 510 and 2040 inclusive (between one half and twice the average per-site depth including all lines) and that no more than 20% of covering reads have mapping quality zero. We found that about 88% of the reference genome was callable. Uncallable intervals are recorded the a bed file 'whole_genome_masked_intervals.bed'.

10. Identification of Inversions

We developed a pipeline aimed at identifying the breakpoints of inversions segregating in our dataset that consisted of two main steps. The first was a discovery step where bioinformatic tools (described below) were used to generate an initial set of candidate inversions. The second step consisted of an empirical evaluation of the initial set of candidate inversions by PCR and generation of breakpoint sequences using Sanger sequencing.

We created the initial set of candidate inversions by running two independent pipelines designed to detect inversions: Delly (i.e. invy; Rausch *et al.* 2012) and an in-house pipeline designed around BLAT (Kent 2002). The two pipelines identify inversions using complementary approaches: Delly identifies inversion calls based on paired-end information whereas the Blat-based pipeline identifies inversions based on split-read information. We ran Delly's invy module (v0.0.9) on each line using the alignments created by Novoalign (v2.07.11). Delly was run with default parameters, but we limited the detection of inversions to reads with a mapping quality score ≥ 20. The in-house pipeline based on BLAT is a simple extension of the pipeline previously developed by our group (Cardoso-Moreira *et al.* 2012). Briefly, the pipeline starts with the set of reads that Mosaik (v1.1.0021) failed to align. Those unaligned reads were re-aligned using BLAT (v3.4, -oneOff=1). Inversions were detected by selecting those reads that show a 'split signature', i.e. one read leads to two non-overlapping alignments, each on a different strand. We further required that the breakpoints detected using this split-read signature were located ≥ 30 bp away from the limits of the reads. To all calls made by Delly and the BLAT-based pipeline we applied the following filters: 1) for each genome inversion breakpoints had to be supported by at least 3 reads; 2) the inversion breakpoints could not overlap known transposable elements (annotated in Flybase; St Pierre *et al.* 2014) or identified by running RepeatMasker (Smit *et al.* 1996) on the 100 bp flanking each of the putative breakpoints); and 3) the candidate inversions had to be ≥ 1 Mb in size. In total, *each* pipeline identified a unique set of 109 candidate inversions with only 12 overlapping between the two sets. The lack of overlap was expected because the two pipelines use different signatures in the sequence data to identify inversions. Critically, all 8 *D. melanogaster* inversions with known breakpoints were independently identified by our approach.

From the set of 218 inversions predicted by the two pipelines, we designed primers to confirm both of the inversion breakpoints of a subset of 43. This subset includes 7 inversions with already mapped breakpoints and is heavily biased toward inversions predicted in multiple lines (as opposed to being private to one genome). Out of the 43 inversions tested we only obtained clear PCR bands for 17 (40%). However, it should be noted that we also only obtained clear PCR bands for 3 of the 7 inversions tested that already have mapped breakpoints. These results suggest that amplifying inversion breakpoints can be challenging, and that our approach is likely to have a high false negative rate in addition to a high false positive rate. In order to confirm the specificity of the PCR amplifications we attempted to sequence using Sanger sequencing 15 of the 17 breakpoints. We successfully sequenced 12, but of these only 6 proved to be true inversions. The remaining sequences suggested mis-

priming during PCR or the presence of structural variation at the breakpoints but not of inversions. After these efforts we generated breakpoint sequences for 5 of the 8 inversions with already known breakpoints and for two inversions with previously unknown molecular breakpoints (File S1). One of the inversions matches well the cytogenetic limits for In(3L)62D:68A described by Lemeunier and Aulard 1992 as a recurrent endemic. The other inversion with previously unknown molecular breakpoints, In(3R)13-72, does not match perfectly the cytogenetic limits of any inversion described by Lemeunier and Aulard 1992 but is located in the proximity to several inversions described. It should be noted that all 10 inversions with molecularly mapped breakpoints possess relatively simple breakpoint structures (Corbett-Detig *et al.* 2012). They were identified by our pipeline which, by design, attempts to exclude regions associated with transposable elements and other types of repeats, which are often associated with the genesis of inversions (Ranz *et al.* 2007). This suggests a potential significant ascertainment bias associated with the known inversion breakpoints.

11. Genotyping of Inversions

We inspected the genome sequences of the Global Diversity Lines for all 10 inversions with known molecular breakpoints. We used the breakpoint sequences that we generated (File S2) as part of our effort to identify inversions and those made available by Corbett-Detig and colleagues (Corbett-Detig *et al.* 2012). For In(3R)P we used the sequences deposited in Genbank by Matzkin and colleagues (Matzkin *et al.* 2005). Table 1 describes the origin of the breakpoint data used to genotype each of the 10 inversions. We genotyped these inversions *in silico* by mapping the raw genomic sequence reads of each genome against the reference genome sequence and all inversion breakpoints using BWA(Li and Durbin 2009). Inversions were called as being present in a given genome when reads from that genome spanned the inversion sequence breakpoints. We required that at least 2 reads spanned the inversion breakpoint with the latter not located within the last 15 bp of the reads. Inversions were called as homozygous when there were reads matching the inversion breakpoint but not the equivalent region in the reference genome. Inversions were called as heterozygous when there were reads matching both the inversion breakpoint and the equivalent region in the reference genome.

We genotyped individual flies for inversion breakpoints and for the reference chromosomal arrangement using a combination of novel primer designs and previously reported PCR assays for In(2L)t (Andolfatto *et al.* 1999) and In(2R)NS, In(3L)P, In(3R)K, and In(3R)Mo (all from Corbett-Detig *et al.* 2012). We also developed SNP genotyping assays for variant sites segregating within a line carrying an inversion that fall within a restriction site (the REF allele contains the restriction site and the PCR product is cut into two smaller bands).

Inversion	Variant	Primer 1	Primer 2	PCR Product Size ALT	REF
In(2L)t	Inversion breakpoint	GACTCTTTCTGCTTCGATCACTAAG	TATTTTGGTGGCCTGTTTCAG	250	None
	No inversion	TATTTTGGTGGCCTGTTTCAG	AAACACCACCAACGACATCC	none	240
	2L:2420388 T/C SNP	ACTAATCAGAGGCGCTTACATC	CTTGCTGCTATGTCACGCAC	298	HindIII: 184+114
In(2R)NS	Inversion breakpoint	TGGCCTGCTTCTGGTCCTCT	GGCGAGCCATCATTGTTATC	249	None
	No inversion	TGGCCTGCTTCTGGTCCTCT	AGAAGCACGCTGAGGAAATG	none	338
	2R:13048623 C/A SNP	CTGTGATACCCTACGCCGAC	AGCAAGTACGAGTGGAGAAGAC	218	HindIII: 140+77
In(3L) 62D:68A	Inversion breakpoint 1	AGAAGCTCTTTCGCAAATGG	GCATCGCAAGATTGTTTCC	800	None
	Inversion breakpoint 2	GCTGCAATTGTACATCGTTCC	TCACTTGGATTGCTTTGCTTG	750	None
	No inversion	GCATCGCAAGATTGTTTCC	TCACTTGGATTGCTTTGCTTG	none	500
	3L:1,325,296 A/G SNP	CATGGCCAGGTAGAAGAAGC	TGCAATGGATTTGTGACTGG	159	XbaI: 39+120
	3L:5,798,786 A/T SNP	CACGGGTATTCCACTCAAGG	GGATTCACCTGATGATACAACG	292	XbaI: 93+199
In(3L)P	Inversion breakpoint	CGGGAATGGTAGCTAGACCA	GTGAGCTCAACCCATTCGGT	306	None
	No inversion	GCTGATTCGCTTTGTCTTCG	GCCTGAAGTGCTGAAAGTGG	none	272
	3L:9034742 G/C SNP	AATGGATATGCGGATGCAG	TTCGATCAACACCCATAGACC	110	XbaI: 78+32
In(3R)K	Inversion breakpoint	TCTGACCCACTCTCCACTTG	CGAAAACCACAAGTACGCCTT	244	None
	Reference	GGGCATACACGAAAGAAGGTC	AGCCCGTGTGGTAATCGTAG	none	236
	3R:21878478 G/A SNP	TGATTAGGCGTTGAAGCCCTG	AGGGTGTGCGCGATTCTAAG	299	HindIII: 224+74
In(3R)Mo	Inversion breakpoint 1	TTGAAAGGTGATCCCAGATATAAG	TCGCCACAGTGTATGACTGC	278	None
	Inversion breakpoint 2	ACCTCACTGCGGATGAAGAG	TCCATGGCAATACCTTCACA	400	None
	No inversion	TCGCCACAGTGTATGACTGC	TCCATGGCAATACCTTCACA	none	308
	3R:17827239 G/T SNP	TTGCAGCAACAACAAATGCG	TGTTCTTGCCGTGTGCTG	250	BamHI: 161+89

Single-fly genomic DNA was isolated for PCR genotypig according to Gloor *et al* (Gloor *et al.* 1993), using LongLife Proteinase K (GBiosciences). PCR reactions consisted of 2 µL single-fly gDNA, 500 nM each PCR primer, 20 mM Tris pH 8, 50 mM KCl, 1.5mM $MgCl_2$, 0.2mM dNTPs, 5% DMSO, and 0.5ul *Taq* polymerase in 50 µL total volume, with cycling conditions 95 °C/15 min; 95 °C/15 sec – 53 °C/10 sec -72 °C/90 sec] x 40 cycles; 72 °C/5 min. For SNP assays, the PCR product was digested by adding the appropriate restriction enzyme in 1x PCR buffer and incubating at 37 °C for 3 hours; fragment sizes were resolved on 1.5% Agarose gels (1x TAE).

12. Genetic Tests of Chromosome Homozygosity

Individual males were selected from fly stocks and crossed to virgins from a double-balancer strain (Bloomington stock #2475: *w*[*]; T(2;3)*ap*[*Xa*], *ap*[*Xa*]/*CyO*; *TM3*, *Sb*[1]). Multiple sib-pair F1 crosses (*CyO/*+; *TM3/*+) were set up from each parental cross to sample each possible F1 x F1 genotype combination. F1 adults were individually genotyped (see above) after the F2 generation was initiated to determine the identity of the chromosome inherited from the founder male. F2 progeny were scored for dominant markers on both balancer chromosomes (minimum 20 F2 progeny required per cross).

13. Diversity Estimates

Diversity estimates and summary statistics were computed using the VariScan package (v2.0.3; (Vilella *et al.* 2005; Hutter *et al.* 2006). For input to VariScan, VCF files were first filtered using the IBD and genome callability masking files and

then converted to HapMap format. Conversion was accomplished by using VCFtools (v0.1.11; (Danecek *et al.* 2011) to convert the VCF files to TPED format, which was then converted to HapMap format using the Perl script "convert_tped_to_hapamp.pl" available on GitHub (https://gist.github.com/pamag/2069211).

To generate the data for Figure 8A, summaries were calculated for windows sized by the number of polymorphic sites (WindowType = 2), with the size equal to 10,000 for autosomes and chrX, and 500 for chr4 (WidthSW = 10000; WidthSW = 500). The stride size was 5,000 sites for autosomes and chrX, and 250 for chr4 (JumpSW = 5000; JumpSW = 250). Additionally, summary statistics were computed using the total number of mutations (UseMuts = 1), and the minimum number sequences at each site were required to be at least 14 or 15 (NumNuc = 14; NumNuc = 15).

14. F_{ST} and Migration:

F_{ST} between each pair of populations was calculated using the unbiased approach of Weir and Clark (1984), which allows for unequal sampling between populations. To generate Figure 4, *m*, the per generation migration rate, was approximated by using the equilibrium from the Wright Island model , $F_{ST} = 1 / (4N_e m + 1)$, and solving for *m*, using 10^6 as an estimate of the effective population size, N_e.

15. Identifying Small Intronic and 4-fold Degenerate SNPs

For several population genetic analyses (i.e examining population structure or demographic effects), obtaining estimates from sequences that behave the most neutrally (most free of selective pressures) can be informative. In the *D. melanogaster* genomes the two classes of sites that have been shown to behave most neutrally are SNP within short (<=65 bp, bases 8-30) introns (SI) and 4-fold degenerate sites (4D) (Parsch *et al.* 2010). To extract SI sites that are polymorphic within the dataset, all SNPs that were annotated as intronic based on the SNPeff output (above) were outputted in "bed" format file. A second bed file that was composed of SI intronic start/end coordinates based on the genic annotation of reference *D. melanogaster* genome (r5) was generated. Introns that were not 'dedicated' (found to be coding in >=1 isoform) were excluded. These two bed files were intersected using BEDtools (v2.17.0;(Quinlan and Hall 2010), generating a list of SNPs in our dataset that fall within SI. Similarly, a bed file was generated for all 4-fold degenerate positions (as annotated by SNPeff) that are polymorphic within the dataset. Redundant positions that resulted from isoforms were removed.

16. Whole Genome Alignment and Species Divergences

For accurate computation of *k*, a measure of divergence per site, and the Polymorphism / Divergence ratio, we created a custom-built multiple genome alignment using a recent, revised *D. simulans* genome assembly superior to the earlier mosaic assembly of this species (Hu *et al.* 2013). We created a whole-genome alignment of the 5 *melanogaster*-subgroup species using publically accessible genome assemblies for *D. melanogaster* (dm3), *D. simulans* (droSim2), *D. sechellia* (droSec1), *D. erecta* (droEre2) and *D. yakuba* (droYak2). Besides the revised *D. simulans* genome assembly, all other genome assemblies were downloaded from the UCSC genome browser (http://genome.ucsc.edu/). Pairwise alignments of *D. melanogaster* to the other assemblies were created using LASTZ (v1.03.34; Harris 2007). These pairwise alignments were further refined into single-coverage alignments using the chaining and netting utilities as prescribed from the UCSC genome browser. Subsequently, these were later merged into a single whole-genome multiple alignment utilizing the *roast* program of the MULTIZ software package ; (Blanchette *et al.* 2004; updated Jan 21, 2009). This 5-way multiple-alignment file (MAF) is available upon request and the alignment is viewable at http://genome-mirror.cshl.edu/. To access this alignment, users should navigate to the *D. melanogaster* genome assembly (dm3) and enable the "Conservation 5way" track.

We computed divergence per site (*k*), the average number of nucleotide substitutions per site between the *D. melanogaster* and *D. simulans,* by first counting the number of nucleotide differences between the two species conditioned on the multiple alignment. Estimates of *k* were computed using the same window sizes as the measure of nucleotide diversity. In each window, sites without an aligned base in the *D. simulans* assembly were filtered. The ratio of divergence per window length (*D*) were then corrected for multiple substitutions using the Jukes-Cantor correction (Jukes and Cantor 1969), $k=-(3/4)\ln(1 - (4/3)D)$.

References

Andolfatto, P., J. D. Wall, and M. Kreitman, 1999 Unusual haplotype structure at the proximal breakpoint of In(2L)t in a natural population of Drosophila melanogaster. Genetics 153: 1297–311.

Arnold, B., R. B. Corbett-Detig, D. Hartl, and K. Bomblies, 2013 RADseq underestimates diversity and introduces genealogical biases due to nonrandom haplotype sampling. Mol. Ecol. 22: 3179–90.

Ballard, W. Isofemale D. melanogaster lines (’ZH') collected near Victoria Falls, Tasmania.

Begun, D. J., and C. F. Aquadro, 1993 African and North American populations of Drosophila melanogaster are very different at the DNA level. Nature 365: 548–50.

Begun, D. J., and C. F. Aquadro, 1995 Molecular Variation at the vermilion Locus in Geographically Diverse Populations of Drosophila melanogaster and D. simulans. Genetics 140: 1019–1032.

Blanchette, M., W. J. Kent, C. Riemer, L. Elnitski, A. F. A. Smit *et al.*, 2004 Aligning multiple genomic sequences with the threaded blockset aligner. Genome Res. 14: 708–15.

Bochdanovits, Z., and G. De Jong, 2003 Temperature dependent larval resource allocation shaping adult body size in Drosophila melanogaster. J. Evol. Biol. 16: 1159–67.

Cardoso-Moreira, M., J. R. Arguello, and A. G. Clark, 2012 Mutation spectrum of Drosophila CNVs revealed by breakpoint sequencing. Genome Biol. 13: R119.

Cingolani, P., A. Platts, L. L. Wang, M. Coon, T. Nguyen *et al.*, 2012 A program for annotating and predicting the effects of single nucleotide polymorphisms, SnpEff: SNPs in the genome of Drosophila melanogaster strain w1118; iso-2; iso-3. Fly (Austin). 6: 80–92.

Corbett-Detig, R. B., C. Cardeno, and C. H. Langley, 2012 Sequence-based detection and breakpoint assembly of polymorphic inversions. Genetics 192: 131–7.

Danecek, P., A. Auton, G. Abecasis, C. A. Albers, E. Banks *et al.*, 2011 The variant call format and VCFtools. Bioinformatics 27: 2156–8.

Davey, J. W., T. Cezard, P. Fuentes-Utrilla, C. Eland, K. Gharbi *et al.*, 2013 Special features of RAD Sequencing data: implications for genotyping. Mol. Ecol. 22: 3151–64.

DePristo, M. A., E. Banks, R. Poplin, K. V Garimella, J. R. Maguire *et al.*, 2011 A framework for variation discovery and genotyping using next-generation DNA sequencing data. Nat. Genet. 43: 491–8.

Fiston-Lavier, A.-S., N. D. Singh, M. Lipatov, and D. A. Petrov, 2010 Drosophila melanogaster recombination rate calculator. Gene 463: 18–20.

Gautier, M., J. Foucaud, K. Gharbi, T. Cézard, M. Galan *et al.*, 2013 Estimation of population allele frequencies from next-generation sequencing data: pool-versus individual-based genotyping. Mol. Ecol. 22: 3766–79.

Gloor, G. B., C. R. Preston, D. M. Johnson-Schlitz, N. A. Nassif, R. W. Phillis *et al.*, 1993 Type I repressors of P element mobility. Genetics 135: 81–95.

Greenberg, A. J., S. R. Hackett, L. G. Harshman, and A. G. Clark, 2010 A hierarchical Bayesian model for a novel sparse partial diallel crossing design. Genetics 185: 361–73.

Gusev, A., J. K. Lowe, M. Stoffel, M. J. Daly, D. Altshuler *et al.*, 2009 Whole population, genome-wide mapping of hidden relatedness. Genome Res. 19: 318–26.

Harris, R. S., 2007 Improved pairwise alignment of genomic DNA: Pennsylvania State University.

Hill-Burns, E., and B. Lazzaro, 2004 Isofemale D. melanogaster lines collected in Ithaca, NY.

Hoffman, A., 2003 Isofemale D. melanogaster lines from Tasmania, Australia.

Hu, T. T., M. B. Eisen, K. R. Thornton, and P. Andolfatto, 2013 A second-generation assembly of the Drosophila simulans genome provides new insights into patterns of lineage-specific divergence. Genome Res. 23: 89–98.

Hutter, S., A. J. Vilella, and J. Rozas, 2006 Genome-wide DNA polymorphism analyses using VariScan. BMC Bioinformatics 7: 409.

Jukes, T., and C. Cantor, 1969 Evolution of protein molecules, pp. 21–132 in *Mammalian Protein Metabolism*, edited by H. Munro.

Kent, W. J., 2002 BLAT--the BLAST-like alignment tool. Genome Res. 12: 656–64.

Langley, C. H., K. Stevens, C. Cardeno, Y. C. G. Lee, D. R. Schrider *et al.*, 2012 Genomic variation in natural populations of Drosophila melanogaster. Genetics 192: 533–98.

Lee, W.-P., M. P. Stromberg, A. Ward, C. Stewart, E. P. Garrison *et al.*, 2014 MOSAIK: a hash-based algorithm for accurate next-generation sequencing short-read mapping. PLoS One 9: e90581.

Lemeunier, F., and S. Aulard, 1992 Inversion Polymorphism in Drosophila melanogaster, pp. 339–406 in *Drosophila Inversion Polymorphism*, edited by C. B. Krimbas and J. R. Powell. CRC Press.

Li, H., and R. Durbin, 2009 Fast and accurate short read alignment with Burrows-Wheeler transform. Bioinformatics 25: 1754–60.

Li, H., B. Handsaker, A. Wysoker, T. Fennell, J. Ruan *et al.*, 2009 The Sequence Alignment/Map format and SAMtools. Bioinformatics 25: 2078–9.

Matzkin, L. M., T. J. S. Merritt, C.-T. Zhu, and W. F. Eanes, 2005 The structure and population genetics of the breakpoints associated with the cosmopolitan chromosomal inversion In(3R)Payne in Drosophila melanogaster. Genetics 170: 1143–52.

McKenna, A., M. Hanna, E. Banks, A. Sivachenko, K. Cibulskis *et al.*, 2010 The Genome Analysis Toolkit: a MapReduce framework for analyzing next-generation DNA sequencing data. Genome Res. 20: 1297–303.

Parsch, J., S. Novozhilov, S. S. Saminadin-Peter, K. M. Wong, and P. Andolfatto, 2010 On the utility of short intron sequences as a reference for the detection of positive and negative selection in Drosophila. Mol. Biol. Evol. 27: 1226–34.

Peterson, B. K., J. N. Weber, E. H. Kay, H. S. Fisher, and H. E. Hoekstra, 2012 Double digest RADseq: an inexpensive method for de novo SNP discovery and genotyping in model and non-model species. PLoS One 7: e37135.

Quinlan, A. R., and I. M. Hall, 2010 BEDTools: a flexible suite of utilities for comparing genomic features. Bioinformatics 26: 841–2.

Ranz, J. M., D. Maurin, Y. S. Chan, M. von Grotthuss, L. W. Hillier *et al.*, 2007 Principles of genome evolution in the Drosophila melanogaster species group. PLoS Biol. 5: e152.

Rausch, T., T. Zichner, A. Schlattl, A. M. Stütz, V. Benes *et al.*, 2012 DELLY: structural variant discovery by integrated paired-end and split-read analysis. Bioinformatics 28: i333–i339.

Sezgin, E., D. D. Duvernell, L. M. Matzkin, Y. Duan, C.-T. Zhu *et al.*, 2004 Single-locus latitudinal clines and their relationship to temperate adaptation in metabolic genes and derived alleles in Drosophila melanogaster. Genetics 168: 923–31.

Smit, A., R. Hubley, and P. Green, 1996 RepeatMasker Open-3.0.

St Pierre, S. E., L. Ponting, R. Stefancsik, and P. McQuilton, 2014 FlyBase 102--advanced approaches to interrogating FlyBase. Nucleic Acids Res. 42: D780–8.

Vilella, A. J., A. Blanco-Garcia, S. Hutter, and J. Rozas, 2005 VariScan: Analysis of evolutionary patterns from large-scale DNA sequence polymorphism data. Bioinformatics 21: 2791–3.

Weir, B., and C. C. Clark, 1984 Estimating F-Statistics for the Analysis of Population Structure. Evolution (N. Y). 38: 1358–1370.
