# Supplementary material for: Global Diversity Lines–A Five-Continent Reference Panel of Sequenced Drosophila melanogaster Strains
Source: G3 (Bethesda). 2015 Feb 11;5(4):593–603. doi: 10.1534/g3.114.015883 (PMC4390575; doi:10.1534/g3.114.015883)
Supplement: Supporting Information [file supp_g3.114.015883_FileS2.pdf]

File S2

Inversion breakpoint sequences

```
>In(3R)13-72_proximal
GTCCTCGCTCTCTGTCTTGGTTTCTGTCTTGGCATTCTTGCTGGACGCCCCTCCGCCATTCTCCTCCAGC
CACTTTTCAAAGACTCCAATGGCCGCATTGATGTTCTTCAGCTTCTCCTCCGCCTTGGTCACTAGAATGG
TAGGTTTAGTGCTGGATTCAACGCATCCCTTGGGCACAAGCACTCACTTGTCAGGACTCTTTTGGCCCGG
CCAAGCAAACCGCGCACCGTCAGCTTGCGATACTGCATGTCGTGACTCTCCAGCAGCTTCAACGTCTCTA
ACGCCTTCTCCATGATGACACCACGGAGATGACCTTCAGCTTGAACAGTTTGGATCTGGATAATATTCGT
CCGCCGTCAGGAATGGAATCGCTGAACAGCTGCTACCAGGATCACTCCCAGCCTAGTTCTCTGCGCCAGC
CGATGCCCAGCAAGAGTCCACGCTTCGCGCGAAAAATGTTTCCAGCCAATTTGGTAGCCAGACGAGCACT
GGGTCACTTAGCTGGCAGTGCGGAGAGCGTGAACTCCAGCTGCAACCTGCTGGACAACATCAAGCCTCCG
TCACTGATGGATGAGCTGCTGGACTCGATGATCAGTGTGGACAGCATTCAGTCTGAGGTGGCCGAGGGCG
AGCAGGACTGCAGCATGGCCACCACCATTTCCGTGTCCAACTATGAGACGGCTGCTTGCGATGATCAGAC
GATGACCGTGCTCCAAAGTTGTTTCGACGAGGATGAGGATGCCACAATGAATGACTACAGCTCGGCCGAG
TCCACACCCAAGCACGGATCTACTCCATCACCCAATCGTCGTTCCCTCACACCGAAAGAC
```

```
>In(3R)13-72_distal
CAACTAGCCCATCGTTCACCGCTAAGCCCAAAGCTTTTTGAGTTCCATGTTAATAAACAACACAAACAAA
TTATAACAATTTTCGTATTGATTGGGGAAAATATATCATAATAAATATGATTGCAAATATAACGACAGCA
ATGTCATATTACATGCTTTAATATGCAGAGCGGCGTTTAATTAGGTAATTAATACTTAAGATCGTTTATT
TCCTCTCAATGTTATCAGTTTCTCGTGGCGCTGACGACATGGCTAAGTATCGATTATTTTCTCGTGGGTT
TTGTTTTGCTCCGGTGCTACGACAACAAACCGGTTTCCAAACATAGAGTCACCCAGTTATTTTGTGGTGC
TTATCTAATTGGCCACGCTCAGCTCTGCTCAGTGCCTCTCAGTGGTGCTCATTTTGCGTCTTCCTCCCAT
GATTAAGTGCCATATGCCGTCGAGTTGGAGACGGCTACGCAAAATCAGTCGTATTCTAGCGCTGACAGGA
AGCAGACAGATACTCACCCAAGTATTAGCAACCAAAGGAGCAGCAATGGCAGAGGGAGATTCGAAATTTG
CCGGGCTGGAGTAGATTTTCCGCCAGAGCTCGCGCTTCGAACGCAATCATGGTCGAGTTTGTCATAATGG
AGGCACTCGTTCCATTAAATAATAGTCTTTGCGGCTCCAATCTA
```

```
>In(3L)62D:68A_proximal
AGAAGCTCTTTCGCAAATGGATTTTCGGGTTCGTCATTTAACTACAATATTTTGTGGGCCTTTCAAGGTT
CGTCTATTGTCCTGAAGGAAACTACGCAGAAGGCTTAAGTTTATTGTTTATTATGTGCGTTTTATGGCAC
GTTGTGCTTGTTTGCTTTTCCATTGCGTATTTAGAAGTTTATTAATCATACCTTTTACTACGACGACTTT
ATGAACATAAATTCTTGAAATTTGGAATTCCGATGAAACGAAAACAATACCTTTTTGTGAAGTTCAGGTA
AAGTAGAAAGAGTTCAATATCACAAAGAGCTGAACGATGACAGAAAGAGGTTTAAAGGATTTAGGCTTCA
GATACTTTTTGTTCATTTTTCAAGCTGAGGCATATAACCTGCTACTTTAAGTATTTTAATGGTTAGGCGC
CCTACCGACCTAATCTGTTCTTAATTTGTAAATTTTTGATCACTCGCCAACTCAGTCGCTTGCCATTAAA
ATCCCATTAAGTTTGAAGCTCAGACATATGAAAATTGAAAATTAAGTATACTGAAAGCTTTTTCATGCGC
AATACTAGTATACTAGACAATCAGCACATACATATGTACTTTAAAACATAAATATGTAAAGCATACTTAT
TCCACTCAAACAAAACTTTAATCAAATTAATCAAATTGCGCGCAAAAGAAGCTGTGAAAAACAAGTAAAA
AGTAAATGAGAACGCTAGAAAAAGTTCAAATTTTCAAAAGTGCGGCCGTTTTTTGGGCGTGGCAAGAAAG
AATCCGGAAACAATCTTGCGATGC
```

```
>In(3L)62D:68A_distal
GCTGCAATTGTACATCGTTCCATGTAATTTACTATAAATAACATTATATCTAATCGGACCGGGATTCAGG
CGCCTCCGAACCGTATTAGTTGCATTCATTGGCTGCAATTTGCATAATGATGCTGACTTTCGGGGGCACA
GACATCAAGCCGGGCTCAAGTCCCAATTATGTCTGCTACATTTTGTTTAATGCCCTCAATTTACCAGCTC
AAAGTTATCTCTGCAGCATGGAAAACTCCCCAGGCAAAGTCGAGTGAATTGCAATCCGTCGGTTGCCCAG
GATATTCGGCCAATTAGTTTCCTTGTGTGGGCTGCTTCTGCAGTAGACCCCCCCCCCCTTCCTCCTCGC
ATTCGTCACACGGCACGTATGCGCAAGGTAAAACAATTTACGCTCAACTGGTTTCTAGCCCCATGCAACA
```

GGTTATTCAAAAATAATATACAAATCTATGCATAAAGCTCAAGTATTCTTTTGTTAAAATCCCTTTTTAA
CGAATTATTTTACCTACGGGTTATAGGTTTAAAAAACAAAAGTATTTGTGGGGGGGTGTAATATGACCTG
GGTCAAAACATAACAGCGAAACACTCCAAGCTTACAAAGCATTTATACAAATTTTTTTTTAGCAAAATTT
AACAATTAGCTTCAATTTCTGTTGGAATTTTTAAGTTTTAACCCATATTCTTAAAACCAAACGATCAGCT
AAATCTCTAAACTTGCAAGCAAAGCAATCCAAGTGA

>In(2R)NS
GCCACTTCGATTCTGCAAAGTCCTGTGTTCTACTGCCCCATCGCTAGCCATTCGAAGCTCAATATTTACT
CTGCCCCACAAAGAGGCGGGAAAGCCAAAGACGTGTTCGTGGCCCCAAACAAACAGACTGTCAACAGTTT
AAAGTTTTCATTGGGGCGAGCCATCATTGTTATCACTACTTAAATGGACCAGGATGAGGATCCATTTGTG
ATTGAGCGACGTCGCAATCGGCTGCGCGTGGAGACAGTTAGAGCGCAGCTCAATGCTCTTCTGGCACAAC
CCATCTTCCCCAGAGGCTTCAGCTTCAAGTATCCCAACAGCGAGGCGGCCCAATTAGCCACGAGTCATAC
TCAAAGTGCCGTGGAGACCATGAAGGCGGCCATAGAGGACCAGAAGCAGGCCAAAAAAGATCGCAACAAG
CGAAAACGGAATGCCTAATCTAACTTAAAGCTGTTTTATTGAATAGGAGATACAAAGCTATAGCCTTGGA
TGTTTCCATCAATGCTTTGAAACAAAGTATCGCACACCTCCATCGGTCAATGAGCGAGCGTGCTTGGCCA
ATAGTGTGGAAACGGACAAGTTTGGGGTGGTAAAGCACCTAGTTATAAAAACGAAAATCAAATCAAAAAC
AATCTAGATATGTGTTCATCAATACTTACTCTATGTACGGTTCCATTTCCTCCATCGTCCATCTCTTCTT
AGTCTTAAATAGCGCCCGCATGCGATCGCTTATATTGGTGGGCAGTAACTCCTCCGCCAGGGAACGTATG
CAAGGCTGCGCTCCCTCCTTGTCGCAAATGCCCAGGCCACGTAGATACTTTAAGTCGCAGGACATGCCCT
CGGGCAGTGCCTCCTGCCAGGTGCGCATGAACTCCTCGTTGCGAAAGCGCAAACCCGGCTGAAGAATGTT
CTGTGCCACAATGCGGGAGACCAAGGACTCCTGGTACTCAAACTTGCCCG

>In(3L)P
GCTGGTTTAATGGCTTCGACTGTATCAGACGCTGGATTTCTTCCAGATCCTGGGATAGAGTGGGTGGTGG
AGCCAAGGTAGCTGGCGGGCCCGGCGCCAACAGACCAACAGTTGGAGGCACCAACTGAGGAGACCACAGT
GCCAGGGATCCACTGGCCAGGGTAATCAGGAGAAGCCAATCCAGAGCGATGGCCATATCTGTAAGGTGGT
GACCGAAAGAAGACTAACAAAATTAAAGTCGCAGAAGCTTTTATATCCATCCATCAAGATTTGTTATTAC
TGTCTACTGATAGCGTCTTCCTGAATTTGGCGCAGCGTTTCTGTTGACCATTGTCGTCATCTGATAAGCG
CTTTTCGATCTGTTTATGGGTCTACCCAATTTTAAATCCCCCACGGAAGTCAGAGTTTTGGGATAAACTG
GCGGGTTGGTAAACATTTGGAGAGAAAGTATGCGAATTTATAACATGCTAAGCTTTATTTTCCAAGTGAA
TCACCAACAATCCGGATGGACCACATAGAACCGCAAACGAACACTTAATAACATTAGGATTTACATAGCT
CCTCATACATACACAAAGCAAACCCAAACTGGGGATTTATAGGATTCATCTTTTTTCGATTTTAAGAGAT
GATGACAAGTTAGTTATCCTTGCGAAATGGGCTTTGGAAAACGGATGATCGCAGTTAATGTTAATGAAAG
TTATGTCTTAGGTGGGCAGTGGCCACCACACTCGGTCAGTTGGCCATTTGGGCATTTGCCGAGTTGTGGG
GGGAGTGGGGTGTGCTGGTATTTTCTGGCCAAAAAGCTGGCCGAAGACAAAGCGAATCAGC

>In(3R)K_proximal
CTCATTTACCCGTGGTCAGCTAAAATGTCACCCATGGCAATGGTAACAGAGAAAAGCTCAAAGCTGGTCA
TAAACTTTATACTTGTTCATACTGTATGTATGAGACGTATGAATTGCAGAGTTGAGTGAATATTTAAATT
CAACAGTTGGGCATGCAAATTGCTCAGAATTCTCAAATATTTGCTTTTAATTTCCATGTCATGCATATTT
TGTGCAGCATTTAAATGAATGCATTTAAATGATTGCAACAGTTCCTTAAAATTTTGAAATGACTCACTTA
ACTAAGCTTAACTACAAGGCCAGGCCAATACGGTTTGGCACTGATTAAAATTTTGTTAAAAACTTTTAAG
TCCTGATGCGATCTACATTATGTGAAAAAATAAATAAAAGTTTTTATGGTTATGCAAAAAATGGGACACT
GGAGATGATTATCGGTTTGTTTGTCTGTGCCGCTTTTTCAGTTTTTTATATCCAACTCCGAGCCATAACA
AAACAATTTTGTTTACCCTCTGTTGTCTGACCCACTCTCCACTTGGATACTCAAACAGGCGACGCTGAAA
ACAGCCCGATGCCTCGGGGAAATCTCTGGGAAACTAGGAAAATGTCGGAACGGGATGTAATAGCTGGCTG
AGCTGAATGAATTTGCCCCATTTATCCACTAGAAACCAATCGAGATTGTTTAGCCGGCTTCTCCCAATTG
TAATGAAGAAAACTCGAGAATCGTTTTCAACATGGAAAAATGCGTACTTGTGGTTTTCGTCCTGATTGTG
GTAATGGGAAGAGTATATCTCTATGAAAGTGTCTTTTTCTTTGACCTTCTTTCGTGTATGCCC

>In(X)Be
GCTGCGTCTACTTGCTGTTGTTGTTGTTGATTTTCATCGGCTTTAATGCCTTTGTCTATTTTGGGTTTCT
GTTTATGGCGTGGGTTTTTCTCAATTGCATTTTTGGGCGCGCTCTCGACTGCAGCAGCAAAAGCACAGTG
GCGGGCAGAGAGATAGCAGCGTTTAAGAAATTAAGAAATATGCATATAGAAAATCGCCTAAAAACTGAAA
GAATTTAATACCCTAAAGAGGTGCTGTTTGATTCATTTTTAAATGTATAAATTGGATACGCTTTGCTATT
ATTTTGTCCTATTTTTCATCTTTAACGTGATACACATTGATTATATTAGTTCTAGACCATGAAATTCTCA
AATTTTAAAGACGTACGCTTTAAATGTACCATGTTAATTAGTTATTCTCTGGCAAAATGTAACAAAATTG
GAAACTACCATATTGACGGGCATTGTAAGTGTCGTATGTGTGAGTGTGGAAATACCAGAATTACCTAAAT
AACAACAAGAATAGCCGTGCACTTATAAAGCAATGTTTGTAATCGGATCGCATTGGCATGTTGGCCAGTT
GAGTTCAGTTCTCAATTGCTGGCCAAAAGTTTTTGGCCCCTCGAGTGTCTGACTGTGGACACGTTCTTCT
TTTTGAGTGGCCTCCTGATGGTGGTGATAGCTCTAAGAGCAATGGAAAGGTACGACTCTTTGGACTTAAG
CAACATGCACGGTGTCATTTAGACGGGTTTTGGAAACAAAGATTATTACTTCCTATTGACCCATTTGCAG
AACCAAAGGAAAGCTCAATGTGTCCCTGA
