# Supplementary material for: Global Diversity Lines–A Five-Continent Reference Panel of Sequenced Drosophila melanogaster Strains
Source: G3 (Bethesda). 2015 Feb 11;5(4):593–603. doi: 10.1534/g3.114.015883 (PMC4390575; doi:10.1534/g3.114.015883)
Supplement: Supporting Information [file supp_g3.114.015883_TableS1.pdf]

Table S1 Data Files

Contents	File Type	File Name(s)
Mapped reads	bam	<line>_GDL_WGS.bam*
Variant sites and genotypes	vcf	GDL_Chr<#>_SNPs.vcf*<br>GDL_Chr<#>_Indels.vcf*
Uncallable genome intervals	bed	GDL_uncallable_intervals.bed*
Regions IBD between populations	bed	GDL_IBD_regions.bed*
Heterozygous blocks	bed	GDL_HetBlocks.bed*
Multi-species alignment	MAF	view at http://genome-mirror.cshl.edu/

* SRA #SRP050151
