# Supplementary material for: Global Diversity Lines–A Five-Continent Reference Panel of Sequenced Drosophila melanogaster Strains
Source: G3 (Bethesda). 2015 Feb 11;5(4):593–603. doi: 10.1534/g3.114.015883 (PMC4390575; doi:10.1534/g3.114.015883)
Supplement: Supporting Information [file supp_g3.114.015883_TableS2.pdf]

Table S2 Read Counts by Line and by Chromosome

	X		2L		2R		3L		3R		4		Other	unmapped	
Line	Mapped Reads	*Avg DoC*	Mapped Reads	*Avg DoC*	Mapped Reads	*Avg DoC*	Mapped Reads	*Avg DoC*	Mapped Reads	*Avg DoC*	Mapped Reads	*Avg DoC*	Mapped Reads	# Reads	All reads
**Beijing, China**															
B04	3,036,688	*13.5*	3,294,733	*14.2*	3,020,949	*14.1*	3,469,105	*14.0*	4,012,220	*14.2*	212,663	*15.7*	3,690,254	1,725,808	22,462,420
B05	2,975,969	*13.2*	3,199,230	*13.8*	3,017,967	*14.1*	3,391,466	*13.7*	3,955,448	*14.0*	190,030	*14.0*	3,696,277	1,802,635	22,229,022
B10	3,006,384	*13.3*	3,249,286	*14.0*	2,992,388	*14.0*	3,420,409	*13.8*	3,958,669	*14.0*	201,815	*14.9*	4,363,567	1,583,002	22,775,520
B11	2,874,138	*12.7*	3,102,107	*13.3*	2,881,317	*13.5*	3,273,001	*13.2*	3,799,545	*13.5*	178,789	*13.2*	3,467,321	947,860	20,524,078
B12	3,117,248	*13.8*	3,387,218	*14.6*	3,107,037	*14.5*	3,588,896	*14.5*	4,128,763	*14.7*	215,253	*15.9*	3,988,871	1,042,874	22,576,160
B14	2,866,674	*12.7*	3,100,647	*13.3*	2,903,760	*13.6*	3,263,524	*13.2*	3,815,092	*13.5*	172,172	*12.7*	3,821,257	992,134	20,935,260
B23	2,680,424	*11.9*	2,885,414	*12.4*	2,703,854	*12.7*	3,052,229	*12.3*	3,565,008	*12.6*	163,975	*12.1*	2,938,173	1,500,197	19,489,274
B28	2,969,053	*13.1*	3,182,360	*13.7*	2,978,186	*13.9*	3,361,246	*13.6*	3,956,731	*14.0*	191,390	*14.1*	3,543,061	1,613,391	21,795,418
B38	2,967,684	*13.1*	3,188,412	*13.7*	2,985,929	*14.0*	3,376,369	*13.6*	3,911,689	*13.9*	185,567	*13.7*	3,511,111	1,002,299	21,129,060
B42	2,888,902	*12.8*	3,095,855	*13.3*	2,914,346	*13.6*	3,288,477	*13.3*	3,821,217	*13.6*	175,070	*12.9*	3,451,671	1,300,870	20,936,408
B43	2,836,306	*12.6*	3,050,177	*13.1*	2,857,044	*13.4*	3,231,038	*13.0*	3,754,890	*13.3*	173,324	*12.8*	3,407,559	1,351,016	20,661,354
B51	2,718,890	*12.0*	2,909,627	*12.5*	2,709,346	*12.7*	3,080,824	*12.4*	3,563,731	*12.6*	162,530	*12.0*	3,464,387	1,470,791	20,080,126
B52	2,980,418	*13.2*	3,201,375	*13.8*	3,039,420	*14.2*	3,400,459	*13.7*	3,979,801	*14.1*	170,940	*12.6*	3,250,693	1,045,958	21,069,064
B54	3,081,805	*13.7*	3,311,988	*14.3*	3,064,437	*14.4*	3,522,141	*14.2*	4,054,104	*14.4*	211,039	*15.6*	4,056,469	1,066,665	22,368,648
B59	2,297,710	*10.2*	2,658,126	*11.5*	2,250,237	*10.6*	2,774,998	*11.2*	3,013,778	*10.7*	219,460	*16.2*	3,974,267	321,486	17,510,062
**Ithaca, NY; North America**															
I01	3,226,159	*14.3*	3,508,949	*15.1*	3,214,396	*15.0*	3,713,192	*15.0*	4,260,913	*15.1*	250,279	*18.5*	4,353,981	2,120,995	24,648,864
I02	3,250,803	*14.4*	3,518,042	*15.1*	3,174,403	*14.9*	3,751,124	*15.1*	4,229,956	*15.0*	224,964	*16.6*	5,098,103	1,359,359	24,606,754
I03	3,337,483	*14.8*	3,622,081	*15.6*	3,286,862	*15.4*	3,833,662	*15.5*	4,370,088	*15.5*	233,148	*17.2*	4,186,246	1,638,022	24,507,592
I04	2,414,568	*10.7*	2,613,311	*11.2*	2,411,398	*11.3*	2,747,824	*11.1*	3,195,403	*11.3*	147,092	*10.8*	3,039,884	1,586,796	18,156,276
I06	2,296,416	*10.2*	2,683,927	*11.6*	2,312,681	*10.9*	2,795,719	*11.3*	3,079,207	*11.0*	191,818	*14.2*	3,429,044	1,300,110	18,088,922
I07	2,953,381	*13.1*	3,194,076	*13.7*	2,973,494	*13.9*	3,369,990	*13.6*	3,920,546	*13.9*	178,112	*13.1*	3,698,312	1,606,341	21,894,252
I13	3,341,466	*14.8*	3,614,755	*15.6*	3,274,832	*15.3*	3,826,004	*15.4*	4,364,808	*15.5*	233,200	*17.2*	4,884,146	2,490,161	26,029,372
I16	2,188,470	*9.7*	2,515,281	*10.9*	2,148,538	*10.1*	2,653,293	*10.7*	2,877,838	*10.3*	195,850	*14.5*	3,537,036	882,012	16,998,318
I17	3,480,184	*15.4*	3,771,246	*16.2*	3,381,971	*15.8*	3,978,544	*16.0*	4,500,611	*16.0*	251,956	*18.6*	4,348,931	2,175,781	25,889,224
I22	3,078,020	*13.6*	3,334,031	*14.3*	3,086,353	*14.4*	3,525,120	*14.2*	4,071,864	*14.5*	200,487	*14.8*	3,727,401	1,825,552	22,848,828
I23	3,400,043	*15.1*	3,681,958	*15.8*	3,378,539	*15.8*	3,914,561	*15.8*	4,500,820	*16.0*	220,345	*16.2*	3,971,172	1,726,272	24,793,710
I24	3,051,144	*13.5*	3,279,481	*14.1*	3,069,162	*14.4*	3,470,800	*14.0*	4,005,999	*14.2*	183,884	*13.5*	3,794,857	1,454,109	22,309,436
I26	2,336,786	*10.4*	2,691,791	*11.6*	2,344,720	*11.0*	2,796,824	*11.3*	3,125,576	*11.1*	189,769	*14.0*	3,421,484	320,876	17,227,826
I29	3,476,520	*15.4*	3,782,781	*16.3*	3,509,444	*16.4*	3,990,293	*16.1*	4,611,204	*16.4*	223,300	*16.4*	3,599,386	1,130,154	24,323,082
I31	2,685,643	*11.9*	2,864,483	*12.3*	2,683,780	*12.6*	3,015,219	*12.2*	3,520,315	*12.5*	159,047	*11.7*	3,065,260	1,450,553	19,444,300
I33	2,631,419	*11.7*	2,805,240	*12.1*	2,654,000	*12.4*	2,978,415	*12.0*	3,476,429	*12.3*	155,529	*11.5*	2,808,188	807,922	18,317,142

Table S2 Read Counts by Line and by Chromosome *cont.*

	X		2L		2R		3L		3R		4		Other	Unmapped	
Line	Mapped Reads	*Avg DoC*	Mapped Reads	*Avg DoC*	Mapped Reads	*Avg DoC*	Mapped Reads	*Avg DoC*	Mapped Reads	*Avg DoC*	Mapped Reads	*Avg DoC*	Mapped Reads	# Reads	All reads
**Ithaca, NY; North America** *cont.*															
I34	2,605,581	*11.5*	2,798,179	*12.0*	2,631,284	*12.3*	2,957,573	*11.9*	3,473,913	*12.3*	149,338	*11.0*	3,103,483	1,429,141	19,148,492
I35	2,650,204	*11.8*	2,889,464	*12.4*	2,688,141	*12.6*	3,038,748	*12.3*	3,541,389	*12.6*	163,575	*12.0*	3,050,198	1,098,427	19,120,146
I38	3,046,933	*13.5*	3,274,762	*14.1*	3,065,928	*14.3*	3,461,098	*14.0*	4,060,443	*14.4*	180,612	*13.3*	3,419,112	924,514	21,433,402
**Netherlands, Europe**															
N01	2,672,621	*11.9*	2,879,436	*12.4*	2,585,067	*12.1*	3,057,652	*12.3*	3,451,466	*12.3*	188,812	*13.9*	3,150,412	1,231,938	19,217,404
N02	2,885,047	*12.8*	3,099,663	*13.4*	2,793,423	*13.1*	3,280,875	*13.3*	3,719,541	*13.2*	201,465	*14.8*	3,417,667	1,246,813	20,644,494
N03	2,643,251	*11.7*	2,836,132	*12.2*	2,549,983	*11.9*	3,029,124	*12.2*	3,403,174	*12.1*	179,145	*13.2*	3,342,778	1,643,029	19,626,616
N04	2,714,598	*12.0*	2,906,524	*12.5*	2,616,973	*12.3*	3,115,407	*12.6*	3,504,246	*12.5*	188,258	*13.9*	3,241,832	1,581,186	19,869,024
N07	2,614,345	*11.6*	2,794,792	*12.0*	2,526,219	*11.8*	2,972,442	*12.0*	3,377,459	*12.0*	183,773	*13.5*	3,306,395	2,623,703	20,399,128
N10	2,762,048	*12.2*	2,966,830	*12.8*	2,671,331	*12.5*	3,141,768	*12.7*	3,567,745	*12.7*	194,015	*14.3*	3,365,200	2,774,081	21,443,018
N11	2,561,775	*11.4*	2,739,439	*11.8*	2,455,598	*11.5*	2,910,080	*11.7*	3,275,421	*11.6*	177,038	*13.0*	3,008,986	2,430,285	19,558,622
N13	2,007,332	*8.9*	2,178,327	*9.4*	1,944,961	*9.1*	2,302,586	*9.3*	2,589,275	*9.2*	139,667	*10.3*	2,953,696	2,888,632	17,004,476
N14	2,556,803	*11.3*	2,778,505	*12.0*	2,493,331	*11.7*	2,960,652	*11.9*	3,324,314	*11.8*	179,060	*13.2*	3,346,798	2,598,831	20,238,294
N15	2,140,351	*9.5*	2,305,988	*9.9*	2,060,129	*9.6*	2,443,834	*9.9*	2,764,036	*9.8*	153,206	*11.3*	2,792,190	2,385,130	17,044,864
N16	2,657,923	*11.8*	2,879,808	*12.4*	2,590,886	*12.1*	3,025,148	*12.2*	3,467,536	*12.3*	196,403	*14.5*	3,510,830	2,694,080	21,022,614
N17	2,720,721	*12.1*	2,912,528	*12.5*	2,633,445	*12.3*	3,104,319	*12.5*	3,538,345	*12.6*	190,261	*14.0*	3,546,928	1,188,533	19,835,080
N18	2,706,424	*12.0*	2,860,701	*12.3*	2,572,061	*12.1*	3,041,970	*12.3*	3,435,187	*12.2*	193,772	*14.3*	3,509,259	1,133,088	19,452,462
N19	2,545,409	*11.3*	2,721,587	*11.7*	2,472,318	*11.6*	2,918,159	*11.8*	3,292,900	*11.7*	178,014	*13.1*	3,378,758	1,801,793	19,308,938
N22	2,594,926	*11.5*	2,763,158	*11.9*	2,527,215	*11.8*	2,960,637	*12.0*	3,371,385	*12.0*	181,036	*13.3*	3,638,679	1,377,154	19,414,190
N23	2,680,173	*11.9*	2,872,055	*12.4*	2,592,838	*12.1*	3,072,860	*12.4*	3,463,906	*12.3*	191,237	*14.1*	3,806,792	1,297,673	19,977,534
N25	2,483,169	*11.0*	2,653,241	*11.4*	2,380,018	*11.2*	2,814,972	*11.4*	3,182,260	*11.3*	170,859	*12.6*	2,986,125	1,695,424	18,366,068
N29	2,394,677	*10.6*	2,560,181	*11.0*	2,313,070	*10.8*	2,736,354	*11.1*	3,096,088	*11.0*	171,944	*12.7*	2,879,955	876,081	17,028,350
N30	2,601,721	*11.5*	2,810,593	*12.1*	2,524,842	*11.8*	2,962,690	*12.0*	3,343,543	*11.9*	185,376	*13.7*	3,098,593	1,311,580	18,838,938
**Tasmania, Australia**															
T01	2,609,342	*11.6*	2,806,398	*12.1*	2,538,444	*11.9*	2,963,810	*12.0*	3,377,590	*12.0*	187,856	*13.8*	3,187,477	1,596,685	19,267,602
T04	2,695,615	*11.9*	2,944,937	*12.7*	2,642,729	*12.4*	3,143,618	*12.7*	3,525,917	*12.5*	203,238	*15.0*	3,247,738	2,074,542	20,478,334
T05	2,212,527	*9.8*	2,393,906	*10.3*	2,163,829	*10.1*	2,536,623	*10.2*	2,890,343	*10.2*	155,773	*11.4*	2,921,670	2,073,635	17,348,306
T07	2,730,524	*12.1*	2,949,876	*12.7*	2,662,556	*12.4*	3,135,924	*12.6*	3,559,569	*12.6*	197,232	*14.5*	3,791,203	1,892,646	20,919,530
T09	2,614,884	*11.6*	2,836,286	*12.2*	2,575,365	*12.1*	3,007,487	*12.1*	3,433,549	*12.2*	191,110	*14.1*	3,399,748	1,592,453	19,650,882
T10	2,694,315	*11.9*	2,879,062	*12.4*	2,612,489	*12.2*	3,073,461	*12.4*	3,497,154	*12.4*	183,994	*13.5*	3,323,085	1,997,484	20,261,044
T14A	2,824,044	*12.5*	3,028,138	*13.0*	2,748,202	*12.8*	3,230,063	*13.0*	3,659,572	*13.0*	191,699	*14.1*	3,814,940	2,365,782	21,862,440
T22A	2,413,591	*10.7*	2,608,848	*11.2*	2,350,302	*11.0*	2,757,057	*11.1*	3,145,979	*11.1*	172,482	*12.6*	2,861,882	2,157,661	18,467,802
T23	2,084,131	*9.2*	2,255,754	*9.7*	2,026,514	*9.5*	2,399,362	*9.7*	2,720,426	*9.7*	138,360	*10.2*	2,615,391	2,707,958	16,947,896
T24	2,541,912	*11.3*	2,717,973	*11.7*	2,465,687	*11.5*	2,889,107	*11.7*	3,274,862	*11.6*	176,156	*13.0*	3,260,393	1,429,964	18,756,054
T25A	2,423,463	*10.7*	2,608,506	*11.2*	2,355,569	*11.0*	2,763,855	*11.1*	3,138,458	*11.1*	168,925	*12.4*	2,966,167	1,311,455	17,736,398

Table S2 Read Counts by Line and by Chromosome *cont.*

	X		2L		2R		3L		3R		4		Other	Unmapped	
Line	Mapped Reads	*Avg DoC*	Mapped Reads	*Avg DoC*	Mapped Reads	*Avg DoC*	Mapped Reads	*Avg DoC*	Mapped Reads	*Avg DoC*	Mapped Reads	*Avg DoC*	Mapped Reads	# Reads	All reads
**Tasmania, Australia** *cont.*															
T29A	2,726,239	*12.1*	2,934,365	*12.6*	2,632,961	*12.3*	3,106,046	*12.5*	3,539,226	*12.6*	193,836	*14.3*	3,228,284	2,408,727	20,769,684
T30A	2,750,016	*12.2*	2,970,764	*12.8*	2,668,337	*12.5*	3,133,105	*12.6*	3,558,955	*12.6*	193,316	*14.2*	3,331,261	1,208,494	19,814,248
T35	2,488,591	*11.0*	2,685,736	*11.6*	2,416,500	*11.3*	2,850,237	*11.5*	3,231,543	*11.5*	177,540	*13.1*	3,733,324	1,465,527	19,048,998
T36B	2,743,172	*12.2*	2,965,658	*12.8*	2,670,885	*12.5*	3,151,340	*12.7*	3,545,922	*12.6*	196,666	*14.5*	3,273,345	1,543,780	20,090,768
T39	2,725,523	*12.1*	2,924,030	*12.6*	2,637,324	*12.3*	3,111,731	*12.6*	3,523,461	*12.5*	190,848	*14.1*	3,437,056	1,411,785	19,961,758
T43A	2,154,291	*9.5*	2,317,445	*10.0*	2,078,454	*9.7*	2,459,230	*9.9*	2,775,729	*9.8*	152,223	*11.2*	2,621,449	2,902,111	17,460,932
T45B	2,330,496	*10.3*	2,529,899	*10.9*	2,337,976	*10.9*	2,693,901	*10.9*	3,090,043	*11.0*	149,005	*11.0*	2,992,793	1,560,437	17,684,550
**Zimbabwe, Africa**															
ZH23	2,575,459	*11.4*	2,766,549	*11.9*	2,533,300	*11.8*	2,959,179	*11.9*	3,383,858	*12.0*	188,712	*13.9*	3,439,165	1,804,756	19,650,978
ZH26	2,589,418	*11.4*	2,837,108	*12.2*	2,563,883	*12.0*	3,003,891	*12.1*	3,409,195	*12.1*	189,569	*13.9*	3,725,403	2,194,373	20,512,840
ZH33	2,649,472	*11.7*	2,856,989	*12.3*	2,727,304	*12.8*	3,031,176	*12.2*	3,579,097	*12.7*	151,893	*11.2*	3,130,759	1,104,706	19,231,396
ZH42	2,972,582	*13.2*	3,242,876	*13.9*	3,017,990	*14.1*	3,428,694	*13.8*	3,983,790	*14.1*	184,733	*13.6*	3,508,896	1,233,925	21,573,486
ZS10	2,559,281	*11.3*	2,790,297	*12.0*	2,603,958	*12.2*	2,946,016	*11.9*	3,430,725	*12.2*	159,830	*11.8*	3,152,941	1,067,284	18,710,332
ZW09	2,659,983	*11.8*	2,939,827	*12.6*	2,696,341	*12.6*	3,095,530	*12.5*	3,580,265	*12.7*	176,672	*13.0*	3,181,496	1,258,388	19,588,502
ZW139	2,729,548	*12.1*	3,024,063	*13.0*	2,795,696	*13.1*	3,222,144	*13.0*	3,708,748	*13.1*	177,553	*13.1*	3,033,429	1,308,819	20,000,000
ZW140	2,737,445	*12.1*	3,018,753	*13.0*	2,771,506	*13.0*	3,211,076	*12.9*	3,697,881	*13.1*	177,059	*13.0*	3,170,461	1,552,769	20,336,950
ZW142	2,751,245	*12.2*	3,015,481	*13.0*	2,758,944	*12.9*	3,212,675	*13.0*	3,692,034	*13.1*	181,030	*13.3*	3,415,925	972,666	20,000,000
ZW144	2,574,503	*11.4*	2,814,264	*12.1*	2,673,337	*12.5*	2,971,541	*12.0*	3,514,858	*12.5*	141,552	*10.4*	2,652,985	1,081,288	18,424,328
ZW155	2,693,520	*11.9*	2,959,209	*12.7*	2,721,907	*12.7*	3,130,876	*12.6*	3,619,366	*12.8*	172,586	*12.7*	3,013,541	1,878,607	20,189,612
ZW177	2,682,984	*11.9*	2,941,812	*12.6*	2,721,627	*12.7*	3,121,164	*12.6*	3,577,353	*12.7*	177,947	*13.1*	3,269,893	1,770,160	20,262,940
ZW184	2,730,523	*12.1*	3,001,890	*12.9*	2,806,159	*13.2*	3,158,435	*12.8*	3,673,716	*13.1*	162,684	*12.0*	3,122,647	1,721,408	20,377,462
ZW185	2,677,725	*11.8*	2,906,698	*12.5*	2,740,756	*12.8*	3,070,819	*12.4*	3,607,400	*12.8*	168,127	*12.4*	2,912,457	1,390,146	19,474,128
