# Supplementary material for: Global Diversity Lines–A Five-Continent Reference Panel of Sequenced Drosophila melanogaster Strains
Source: G3 (Bethesda). 2015 Feb 11;5(4):593–603. doi: 10.1534/g3.114.015883 (PMC4390575; doi:10.1534/g3.114.015883)
Supplement: Supporting Information [file supp_g3.114.015883_TableS3.pdf]

Table S3 Variant Calls by Line and Chromosome

	X		2L		2R		3L		3R		4	
**Line**	**SNP**	***indel***	**SNP**	***indel***	**SNP**	***indel***	**SNP**	***indel***	**SNP**	***indel***	**SNP**	***Indel***
**Beijing, China**												
B04	96,318	*15,163*	140,046	*16,680*	123,435	*14,886*	139,281	*17,262*	129,823	*16,793*	825	*151*
B05	95,028	*13,580*	139,781	*15,351*	122,472	*13,782*	184,280	*23,033*	231,795	*36,114*	972	*139*
B10	96,583	*14,577*	139,105	*16,331*	124,632	*14,634*	140,353	*17,013*	177,103	*25,965*	1,031	*140*
B11	95,292	*12,667*	232,757	*29,217*	122,749	*12,780*	157,161	*19,884*	149,582	*19,042*	1,006	*114*
B12	96,931	*16,690*	246,150	*36,044*	123,565	*15,796*	138,549	*18,681*	140,716	*19,204*	911	*164*
B14	96,301	*12,623*	135,733	*13,748*	120,988	*12,608*	211,642	*30,884*	133,540	*15,284*	960	*116*
B23	93,915	*10,864*	138,564	*12,614*	122,560	*11,525*	225,754	*31,648*	210,687	*29,811*	907	*102*
B28	93,806	*12,479*	136,764	*14,247*	124,054	*13,005*	137,809	*14,556*	169,706	*25,171*	1,355	*147*
B38	95,178	*13,547*	135,447	*14,608*	122,236	*13,470*	202,217	*29,036*	188,075	*25,737*	930	*144*
B42	94,959	*12,785*	139,773	*14,230*	119,990	*12,852*	211,057	*29,178*	222,683	*30,807*	965	*148*
B43	95,024	*13,013*	137,936	*14,379*	118,903	*12,615*	213,921	*31,549*	200,852	*30,459*	974	*108*
B51	96,095	*11,967*	139,731	*12,931*	121,593	*11,616*	214,229	*30,990*	226,099	*31,692*	1,077	*138*
B52	94,071	*13,040*	137,891	*14,637*	124,429	*13,773*	220,342	*33,634*	192,926	*30,074*	892	*131*
B54	94,300	*15,674*	241,173	*33,728*	140,194	*19,329*	140,953	*18,656*	131,375	*17,647*	811	*139*
B59	86,094	*10,758*	200,582	*24,230*	145,049	*17,271*	134,297	*14,567*	147,570	*18,390*	897	*156*
**Ithaca, NY; North America**												
I01	88,664	*15,306*	249,227	*36,836*	189,734	*26,653*	144,946	*19,608*	155,496	*22,870*	913	*156*
I02	84,777	*15,814*	144,695	*19,048*	124,388	*16,335*	145,946	*21,597*	131,528	*18,608*	1,490	*321*
I03	84,162	*15,804*	143,713	*19,679*	120,766	*16,658*	141,783	*21,769*	187,773	*33,602*	1,624	*324*
I04	84,050	*7,852*	238,388	*26,262*	123,078	*9,474*	144,657	*10,613*	137,724	*10,518*	1,749	*176*
I06	82,358	*10,372*	237,733	*32,024*	127,834	*13,201*	155,295	*19,345*	131,383	*13,424*	1,324	*211*
I07	87,118	*11,914*	153,777	*17,297*	140,445	*17,193*	143,051	*15,811*	130,871	*15,312*	646	*91*
I13	85,410	*15,973*	156,026	*21,284*	132,284	*18,184*	142,227	*20,899*	134,053	*19,408*	775	*158*
I16	83,851	*10,177*	152,314	*15,977*	125,186	*11,469*	138,317	*14,306*	133,596	*12,813*	597	*119*
I17	84,606	*17,490*	146,275	*21,586*	126,100	*18,204*	165,328	*28,785*	211,233	*34,725*	1,883	*381*
I22	83,199	*13,527*	238,724	*33,614*	139,837	*18,175*	155,382	*21,570*	136,559	*17,555*	1,739	*261*
I23	85,522	*15,824*	177,750	*25,895*	137,867	*19,900*	181,574	*32,124*	171,608	*30,330*	1,745	*309*
I24	84,033	*12,846*	144,222	*16,064*	122,238	*13,860*	152,145	*19,237*	228,070	*35,924*	1,755	*258*
I26	82,050	*10,364*	171,419	*19,891*	132,505	*14,893*	147,016	*16,357*	218,586	*31,455*	1,753	*285*
I29	80,354	*16,008*	152,322	*22,276*	127,821	*18,993*	148,117	*23,215*	141,891	*22,349*	1,758	*336*
I31	85,686	*9,491*	147,215	*13,061*	121,298	*10,815*	139,516	*12,305*	133,632	*12,361*	623	*85*
I33	85,856	*8,722*	145,161	*12,128*	123,325	*10,357*	140,330	*11,427*	135,450	*12,108*	774	*77*
I34	84,360	*8,586*	145,356	*11,711*	123,620	*10,562*	138,855	*11,298*	132,567	*12,013*	1,749	*138*
I35	85,892	*10,011*	145,090	*13,108*	135,593	*14,410*	149,116	*15,333*	141,055	*14,825*	1,571	*212*
I38	84,257	*12,565*	144,793	*16,263*	126,387	*14,638*	140,211	*16,710*	132,167	*16,504*	376	*44*
**Netherlands, Europe**												
N01	84,069	*11,700*	135,973	*13,886*	118,272	*11,584*	136,113	*14,884*	130,973	*14,050*	1,053	*175*
N02	82,245	*12,656*	168,631	*21,948*	127,955	*15,404*	148,378	*19,396*	126,962	*15,498*	1,411	*258*
N03	81,964	*11,087*	149,558	*16,299*	128,168	*13,039*	137,960	*14,661*	131,503	*13,508*	1,751	*284*
N04	81,134	*11,783*	133,540	*14,019*	119,473	*12,324*	138,218	*15,596*	128,168	*14,230*	998	*155*
N07	81,946	*9,800*	246,489	*31,202*	123,149	*11,124*	135,300	*12,981*	130,656	*12,574*	1,801	*298*
N10	77,723	*9,766*	243,257	*33,545*	121,777	*11,897*	136,496	*13,899*	120,623	*11,468*	990	*153*
N11	83,581	*9,272*	140,051	*11,616*	121,243	*9,563*	144,441	*14,302*	210,892	*27,181*	809	*123*
N13	84,220	*4,947*	132,988	*5,999*	121,860	*5,407*	148,311	*9,046*	199,752	*20,535*	774	*86*
N14	84,593	*9,790*	152,629	*15,504*	172,495	*19,528*	137,079	*12,694*	130,165	*11,543*	1,332	*203*

Table S3 Variant Calls by Line and Chromosome *cont.*

	**X**		**2L**		**2R**		**3L**		**3R**		**4**	
**Line**	**SNP**	***indel***	**SNP**	***indel***	**SNP**	***indel***	**SNP**	***indel***	**SNP**	***indel***	**SNP**	***Indel***
**Netherlands, Europe** *cont.*												
N15	83,057	*5,568*	133,749	*7,117*	119,336	*6,022*	141,062	*7,777*	130,485	*7,068*	1,031	*124*
N16	82,112	*10,205*	138,845	*14,043*	125,061	*12,141*	179,650	*22,051*	134,816	*13,746*	1,345	*220*
N17	81,425	*11,925*	243,849	*32,932*	128,610	*14,260*	161,846	*22,448*	135,199	*16,049*	1,828	*304*
N18	83,648	*11,477*	144,991	*15,536*	122,774	*12,124*	140,244	*15,194*	130,026	*13,852*	1,343	*231*
N19	82,514	*10,083*	191,707	*16,895*	120,820	*10,827*	139,118	*14,282*	143,492	*15,257*	814	*121*
N22	81,720	*11,059*	195,296	*17,918*	120,232	*11,334*	139,630	*14,285*	151,230	*19,237*	1,423	*230*
N23	80,426	*11,353*	239,092	*31,771*	132,963	*14,816*	137,440	*15,303*	130,077	*13,672*	1,444	*272*
N25	80,597	*9,298*	142,909	*13,581*	130,935	*12,652*	141,292	*13,627*	139,295	*13,858*	1,501	*213*
N29	83,842	*9,376*	232,943	*28,224*	126,212	*11,076*	138,198	*12,380*	131,252	*11,722*	1,023	*146*
N30	82,121	*11,022*	136,796	*13,046*	122,019	*11,717*	138,389	*14,423*	131,620	*13,112*	599	*96*
**Tasmania, Australia**												
T01	88,545	*11,392*	145,059	*14,143*	122,432	*11,745*	151,573	*15,887*	136,473	*14,146*	1,103	*173*
T04	86,848	*11,101*	142,673	*13,912*	126,325	*12,060*	224,878	*33,979*	220,759	*31,879*	791	*126*
T05	87,228	*7,063*	144,979	*9,173*	124,532	*7,592*	155,479	*11,684*	140,118	*9,476*	1,438	*196*
T07	86,441	*11,323*	251,811	*34,275*	195,476	*26,509*	145,557	*15,383*	138,655	*14,549*	1,806	*306*
T09	86,890	*10,866*	158,923	*15,885*	128,359	*12,492*	218,547	*30,102*	143,221	*14,744*	1,163	*204*
T10	87,170	*10,909*	145,668	*14,084*	125,080	*11,760*	226,060	*34,057*	143,116	*15,564*	1,329	*212*
T14A	85,205	*11,828*	150,028	*15,715*	124,610	*12,898*	157,477	*19,788*	147,875	*17,411*	1,320	*236*
T22A	87,191	*8,733*	141,364	*10,596*	175,803	*18,811*	144,421	*11,516*	139,996	*10,908*	1,998	*312*
T23	86,362	*5,907*	138,913	*8,270*	129,441	*7,433*	130,381	*7,042*	115,406	*5,876*	564	*63*
T24	87,618	*10,481*	148,354	*13,305*	123,271	*10,754*	151,850	*15,397*	217,293	*29,344*	771	*126*
T25A	87,383	*9,420*	243,607	*29,635*	180,890	*20,908*	145,664	*12,846*	170,346	*14,275*	1,456	*209*
T29A	86,144	*11,723*	148,499	*14,880*	145,675	*13,984*	169,758	*17,687*	135,739	*14,168*	1,743	*287*
T30	87,253	*12,997*	148,957	*15,922*	125,375	*12,949*	217,955	*32,672*	219,363	*32,895*	1,777	*310*
T35	88,072	*10,311*	230,647	*27,284*	191,412	*23,869*	177,848	*16,575*	139,212	*12,766*	1,753	*240*
T36B	86,850	*12,201*	158,913	*18,006*	139,412	*16,064*	228,587	*35,919*	210,741	*30,101*	1,462	*271*
T39	86,450	*12,058*	147,384	*14,957*	144,614	*14,283*	147,689	*16,784*	192,150	*24,956*	803	*138*
T43A	87,807	*6,382*	152,276	*8,213*	176,200	*16,618*	151,405	*9,296*	195,388	*19,437*	985	*108*
T45B	88,761	*7,603*	151,777	*10,567*	122,916	*8,584*	146,190	*11,022*	217,157	*26,461*	1,508	*132*
**Zimbabwe, Africa**												
ZH23	171,276	*19,994*	204,521	*18,998*	146,959	*13,563*	203,705	*21,230*	253,696	*35,044*	1,720	*286*
ZH26	145,667	*14,911*	255,846	*28,755*	118,051	*10,202*	250,722	*33,502*	250,140	*33,002*	1,624	*249*
ZH33	153,164	*16,046*	220,937	*18,399*	160,023	*14,369*	257,568	*32,256*	235,131	*31,661*	1,657	*138*
ZH42	177,872	*26,159*	303,456	*39,819*	165,397	*20,274*	285,542	*42,409*	277,232	*42,613*	1,692	*261*
ZS10	173,895	*18,068*	296,140	*33,740*	223,932	*25,838*	201,038	*18,420*	271,514	*34,181*	1,663	*158*
ZW09	184,492	*22,328*	310,275	*37,906*	169,181	*18,243*	268,603	*35,164*	269,233	*34,219*	1,852	*266*
ZW139	183,555	*22,781*	220,721	*21,472*	178,604	*18,201*	288,917	*40,851*	285,369	*39,726*	1,881	*243*
ZW140	182,107	*22,612*	224,337	*22,359*	173,788	*17,491*	289,355	*40,001*	285,229	*38,391*	1,796	*247*
ZW142	185,220	*24,332*	211,945	*22,398*	173,675	*18,481*	282,061	*38,771*	274,969	*37,558*	1,718	*267*
ZW144	183,630	*19,141*	222,452	*19,041*	170,538	*15,981*	281,612	*34,034*	281,479	*35,442*	1,684	*139*
ZW155	175,922	*20,577*	217,761	*20,034*	167,308	*15,895*	283,317	*39,027*	210,343	*23,406*	1,620	*210*
ZW177	185,795	*22,245*	226,623	*22,010*	172,763	*17,103*	286,148	*38,347*	284,316	*37,910*	1,750	*237*
ZW184	153,937	*17,836*	150,410	*14,736*	118,980	*12,130*	141,061	*14,189*	134,517	*14,362*	608	*82*
ZW185	181,366	*19,617*	305,132	*35,499*	174,455	*17,357*	255,225	*30,194*	250,399	*29,334*	1,693	*193*
