# Supplementary material for: Global Diversity Lines–A Five-Continent Reference Panel of Sequenced Drosophila melanogaster Strains
Source: G3 (Bethesda). 2015 Feb 11;5(4):593–603. doi: 10.1534/g3.114.015883 (PMC4390575; doi:10.1534/g3.114.015883)
Supplement: Supporting Information [file supp_g3.114.015883_TableS4.pdf]

Table S4 Genotypes of Known Large Inversions

Chrom	2L	2R	3L	3L	3R	3R	3R	3R	X	X
Inv Name	In(2L)t	In(2R)NS	In(3L)P	In(3L) 62D:68A	In(3R)K	In(3R)P	In(3R)Mo	In(3R) 13-72	X(Be)	X(A)
Ref.	*1*	*2*	*2*	*3*	*2*	*4*	*2*	*3*	*2*	*2*
**Beijing, China**										
B04	0/0	0/0	0/0	0/0	0/0	0/0	0/0	0/0	0/0	0/0
B05	0/0	0/0	**1/1**	0/0	0/0	**0/1**	0/0	0/0	0/0	0/0
B10	0/0	0/0	0/0	0/0	0/0	0/0	0/0	0/0	0/0	0/0
B11	**0/1**	0/0	0/0	0/0	0/0	0/0	0/0	0/0	0/0	0/0
B12	**0/1**	0/0	0/0	0/0	0/0	0/0	0/0	0/0	0/0	0/0
B14	0/0	0/0	**0/1**	0/0	0/0	0/0	0/0	0/0	0/0	0/0
B23	0/0	0/0	**0/1**	0/0	0/0	**0/1**	0/0	0/0	0/0	0/0
B28	0/0	0/0	0/0	0/0	0/0	0/0	**0/1**	0/0	0/0	0/0
B38	0/0	0/0	**0/1**	0/0	0/0	0/0	0/0	0/0	0/0	0/0
B42	0/0	0/0	**0/1**	0/0	0/0	**0/1**	0/0	0/0	0/0	0/0
B43	0/0	0/0	**0/1**	0/0	0/0	0/0	0/0	0/0	0/0	0/0
B51	0/0	0/0	**0/1**	0/0	0/0	**0/1**	0/0	0/0	0/0	0/0
B52	0/0	0/0	**0/1**	0/0	0/0	0/0	0/0	0/0	0/0	0/0
B54	**0/1**	0/0	0/0	0/0	0/0	0/0	0/0	0/0	0/0	0/0
B59	**0/1**	0/0	0/0	0/0	0/0	0/0	0/0	0/0	0/0	0/0
**Ithaca, NY; North America**										
I01	**0/1**	**0/1**	0/0	0/0	0/0	0/0	**0/1**	0/0	0/0	0/0
I02	0/0	0/0	0/0	0/0	0/0	0/0	**1/1**	0/0	0/0	0/0
I03	0/0	0/0	0/0	0/0	0/0	0/0	**0/1**	0/0	0/0	0/0
I04	**0/1**	0/0	0/0	0/0	0/0	0/0	0/0	0/0	0/0	0/0
I06	**0/1**	0/0	0/0	0/0	0/0	0/0	0/0	0/0	0/0	0/0
I07	0/0	0/0	0/0	0/0	0/0	0/0	0/0	0/0	0/0	0/0
I13	0/0	0/0	0/0	0/0	0/0	0/0	0/0	0/0	0/0	0/0
I16	0/0	0/0	0/0	0/0	0/0	0/0	0/0	0/0	0/0	0/0
I17	0/0	0/0	0/0	0/0	**0/1**	0/0	0/0	0/0	0/0	0/0
I22	**0/1**	0/0	0/0	0/0	0/0	0/0	0/0	**1/1**	0/0	0/0
I23	0/0	0/0	0/0	0/0	0/0	0/0	0/0	0/0	0/0	0/0
I24	0/0	0/0	0/0	0/0	0/0	**0/1**	0/0	0/0	0/0	0/0
I26	0/0	0/0	0/0	0/0	**0/1**	0/0	0/0	0/0	0/0	0/0
I29	0/0	0/0	0/0	0/0	0/0	0/0	0/0	0/0	0/0	0/0
I31	0/0	0/0	0/0	0/0	0/0	0/0	0/0	0/0	0/0	0/0
I33	0/0	0/0	0/0	0/0	0/0	0/0	0/0	0/0	0/0	0/0
I34	0/0	0/0	0/0	0/0	0/0	0/0	0/0	0/0	0/0	0/0
I35	0/0	0/0	0/0	0/0	0/0	0/0	0/0	0/0	0/0	0/0
I38	0/0	0/0	0/0	0/0	0/0	0/0	0/0	0/0	0/0	0/0
**Netherlands, Europe**										
N01	0/0	0/0	0/0	0/0	0/0	0/0	0/0	0/0	0/0	0/0
N02	0/0	0/0	0/0	0/0	0/0	0/0	0/0	0/0	0/0	0/0
N03	0/0	0/0	0/0	0/0	0/0	0/0	0/0	0/0	0/0	0/0
N04	0/0	0/0	0/0	0/0	0/0	0/0	0/0	0/0	0/0	0/0
N07	**0/1**	0/0	0/0	0/0	0/0	0/0	0/0	0/0	0/0	0/0
N10	**0/1**	0/0	0/0	0/0	0/0	0/0	0/0	0/0	0/0	0/0
N11	0/0	0/0	0/0	0/0	0/0	**0/1**	0/0	0/0	0/0	0/0
N13	0/0	0/0	0/0	0/0	0/0	0/0	0/0	0/0	0/0	0/0
N14	0/0	0/0	0/0	0/0	0/0	0/0	0/0	0/0	0/0	0/0

Table S4 Genotype of Known Large Inversions *cont.*

Chrom	2L	2R	3L	3L	3R	3R	3R	3R	X	X
Inv Name	In(2L)t	In(2R)NS	In(3L)P	In(3L) 62D:68A	In(3R)K	In(3R)P	In(3R)Mo	In(3R) 13-72	X(Be)	X(A)
Ref.	*1*	*2*	*2*	*3*	*2*	*4*	*2*	*3*	*2*	*2*
**Netherlands, Europe** *cont.*										
N15	0/0	0/0	0/0	0/0	0/0	0/0	0/0	0/0	0/0	0/0
N16	0/0	0/0	**0/1**	0/0	0/0	0/0	0/0	0/0	0/0	0/0
N17	**0/1**	0/0	0/0	0/0	0/0	0/0	0/0	0/0	0/0	0/0
N18	0/0	0/0	0/0	0/0	0/0	0/0	**1/1**	0/0	0/0	0/0
N19	**1/1**	0/0	0/0	0/0	0/0	0/0	0/0	0/0	0/0	0/0
N22	**1/1**	0/0	0/0	0/0	0/0	0/0	**0/1**	0/0	0/0	0/0
N23	**0/1**	0/0	0/0	0/0	0/0	0/0	0/0	0/0	0/0	0/0
N25	0/0	0/0	0/0	0/0	0/0	0/0	0/0	0/0	0/0	0/0
N29	**0/1**	0/0	0/0	0/0	0/0	0/0	0/0	0/0	0/0	0/0
N30	0/0	0/0	0/0	0/0	0/0	0/0	0/0	0/0	0/0	0/0
**Tasmania, Australia**										
T01	0/0	0/0	0/0	0/0	0/0	0/0	0/0	0/0	0/0	0/0
T04	0/0	0/0	**0/1**	0/0	0/0	**0/1**	0/0	0/0	0/0	0/0
T05	0/0	0/0	0/0	0/0	0/0	0/0	0/0	0/0	0/0	0/0
T07	**0/1**	**0/1**	0/0	0/0	0/0	0/0	0/0	0/0	0/0	0/0
T09	0/0	0/0	**0/1**	0/0	0/0	0/0	0/0	0/0	0/0	0/0
T10	0/0	0/0	**0/1**	0/0	0/0	0/0	0/0	0/0	0/0	0/0
T14A	0/0	0/0	0/0	0/0	0/0	0/0	0/0	0/0	0/0	0/0
T22A	0/0	**0/1**	0/0	0/0	0/0	0/0	0/0	0/0	0/0	0/0
T23	0/0	0/0	0/0	0/0	0/0	0/0	0/0	0/0	0/0	0/0
T24	0/0	0/0	0/0	0/0	0/0	**0/1**	0/0	0/0	0/0	0/0
T25A	**0/1**	**0/1**	0/0	0/0	0/0	0/0	0/0	0/0	0/0	0/0
T29A	0/0	**0/1**	**1/1**	0/0	0/0	0/0	0/0	0/0	0/0	0/0
T30A	0/0	0/0	**0/1**	0/0	0/0	**0/1**	0/0	0/0	0/0	0/0
T35	0/0	**0/1**	**1/1**	0/0	0/0	0/0	0/0	0/0	0/0	0/0
T36B	0/0	0/0	**0/1**	0/0	0/0	0/0	0/0	0/0	0/0	0/0
T39	0/0	**0/1**	0/0	0/0	0/0	0/0	0/0	0/0	0/0	0/0
T43A	0/0	**0/1**	0/0	0/0	0/0	**0/1**	0/0	0/0	0/0	0/0
T45B	0/0	0/0	0/0	0/0	0/0	**0/1**	0/0	0/0	0/0	0/0
**Zimbabwe, Africa**										
ZH23	**1/1**	0/0	0/0	0/0	0/0	0/0	**0/1**	0/0	**1/1**	0/0
ZH26	**0/1**	0/0	0/0	0/0	**0/1**	**0/1**	0/0	0/0	0/0	**0/1**
ZH33	0/0	0/0	0/0	**0/1**	**0/1**	0/0	0/0	0/0	0/0	0/0
ZH42	**0/1**	0/0	0/0	**0/1**	0/0	**0/1**	0/0	0/0	0/0	0/0
ZS10	**0/1**	**0/1**	0/0	0/0	**0/1**	0/0	0/0	0/0	0/0	0/0
ZW09	**0/1**	0/0	0/0	**0/1**	0/0	**0/1**	0/0	0/0	0/0	0/0
ZW139	0/0	0/0	0/0	**0/1**	0/0	**0/1**	0/0	0/0	0/0	0/0
ZW140	0/0	0/0	0/0	**0/1**	0/0	**0/1**	0/0	0/0	0/0	0/0
ZW142	0/0	0/0	0/0	**0/1**	0/0	**0/1**	0/0	0/0	0/0	0/0
ZW144	0/0	0/0	0/0	**0/1**	0/0	**0/1**	0/0	0/0	0/0	0/0
ZW155	0/0	0/0	0/0	**0/1**	0/0	0/0	0/0	0/0	0/0	0/0
ZW177	0/0	0/0	0/0	**0/1**	0/0	**0/1**	0/0	0/0	0/0	0/0
ZW184	0/0	0/0	0/0	0/0	0/0	0/0	0/0	0/0	0/0	0/0
ZW185	**0/1**	0/0	0/0	**0/1**	0/0	**0/1**	0/0	0/0	0/0	0/0

1. Andolfatto *et al.* 1999 2. Corbett-Detig *et al.* 2012 3. This study 4. Sezgin *et al.* 2004
