# Supplementary material for: Global Diversity Lines–A Five-Continent Reference Panel of Sequenced Drosophila melanogaster Strains
Source: G3 (Bethesda). 2015 Feb 11;5(4):593–603. doi: 10.1534/g3.114.015883 (PMC4390575; doi:10.1534/g3.114.015883)
Supplement: Supporting Information [file supp_g3.114.015883_FigureS1.pdf]

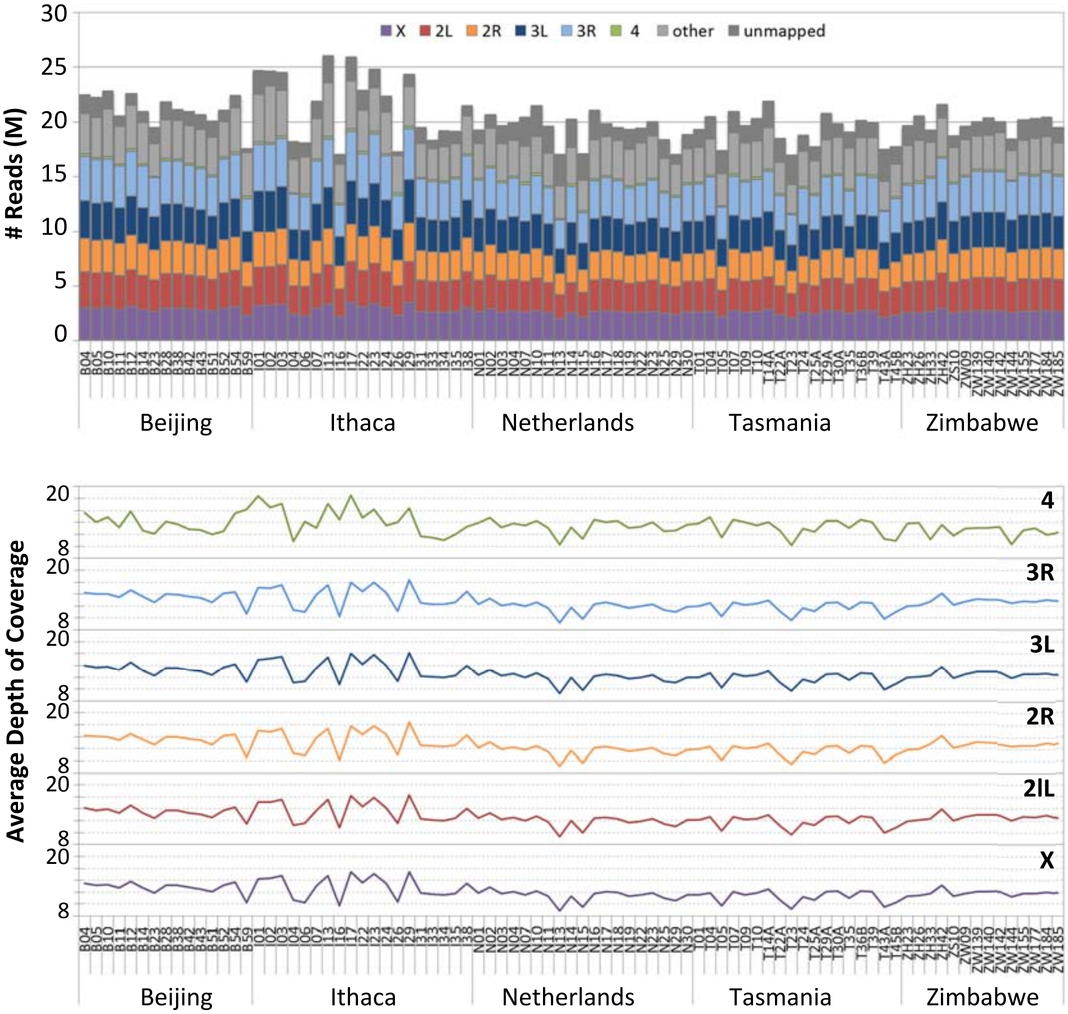

Figure S1 Read Counts and Depth of Coverage by Chromosome
(**Top**) The number of reads mapped to the reference genome is shown for each *Drosophila* line. Contigs other than the 5 major chromosome arms (X, 2L, 2R, 3L, 3R) and chromosome 4 are grouped into the 'other' category. Unmapped reads constituted 8% of the total reads, on average. (**Bottom**) The average depth of coverage is shown for each line, for each of the 5 major chromosome arms (X, 2L, 2R, 3L, 3R) and chromosome 4, with most lines having average depth > 12 for most chromosomes.
