# Supplementary material for: Global Diversity Lines–A Five-Continent Reference Panel of Sequenced Drosophila melanogaster Strains
Source: G3 (Bethesda). 2015 Feb 11;5(4):593–603. doi: 10.1534/g3.114.015883 (PMC4390575; doi:10.1534/g3.114.015883)
Supplement: Supporting Information [file supp_g3.114.015883_FigureS2.pdf]

ddRAD Validation (12 lines)					
		ddRAD Genotype			
		REF	HET	ALT	*# Calls*
GATK	REF	**99.9%**	0.1%	0%	*333,312*
	HET	25%	**62%**	13%	*13,992*
	ALT	0.1%	0.2%	**99.7%**	*40,245*

100x Validation (ZW155)					
		100x Genotype			
		REF	HET	ALT	*# Calls*
GATK	REF	**99.7%**	0.3%	0%	*3.8M*
	HET	11%	**81%**	8%	*308,299*
	High	*5%*	***93%***	*2%*	*248,046*
	Low	*37%*	***30%***	*33%*	*60,253*
	ALT	0%	0.7%	**99.2%**	*572,859*

Figure S2 Summary of SNP Validation

SNPs were validated using two independent strategies. (**Top**) For 12 lines, genotypes called from ddRAD libraries covering ~1% of the genome matched the WGS SNP genotypes called by the GATK pipeline for >99% of homozygous genotypes. Heterozygous genotype calls from the GATK pipeline validated at a lower rate (62%) likely due to underrepresentation of both chromosome copies in the ddRAD libraries (Arnold *et al.* 2013; Davey *et al.* 2013; Gautier *et al.* 2013). (**Bottom**) For one line, ZW155, independent libraries were constructed and sequenced to 100x depth. Genotypes were called based on read depth per allele (see Methods) and compared to the genotypes called by the GATK pipeline from the ZW155 10x library. Again, calls matched for >99% of homozygous genotypes, but the validation rate was lower for heterozygous calls (81%). For the 100x dataset with full genome coverage, there were sufficient heterozygous calls to divide these into sites that fall in regions with *high* vs *low* heterozygous call frequency for this line. The validation rate was considerably higher in regions of high heterozygosity compared to regions of low heterozygosity, consistent with these being true regions of residual heterozygosity.
