# Supplementary material for: Global Diversity Lines–A Five-Continent Reference Panel of Sequenced Drosophila melanogaster Strains
Source: G3 (Bethesda). 2015 Feb 11;5(4):593–603. doi: 10.1534/g3.114.015883 (PMC4390575; doi:10.1534/g3.114.015883)
Supplement: Supporting Information [file supp_g3.114.015883_FigureS3.pdf]

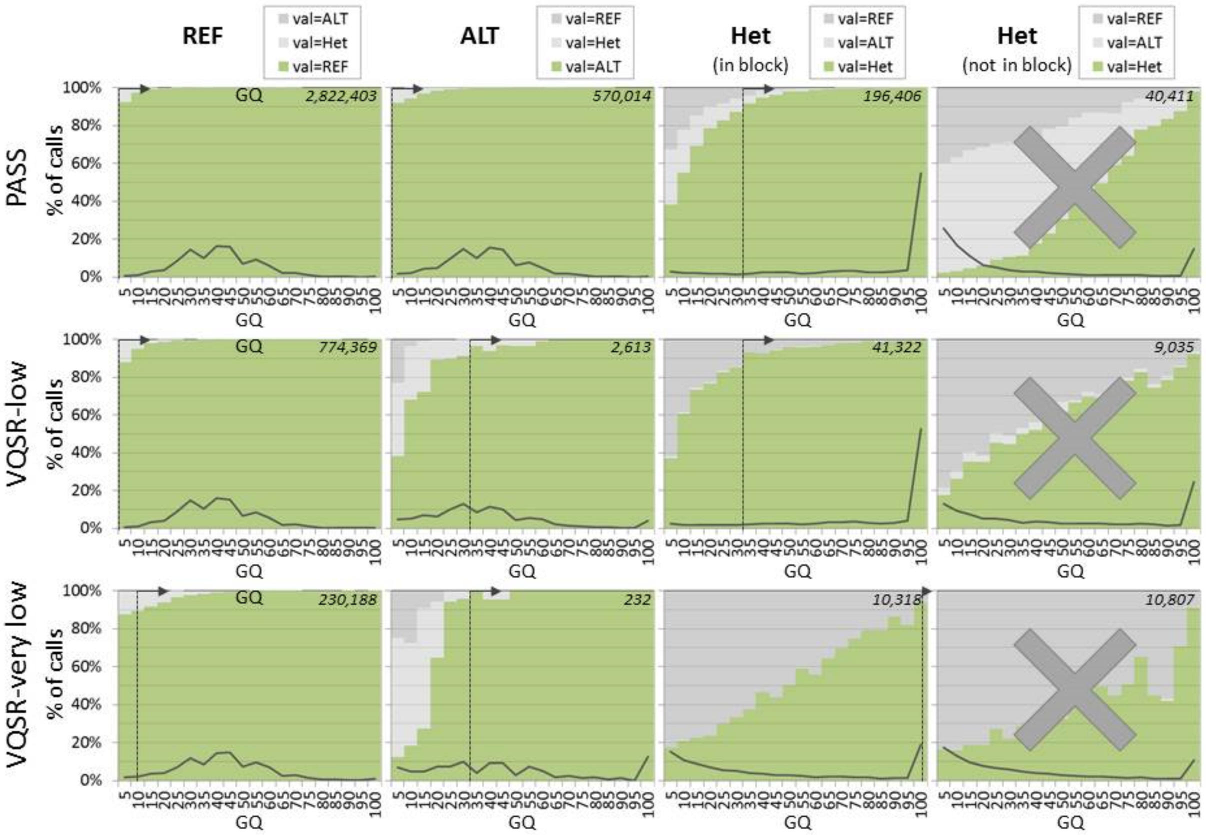

Figure S3 SNP Validation Rate Correlates with Genotype Quality Score
Validation rates were determined for different classes of GATK SNP calls, divided by Variant Quality Recalibration flag (rows: PASS, VQSR-low, VQSR-very low), SNP genotype (columns: REF, ALT, Het-high freq, Het-low freq), and Genotype Quality score (x-axes: GQ) for line ZW155 compared to the 100x validation dataset (val). Green bars indicate the genotypic agreement for each GQ bin; grey bars indicate discordant calls. The relative frequency of each GQ bin for each class of genotype call is shown as a dark grey line, with heterozygous calls having a bimodal GQ distribution. Vertical black lines and arrows indicate the GQ cutoff for each class of genotype call used to filter the SNP dataset. All heterozygous calls in regions of low heterozygous call frequency (assessed per line) were filtered out of the final SNP genotypes.
