# Supplementary material for: Global Diversity Lines–A Five-Continent Reference Panel of Sequenced Drosophila melanogaster Strains
Source: G3 (Bethesda). 2015 Feb 11;5(4):593–603. doi: 10.1534/g3.114.015883 (PMC4390575; doi:10.1534/g3.114.015883)
Supplement: Supporting Information [file supp_g3.114.015883_FigureS5.pdf]

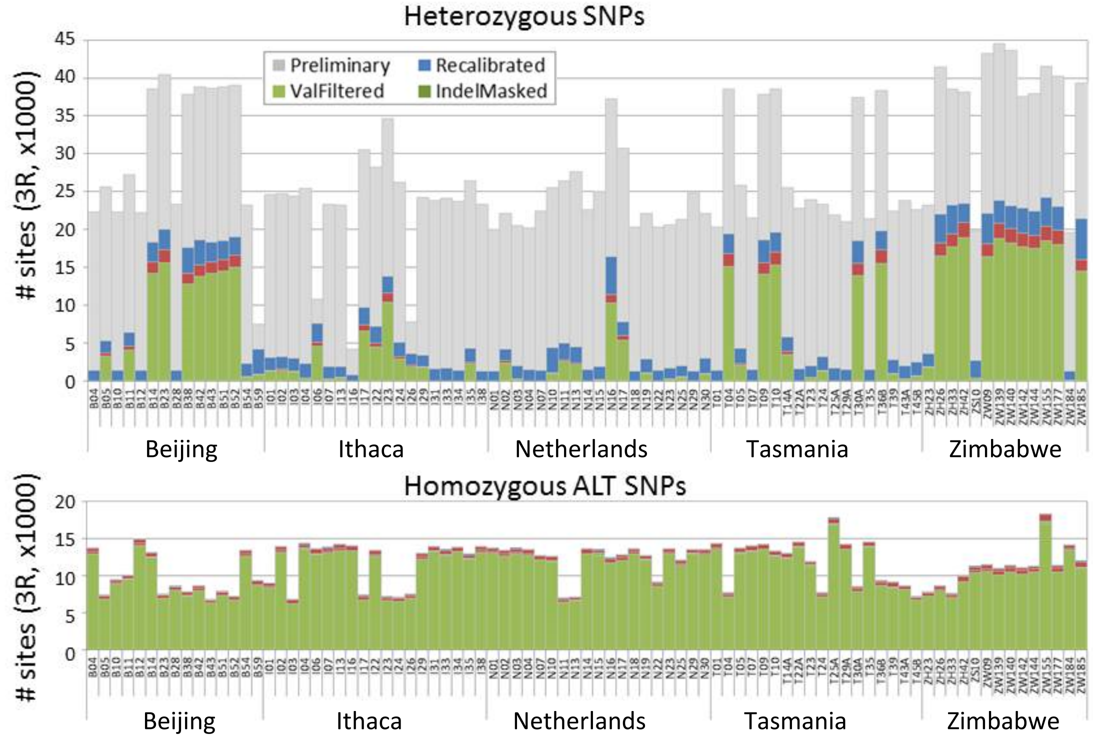

Figure S5 SNP Genotype Counts Per Line

The number of heterozygous (top) and homozygous ALT (bottom) SNP genotype per line is shown for a representative chromosome arm (3R), for each step in the SNP calling pipeline. The most significant change in SNP calls occurred at the Base Quality recalibration step for heterozygous calls, where the number of heterozygous calls dropped by 20,000 for most lines. After Base Quality recalibration, some lines (primarily from the Beijing and Zimbabwe populations) still had a high number of heterozygous calls. The SNP calling pipeline had much less effect on the homozygous ALT calls.
