# Supplementary material for: Global Diversity Lines–A Five-Continent Reference Panel of Sequenced Drosophila melanogaster Strains
Source: G3 (Bethesda). 2015 Feb 11;5(4):593–603. doi: 10.1534/g3.114.015883 (PMC4390575; doi:10.1534/g3.114.015883)
Supplement: Supporting Information [file supp_g3.114.015883_FigureS7.pdf]

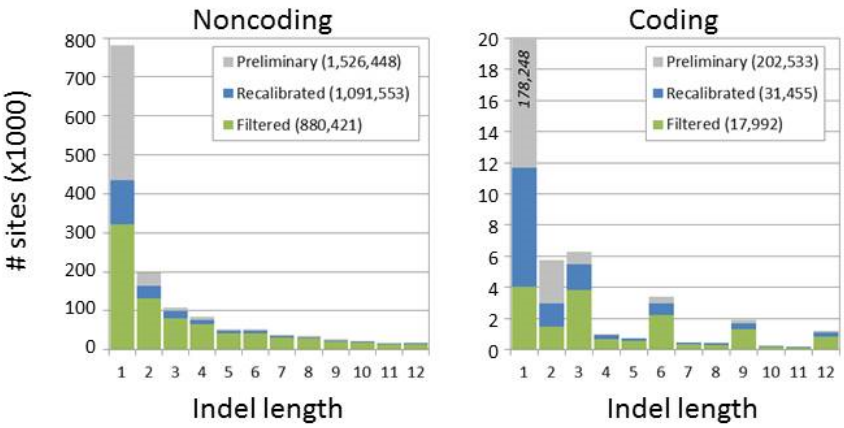

Figure S7 Small Indel Length Distribution

The number of 1-nt and 2-nt small indel variants dropped as calls were improved through the GATK pipeline, although the number of small indels in non-coding regions (left) were less affected than small indel calls within coding regions (right). The largest effect was a >90% reduction in the number of 1-nt small indels called within coding regions at the Base Quality recalibration step. Within coding regions, the selective constraint of maintaining the protein reading frame is evident in the 3nt periodicity in the small indel length distribution.
