# Supplementary material for: Global Diversity Lines–A Five-Continent Reference Panel of Sequenced Drosophila melanogaster Strains
Source: G3 (Bethesda). 2015 Feb 11;5(4):593–603. doi: 10.1534/g3.114.015883 (PMC4390575; doi:10.1534/g3.114.015883)
Supplement: Supporting Information [file supp_g3.114.015883_FigureS8.pdf]

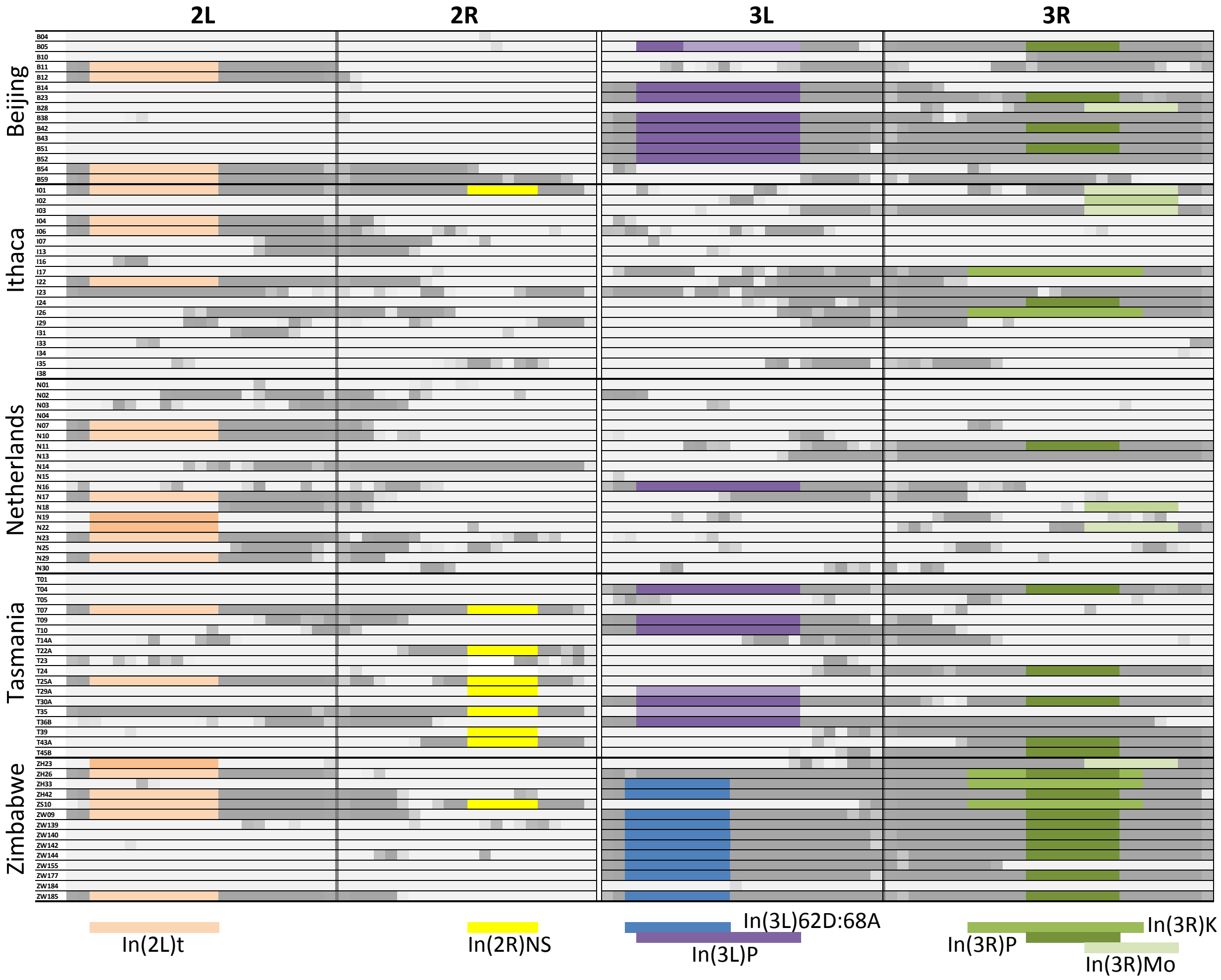

Figure S8 Large Known Inversions and Residual Heterozygosity
Regions with a high frequency of heterozygous genotype calls (‘heterozygous blocks’) are shown in grey for chromosomes 2 and 3 for each line. The lines from the Beijing and Zimbabwe populations have the highest frequency of heterozygous blocks, especially on chromosome 3. There is a clear correlation between the presence of residual heterozygosity and the presence of a heterozygous large inversion (colored bars). Overall, large inversions explain 57% of the observed heterozygous blocks.
