# Supplementary material for: Global Diversity Lines–A Five-Continent Reference Panel of Sequenced Drosophila melanogaster Strains
Source: G3 (Bethesda). 2015 Feb 11;5(4):593–603. doi: 10.1534/g3.114.015883 (PMC4390575; doi:10.1534/g3.114.015883)
Supplement: Supporting Information [file supp_g3.114.015883_FigureS9.pdf]

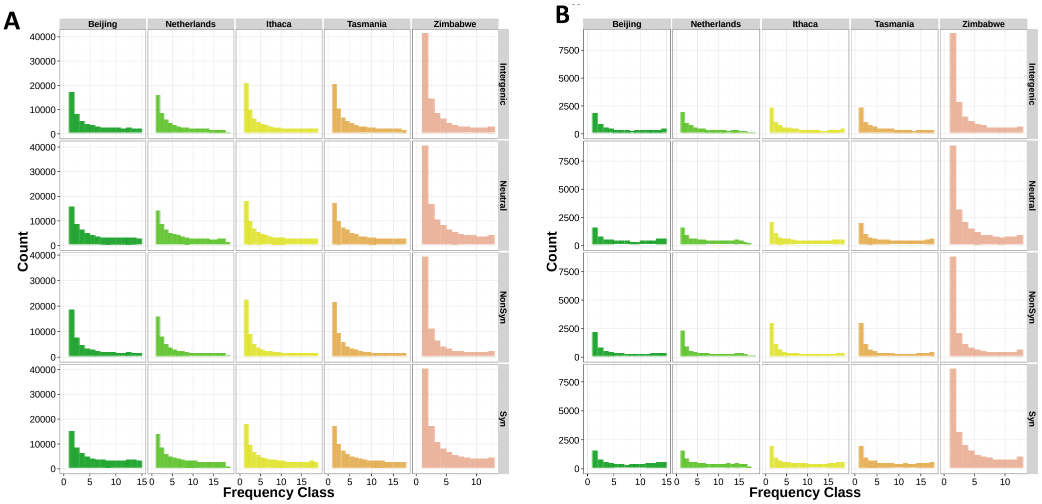


**Figure S9 Population-Specific Site Frequency Spectra for Four Classes of SNPs**
Unfolded SNP site frequency spectra (SFS) for four classes of SNPs separated by autosomes (**A**) and Chromosome X (**B**) for each population. Note that the X-axis varies between populations due to varying sample sizes. Most differences in SFS reside between the low frequency bins between the African sample and all non-African samples. There are is also a notable X-effect observed in the reduction of low frequency variants for the non-African samples. NonSyn = nonsynonymous sites; Syn = synonymous sites.
