# Supplementary material for: Global Diversity Lines–A Five-Continent Reference Panel of Sequenced Drosophila melanogaster Strains
Source: G3 (Bethesda). 2015 Feb 11;5(4):593–603. doi: 10.1534/g3.114.015883 (PMC4390575; doi:10.1534/g3.114.015883)
Supplement: Supporting Information [file supp_g3.114.015883_FigureS10.pdf]

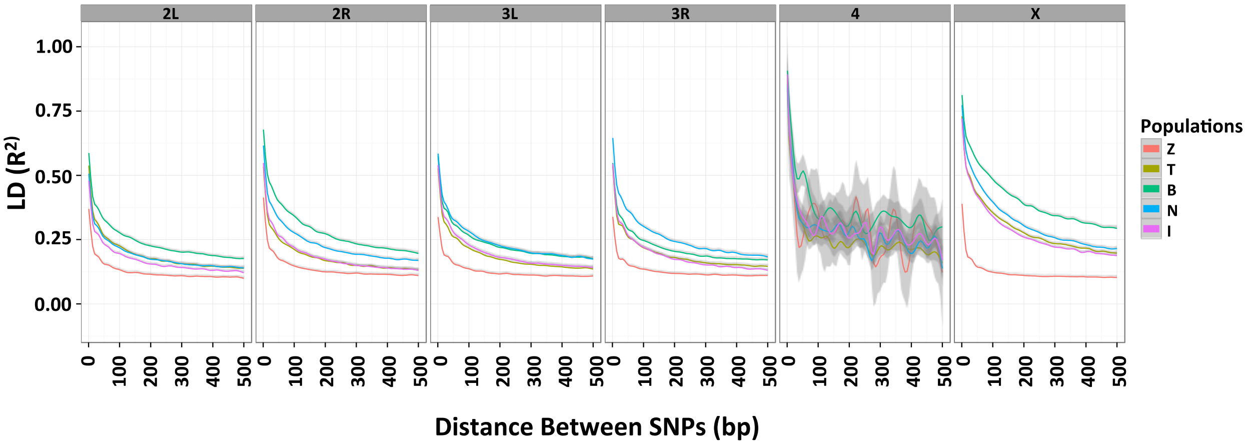

Figure S10 Decay of Linkage Disequilibrium Over Distance

Linkage disequilibrium as measured by R^2 divided by population and by chromosome. Most LD is quickly lost within the first 100-200 bp. The decay is most dramatic in the ancestral African population, particularly on chromosome X.
