# Supplementary material for: Global Diversity Lines–A Five-Continent Reference Panel of Sequenced Drosophila melanogaster Strains
Source: G3 (Bethesda). 2015 Feb 11;5(4):593–603. doi: 10.1534/g3.114.015883 (PMC4390575; doi:10.1534/g3.114.015883)
Supplement: Supporting Information [file supp_g3.114.015883_FigureS6.pdf]

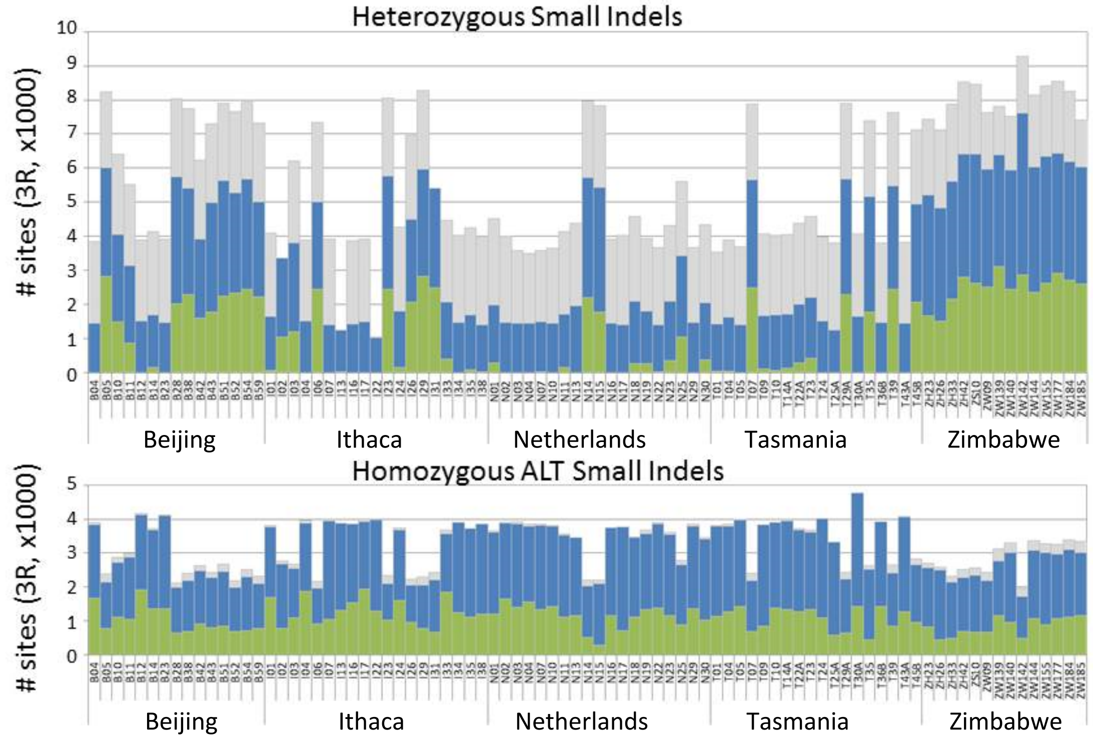

Figure S6 Small Indel Genotype Counts Per Line

The number of heterozygous (top) and homozygous ALT (bottom) small indel calls per line is shown for a representative chromosome arm (3R), for each step in the small indel calling pipeline. The two most significant change in small indel calls occurred at the Base Quality recalibration step for heterozygous calls, where the number of heterozygous calls dropped by 2,000 for most lines, and the Genotype Quality filtering step for all calls, which further reduced the number of small indel calls by 2,000 for most lines. After Base Quality recalibration, some lines (primarily from the Beijing and Zimbabwe populations) still had a high number of heterozygous calls, matching the same pattern seen for SNP calls (see Figure S5). Legend is the same as Figure S5.
